# Supplementary material for: Preclinical Efficacy and Involvement of AKT, mTOR, and ERK Kinases in the Mechanism of Sulforaphane against Endometrial Cancer
Source: Cancers (Basel). 2020 May 18;12(5):1273. doi: 10.3390/cancers12051273 (PMC7281543; doi:10.3390/cancers12051273)
Supplement: Supplementary file 1 [file cancers-12-01273-s001.zip › cancers-800856 supplementary/cancers-800856 supplementary.pdf]

cancers

Preclinical Efficacy and Involvement of AKT, mTOR, and ERK Kinases in the Mechanism of Sulforaphane against Endometrial Cancer

Rajani Rai [1,†], Kathleen Gong Essel [2,†], Doris Mangiaracina Benbrook [1,2], Justin Garland [1], Yan Daniel Zhao [3], and Vishal Chandra [1,2,*]

[1] Stephenson Cancer Center, University of Oklahoma Health Sciences Center, Oklahoma City, OK 73104, USA; rrai@ouhsc.edu (R.R.); Doris-Benbrook@ouhsc.edu (D.M.B.); Justin-Garland@ouhsc.edu (J.G.)
[2] Division of Gynecologic Oncology, Department of Obstetrics and Gynecology, College of Medicine, University of Oklahoma Health Sciences Center, Oklahoma City, OK 73104, USA; Kathleen-Essel@ouhsc.edu
[3] Biostatistics & Epidemiology, College of Public Health University of Oklahoma Health Sciences Center, Oklahoma City, OK 73104, USA, daniel-zhao@ouhsc.edu
* Correspondence: vishal-chandra@ouhsc.edu
† Co-First Authors.

Supplementary

A

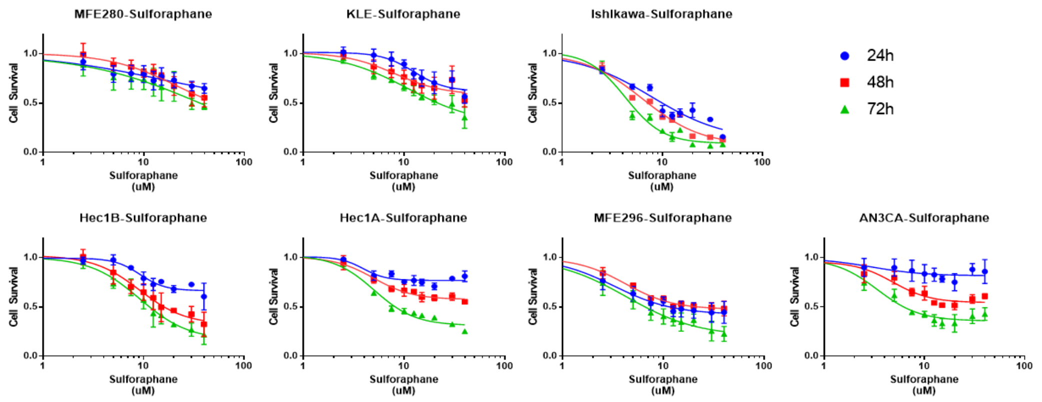

B

		MFE280	KLE	Ishikawa	Hec1B	Hec1A	MFE296	AN3CA
**24hrs**	CNT vs. 40 μM	****	****	****	****	****	****	***
	CNT vs. 30 μM	****	****	****	****	****	****	***
	CNT vs. 20 μM	****	****	****	****	****	****	****
	CNT vs. 15 μM	****	****	****	****	****	****	****
	CNT vs. 12.5 μM	****	****	****	****	****	****	****
	CNT vs. 10 μM	****	**	****	****	****	****	****
	CNT vs. 7.5 μM	****	ns	****	****	****	****	***
	CNT vs. 5 μM	****	ns	****	ns	****	****	*
	CNT vs. 2.5 μM	*	ns	****	ns	ns	****	*
**48hrs**	CNT vs. 40 μM	****	****	****	****	****	****	****
	CNT vs. 30 μM	****	****	****	****	****	****	****
	CNT vs. 20 μM	****	****	****	****	****	****	****
	CNT vs. 15 μM	****	****	****	****	****	****	****
	CNT vs. 12.5 μM	****	****	****	****	****	****	****
	CNT vs. 10 μM	****	****	****	****	****	****	****
	CNT vs. 7.5 μM	****	**	****	****	****	****	****
	CNT vs. 5 μM	**	ns	****	****	****	****	**
	CNT vs. 2.5 μM	ns	ns	****	ns	*	****	ns
**72hrs**	CNT vs. 40 μM	****	****	****	****	****	****	****
	CNT vs. 30 μM	****	****	****	****	****	****	****
	CNT vs. 20 μM	****	****	****	****	****	****	****
	CNT vs. 15 μM	****	****	****	****	****	****	****
	CNT vs. 12.5 μM	****	****	****	****	****	****	****
	CNT vs. 10 μM	****	****	****	****	****	****	****
	CNT vs. 7.5 μM	****	****	****	****	****	****	****
	CNT vs. 5 μM	****	****	****	****	****	****	****
	CNT vs. 2.5 μM	***	ns	****	ns	*	****	****

Figure S1. (**A**) Sulforaphane inhibits endometrial cancer cells viability. Multiple endometrial cancer cells (MFE280, KLE, Ishikawa, Hec1B, Hec1A, MFE296, and AN3CA) were treated with sulforaphane at the different μM (1, 2.5, 5, 7.5, 10, 12.5, 15, 20, 30, 40 μM) concentrations for 24, 48 and 72h and cellular viability was assessed

using the MTT assay. Data are the mean ± SD of three independent experiments performed in triplicate. **Figure S1b:**'p' values are indicated *; p ≤ 0.05, **; p≤0.01, ***; p≤0.001, ****; p≤0.0001 when compared with respective control. SFN; sulforaphane. (**B**) Sulforaphane inhibits endometrial cancer cells viability.

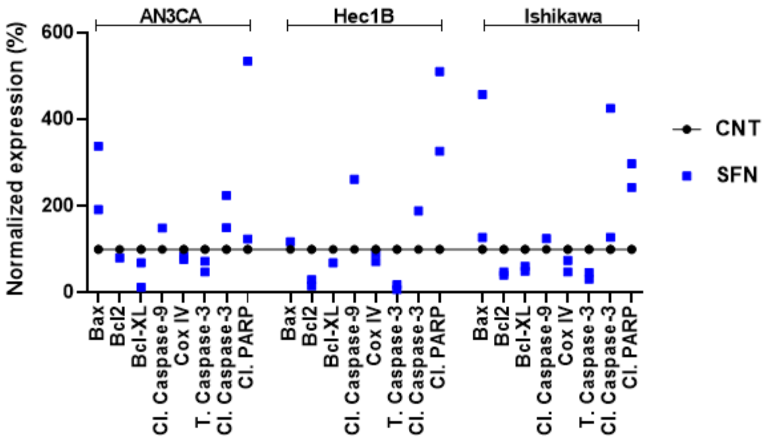

Figure S2. Densitometry analysis of Sulforaphane induced apoptotic markers. Densitometry analysis was performed for AN3CA and Hec1B cells treated with 12.5 µM of sulforaphane, and Ishikawa cell treated with 10 µM of sulforaphane. SFN; sulforaphane.

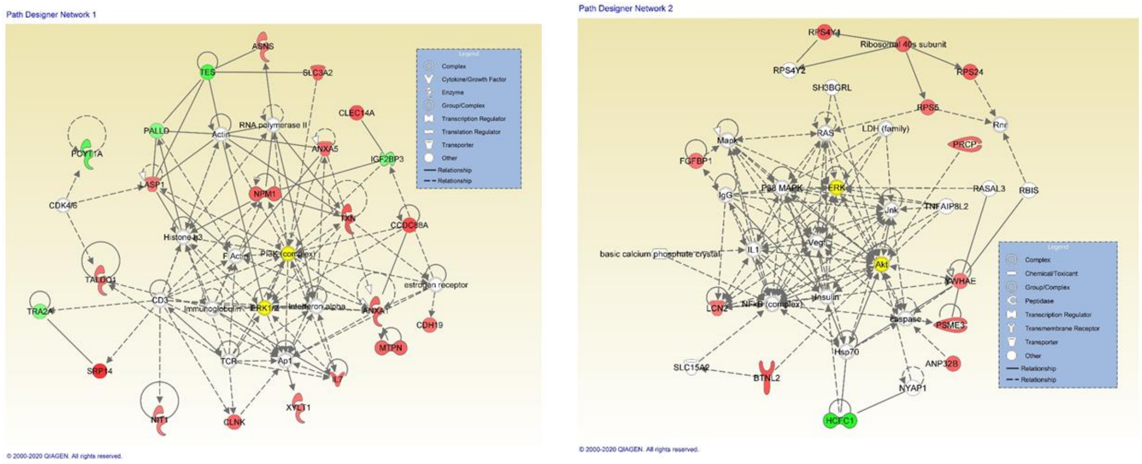

Figure S3. Sulforaphane regulated pathways in Ishikawa endometrial cancer cell. Top two networks of sulforaphane-regulated molecules identified by Ingenuity analysis implicate AKT and ERK1/2 signaling in the mechanism of action. Red indicates upregulated expression by sulforaphane, green indicates down-regulation by sulforaphane, and yellow highlights the AKT and ERK1/2 kinases.

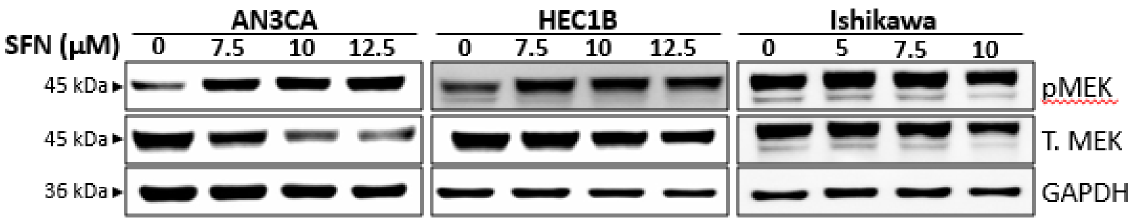

Figure S4. Sulforaphane induces phosphorylation of MEK/ERK pathway. Endometrial cancer cells (AN3CA, Hec1B and Ishikawa) were treated with sulforaphane at indicated concentrations for 24 h and cell lysate were analyzed by western blot with antibodies against phosphorylated MEK, Total MEK for MEK/ERK signaling. GAPDH was used as a reference gene. Representative blots are shown. SFN, sulforaphane.
