# Supplementary material for: Preclinical Efficacy and Involvement of AKT, mTOR, and ERK Kinases in the Mechanism of Sulforaphane against Endometrial Cancer
Source: Cancers (Basel). 2020 May 18;12(5):1273. doi: 10.3390/cancers12051273 (PMC7281543; doi:10.3390/cancers12051273)
Supplement: Supplementary file 1 [file cancers-12-01273-s001.zip › cancers-800856 supplementary/Figure S5. The whole western blot figures.pptx]

## Slide 1
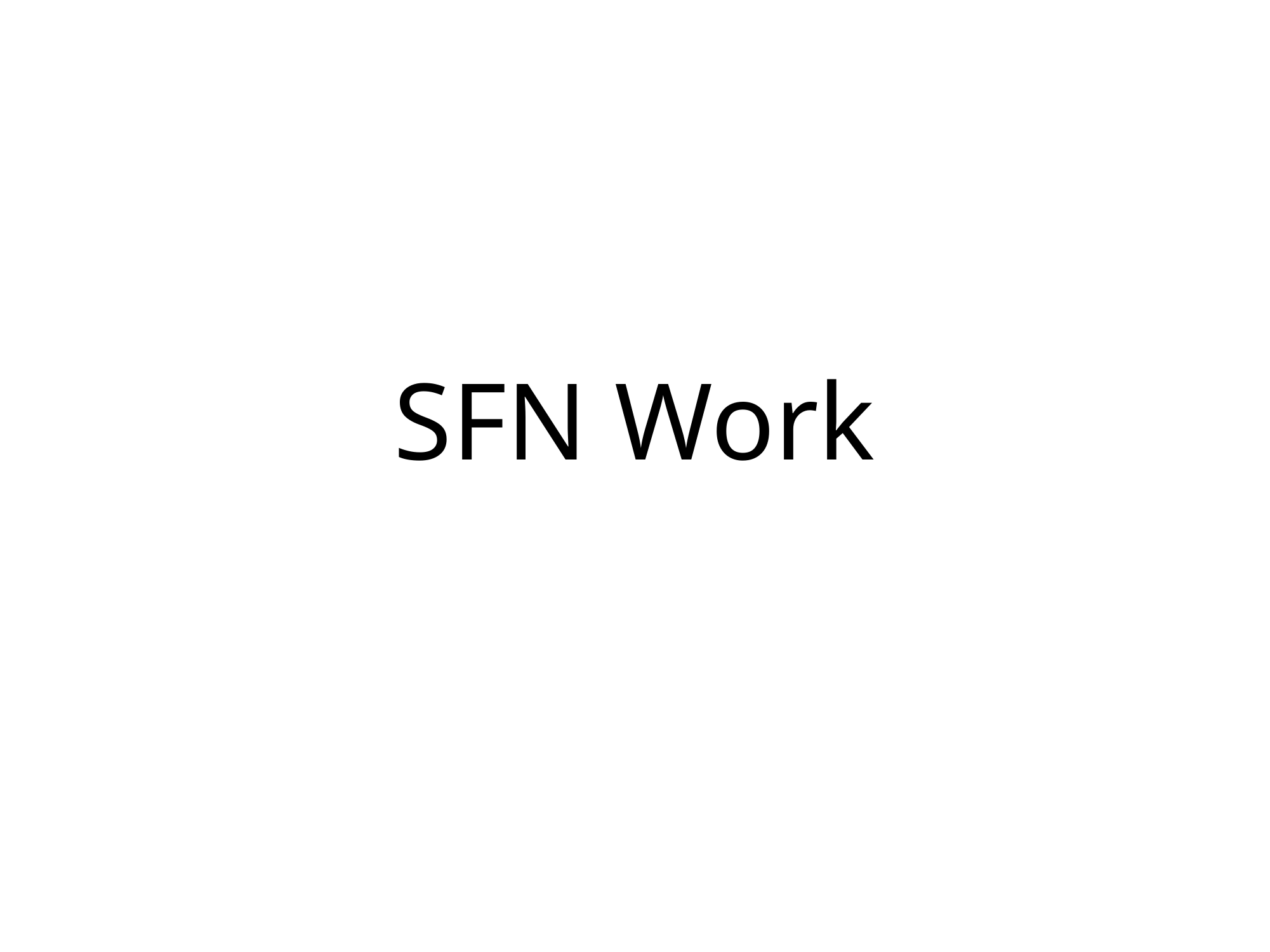

# SFN Work

## Slide 2
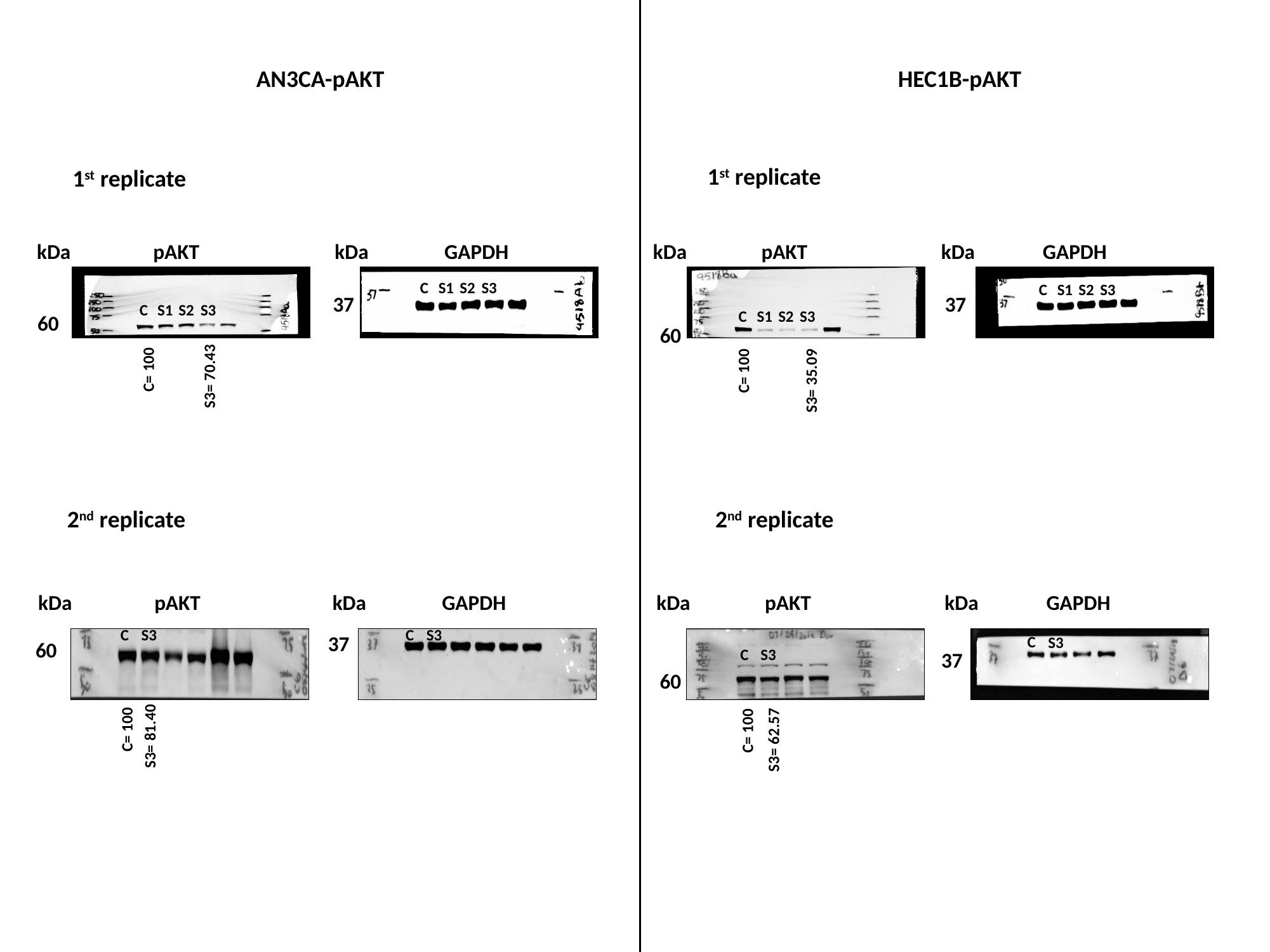

AN3CA-pAKT
HEC1B-pAKT
1st replicate
1st replicate
kDa
pAKT
kDa
GAPDH
kDa
pAKT
kDa
GAPDH
S2
S1
C
S3
S2
S1
C
S3
37
37
S3
C
S1
S2
S2
S1
C
S3
60
60
C= 100
C= 100
S3= 70.43
S3= 35.09
2nd replicate
2nd replicate
kDa
pAKT
kDa
GAPDH
kDa
pAKT
kDa
GAPDH
C
S3
C
S3
37
C
S3
60
C
S3
37
60
C= 100
S3= 81.40
C= 100
S3= 62.57

## Slide 3
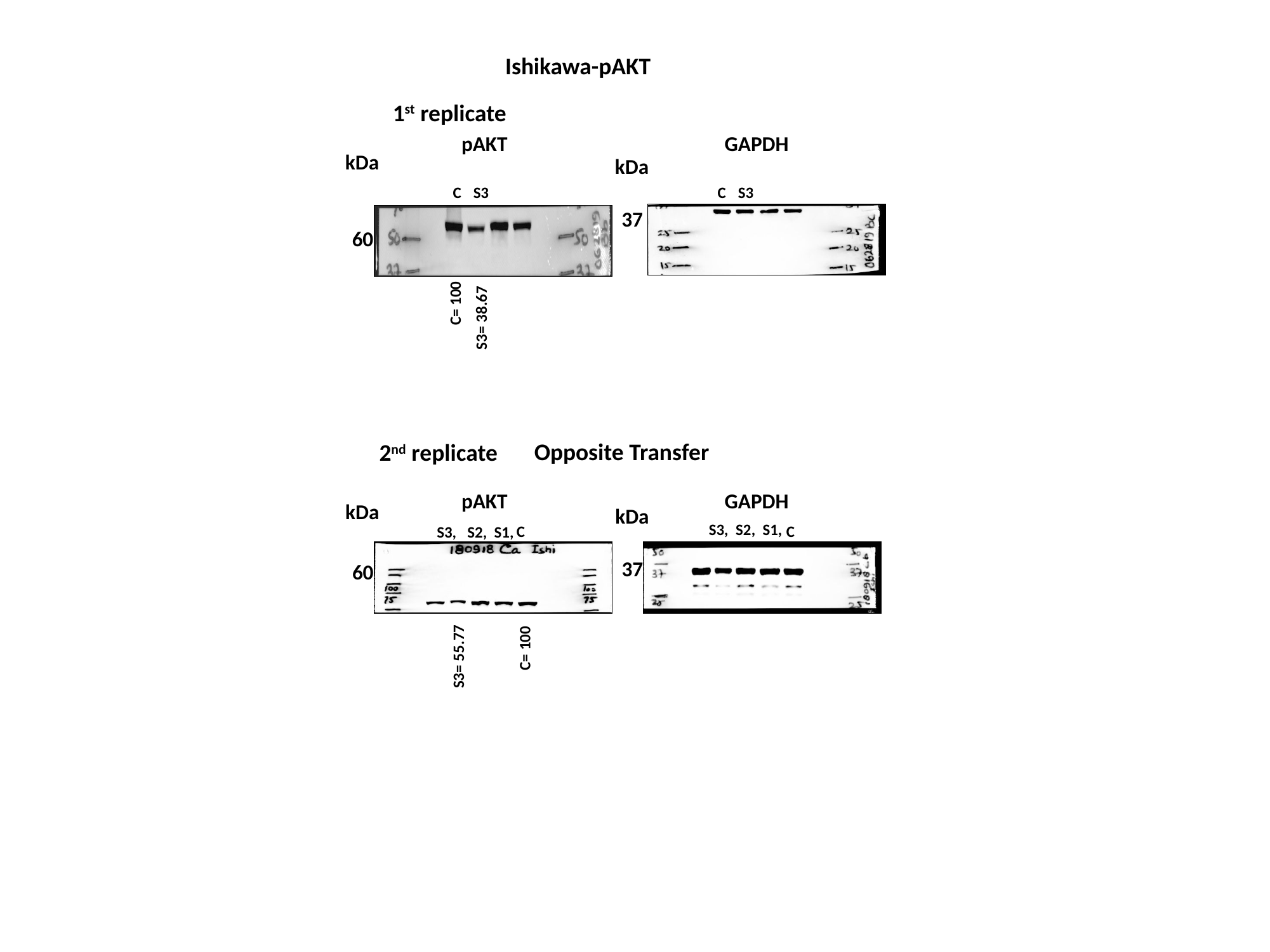

Ishikawa-pAKT
1st replicate
pAKT
GAPDH
kDa
kDa
C
S3
C
S3
37
60
C= 100
S3= 38.67
Opposite Transfer
2nd replicate
pAKT
GAPDH
kDa
kDa
 S3, S2, S1,
C
C
S3, S2, S1,
37
60
C= 100
S3= 55.77

## Slide 4
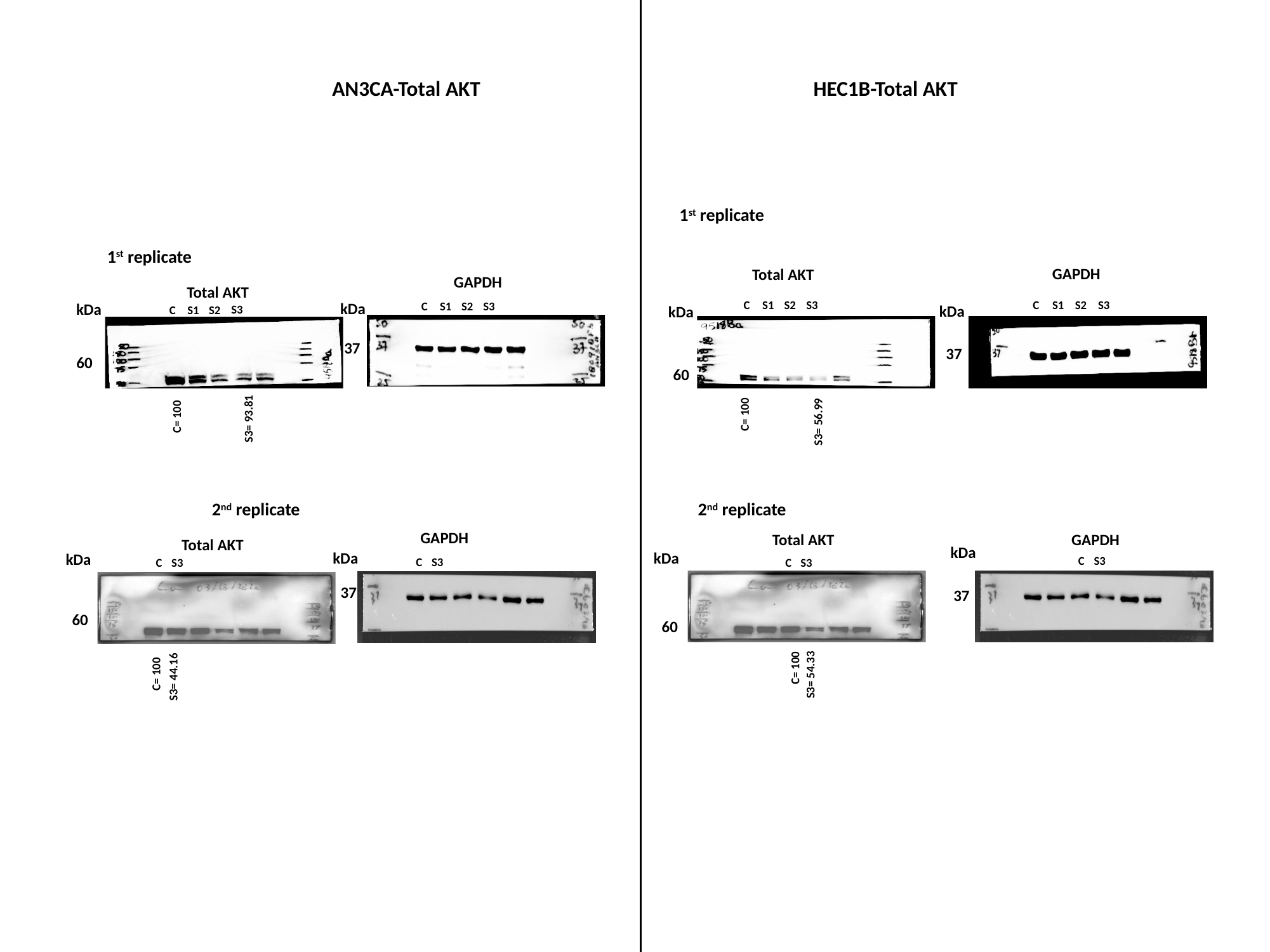

AN3CA-Total AKT
HEC1B-Total AKT
1st replicate
1st replicate
GAPDH
Total AKT
GAPDH
Total AKT
S2
S1
C
S3
S2
S1
C
S3
S2
S1
C
S3
kDa
kDa
kDa
S3
C
S1
S2
kDa
37
37
60
60
C= 100
C= 100
S3= 93.81
S3= 56.99
2nd replicate
2nd replicate
GAPDH
Total AKT
GAPDH
Total AKT
kDa
kDa
kDa
kDa
C
S3
C
S3
C
S3
C
S3
37
37
60
60
C= 100
S3= 54.33
C= 100
S3= 44.16

## Slide 5
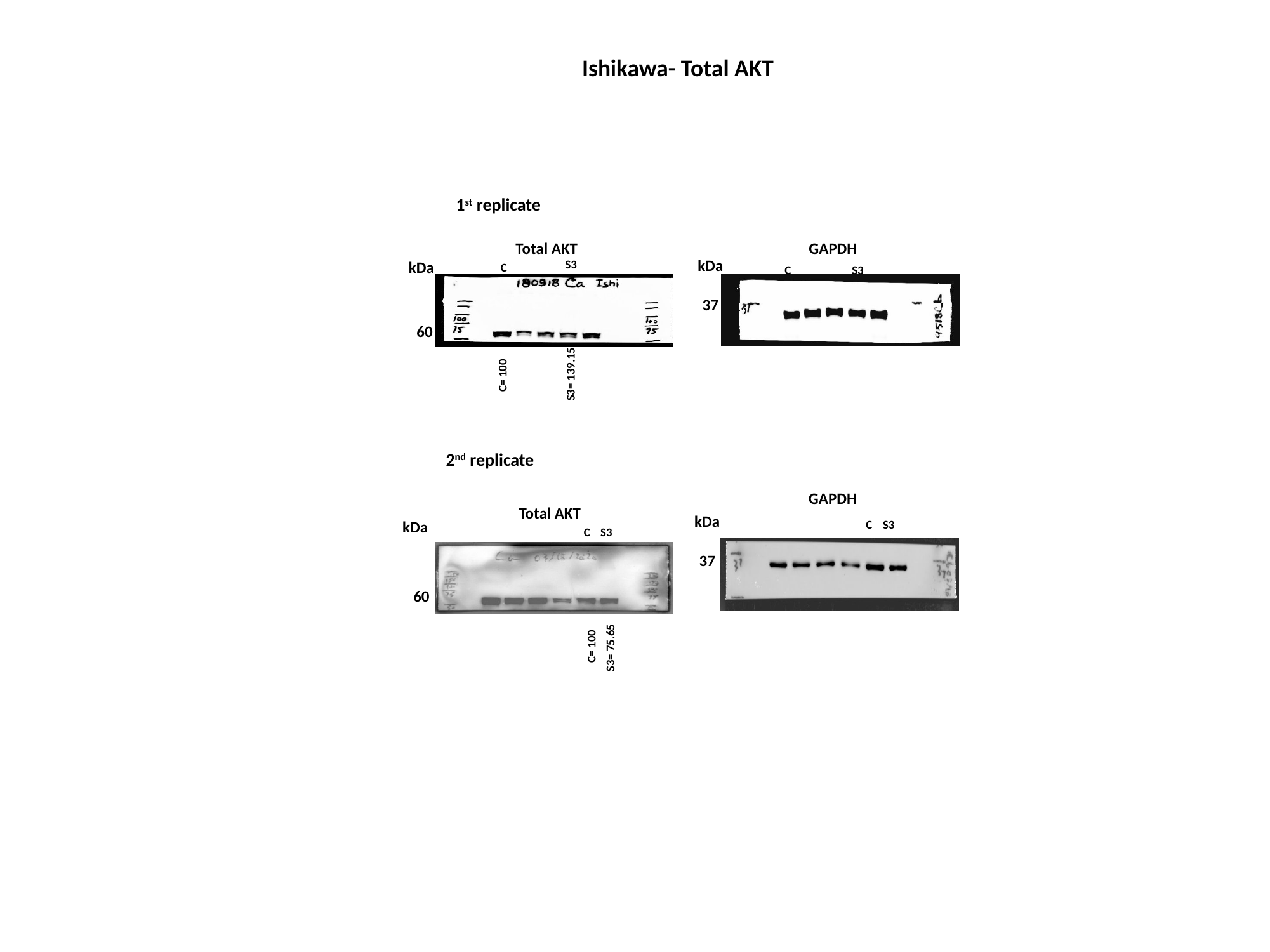

Ishikawa- Total AKT
1st replicate
Total AKT
GAPDH
kDa
S3
kDa
C
C
S3
37
60
S3= 139.15
C= 100
2nd replicate
GAPDH
Total AKT
kDa
S3
C
kDa
S3
C
37
60
C= 100
S3= 75.65

## Slide 6
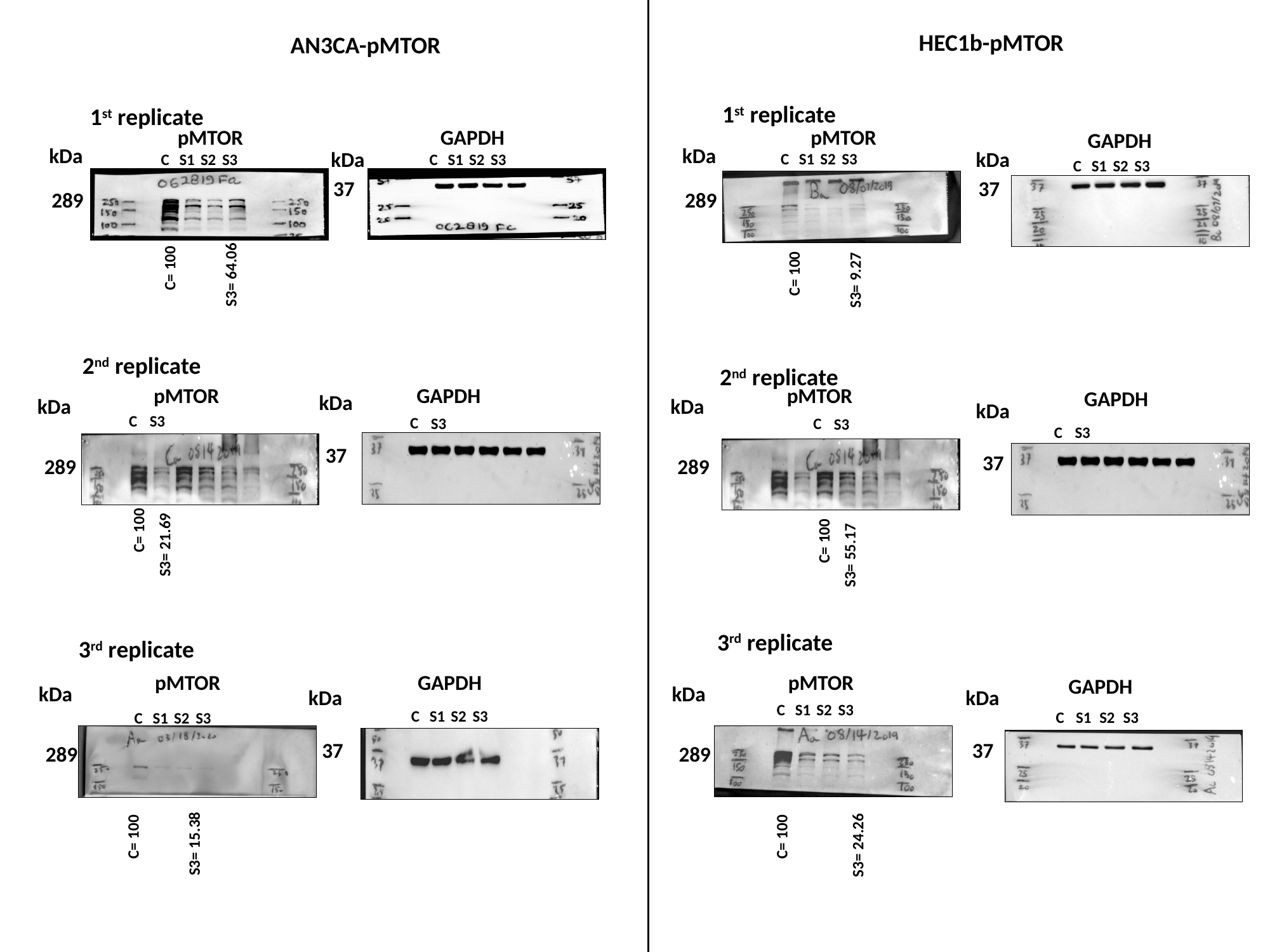

HEC1b-pMTOR
AN3CA-pMTOR
1st replicate
1st replicate
pMTOR
GAPDH
pMTOR
GAPDH
kDa
kDa
kDa
kDa
S2
S1
C
S3
S2
S1
C
S3
S2
S1
C
S3
S2
S1
C
S3
37
37
289
289
C= 100
C= 100
S3= 64.06
S3= 9.27
2nd replicate
2nd replicate
pMTOR
GAPDH
pMTOR
GAPDH
kDa
kDa
kDa
kDa
C
S3
C
S3
C
S3
C
S3
37
37
289
289
C= 100
C= 100
S3= 21.69
S3= 55.17
3rd replicate
3rd replicate
pMTOR
GAPDH
pMTOR
GAPDH
kDa
kDa
kDa
kDa
S2
S1
C
S3
S2
S1
C
S3
S2
S1
C
S3
S2
S1
C
S3
37
37
289
289
C= 100
C= 100
S3= 15.38
S3= 24.26

## Slide 7
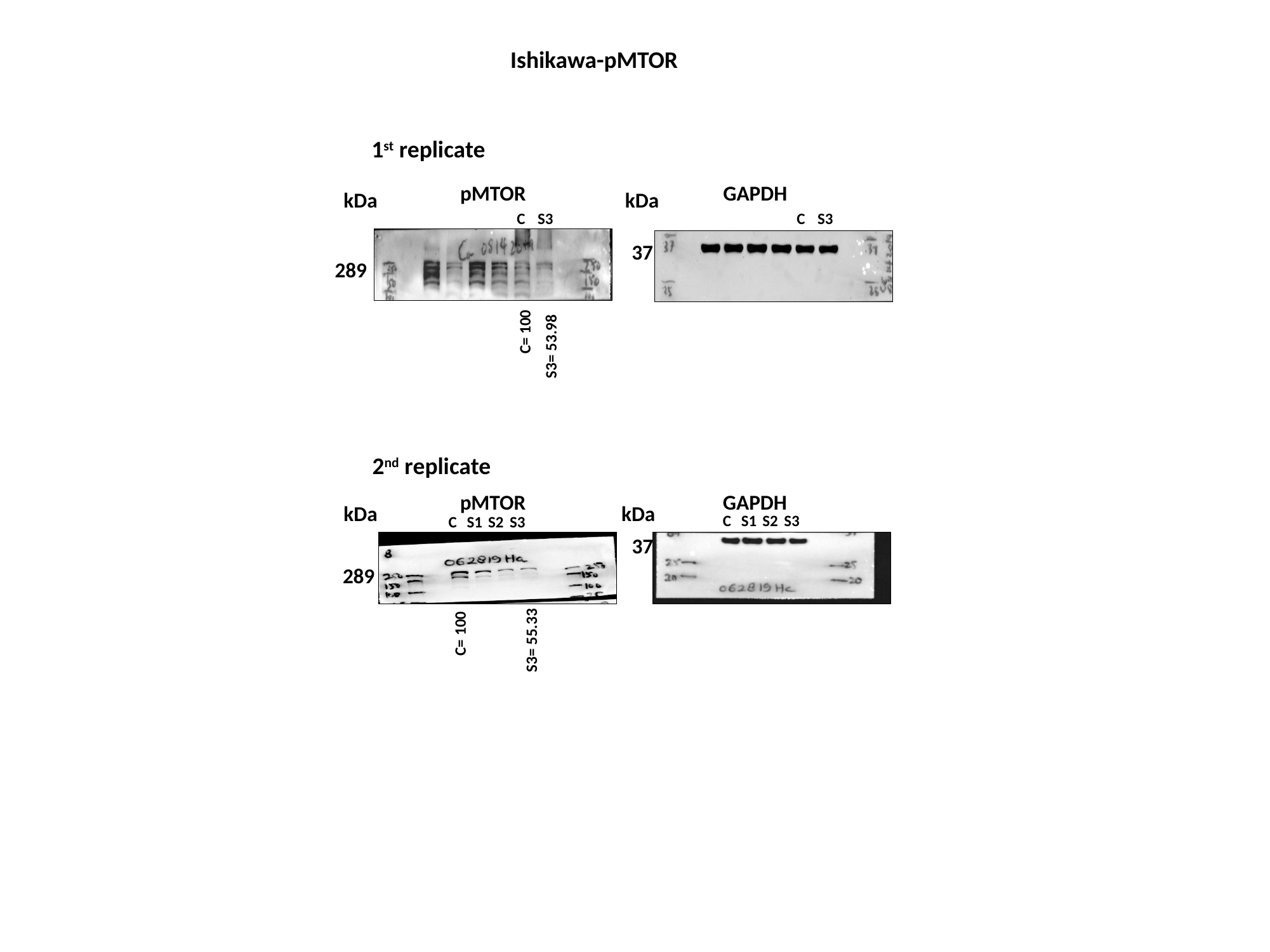

Ishikawa-pMTOR
1st replicate
pMTOR
GAPDH
kDa
kDa
C
S3
C
S3
37
289
C= 100
S3= 53.98
2nd replicate
pMTOR
GAPDH
kDa
kDa
S2
S1
C
S3
S2
S1
C
S3
37
289
C= 100
S3= 55.33

## Slide 8
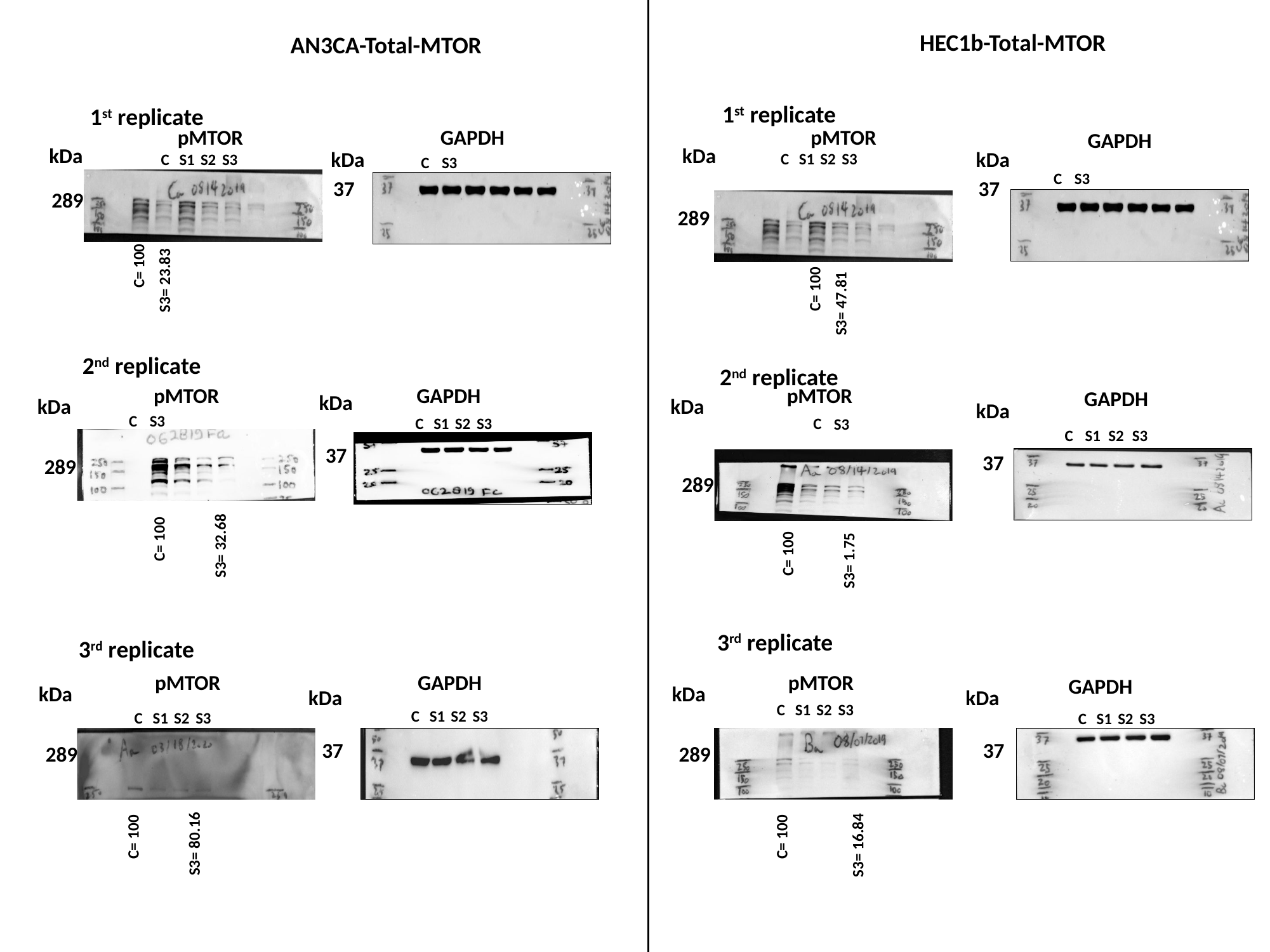

HEC1b-Total-MTOR
AN3CA-Total-MTOR
1st replicate
1st replicate
pMTOR
GAPDH
pMTOR
GAPDH
kDa
kDa
kDa
kDa
S2
S1
C
S3
S2
S1
C
S3
C
S3
C
S3
37
37
289
289
C= 100
S3= 23.83
C= 100
S3= 47.81
2nd replicate
2nd replicate
pMTOR
GAPDH
pMTOR
GAPDH
kDa
kDa
kDa
kDa
C
S3
S2
S1
C
S3
C
S3
S2
S1
C
S3
37
37
289
289
C= 100
S3= 32.68
C= 100
S3= 1.75
3rd replicate
3rd replicate
pMTOR
GAPDH
pMTOR
GAPDH
kDa
kDa
kDa
kDa
S2
S1
C
S3
S2
S1
C
S3
S2
S1
C
S3
S2
S1
C
S3
37
37
289
289
C= 100
C= 100
S3= 80.16
S3= 16.84

## Slide 9
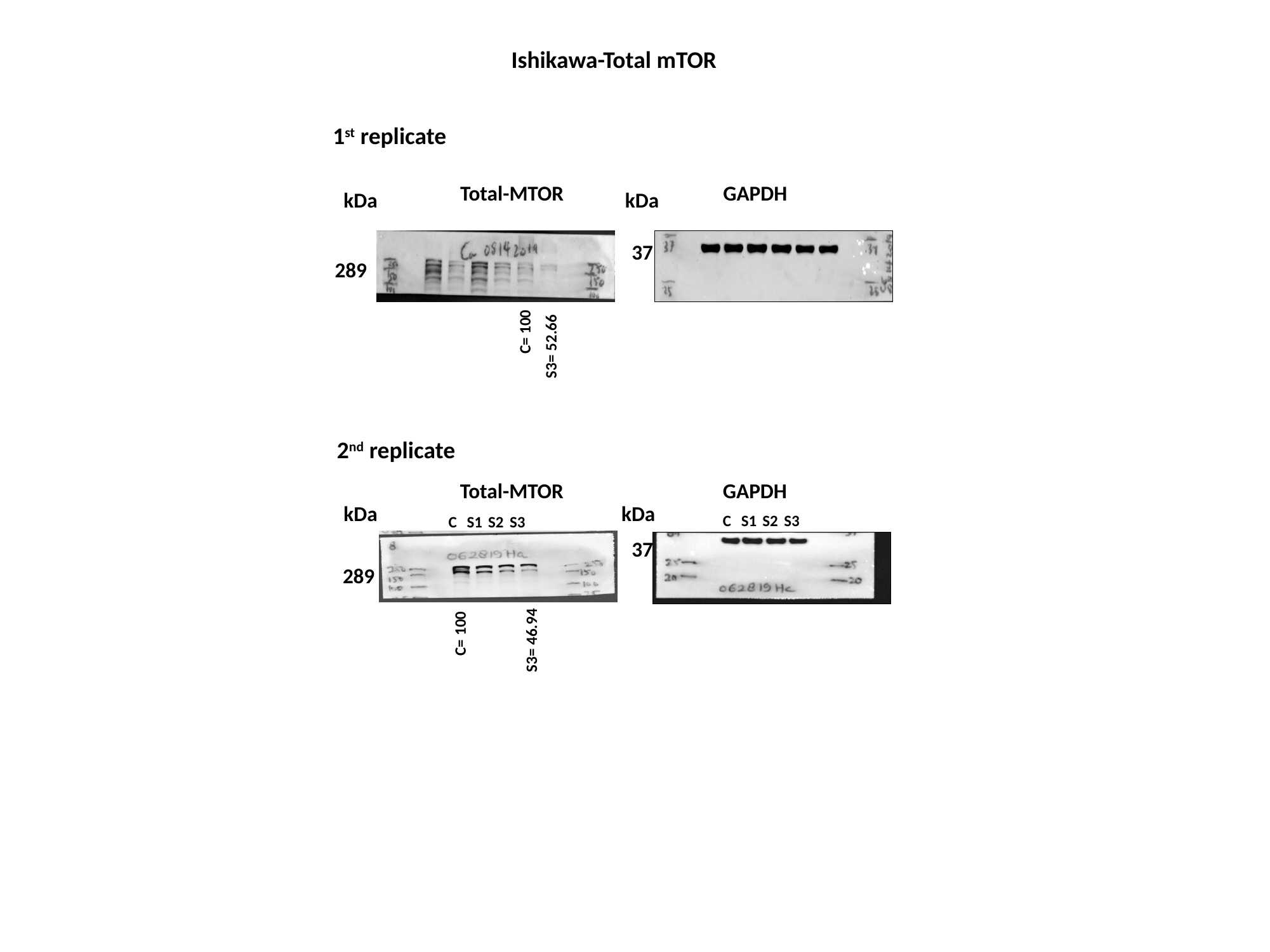

Ishikawa-Total mTOR
1st replicate
Total-MTOR
GAPDH
kDa
kDa
37
289
C= 100
S3= 52.66
2nd replicate
Total-MTOR
GAPDH
kDa
kDa
S2
S1
C
S3
S2
S1
C
S3
37
289
C= 100
S3= 46.94

## Slide 10
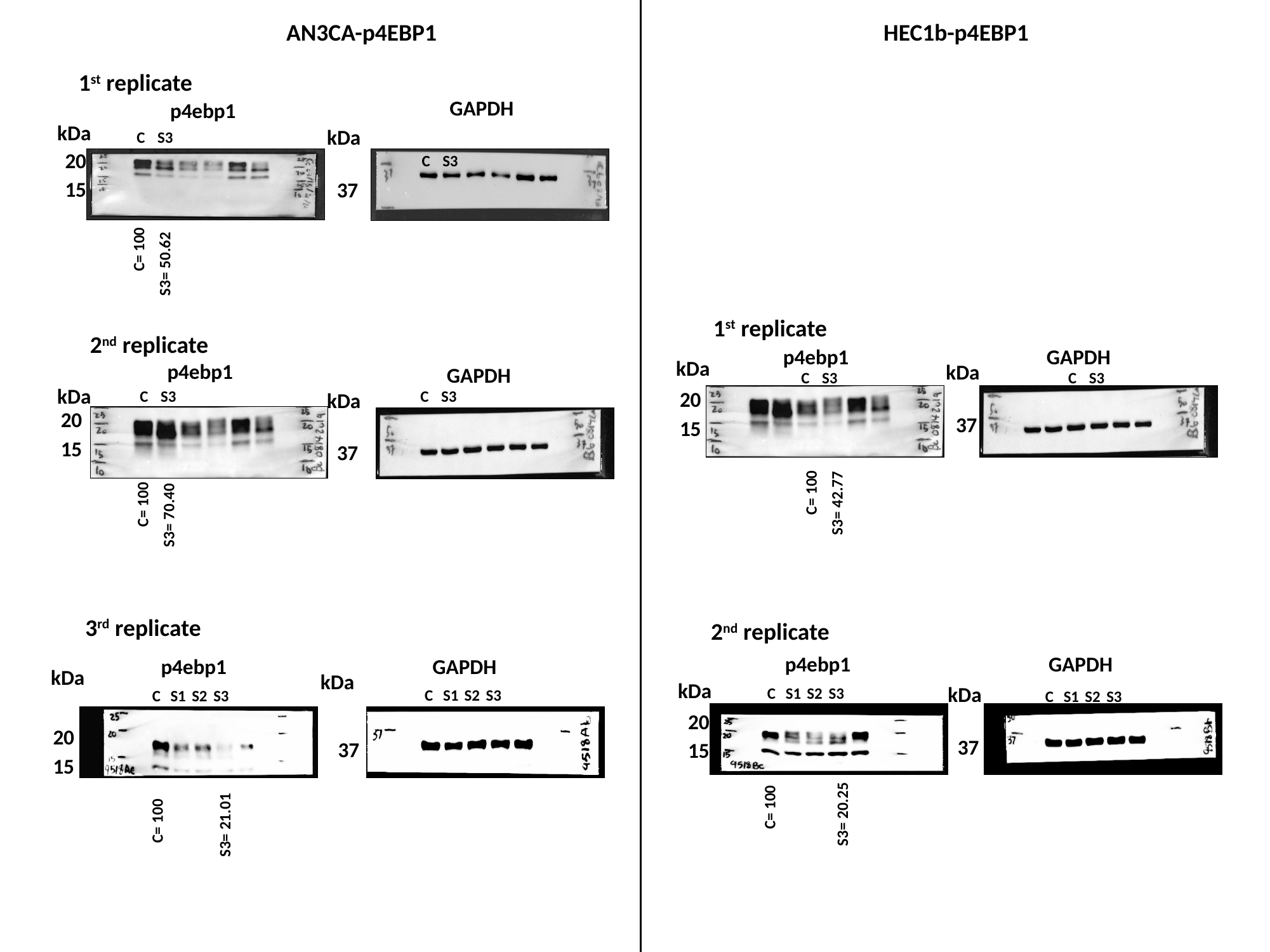

AN3CA-p4EBP1
HEC1b-p4EBP1
1st replicate
GAPDH
p4ebp1
kDa
kDa
C
S3
20
C
S3
15
37
C= 100
S3= 50.62
1st replicate
2nd replicate
p4ebp1
GAPDH
kDa
p4ebp1
kDa
GAPDH
C
S3
C
S3
kDa
C
S3
20
C
S3
kDa
20
37
15
15
37
C= 100
S3= 42.77
C= 100
S3= 70.40
3rd replicate
2nd replicate
p4ebp1
GAPDH
p4ebp1
GAPDH
kDa
kDa
kDa
kDa
S2
S1
C
S3
S2
S1
C
S3
S2
S1
C
S3
S2
S1
C
S3
20
20
37
37
15
15
C= 100
S3= 20.25
C= 100
S3= 21.01

## Slide 11
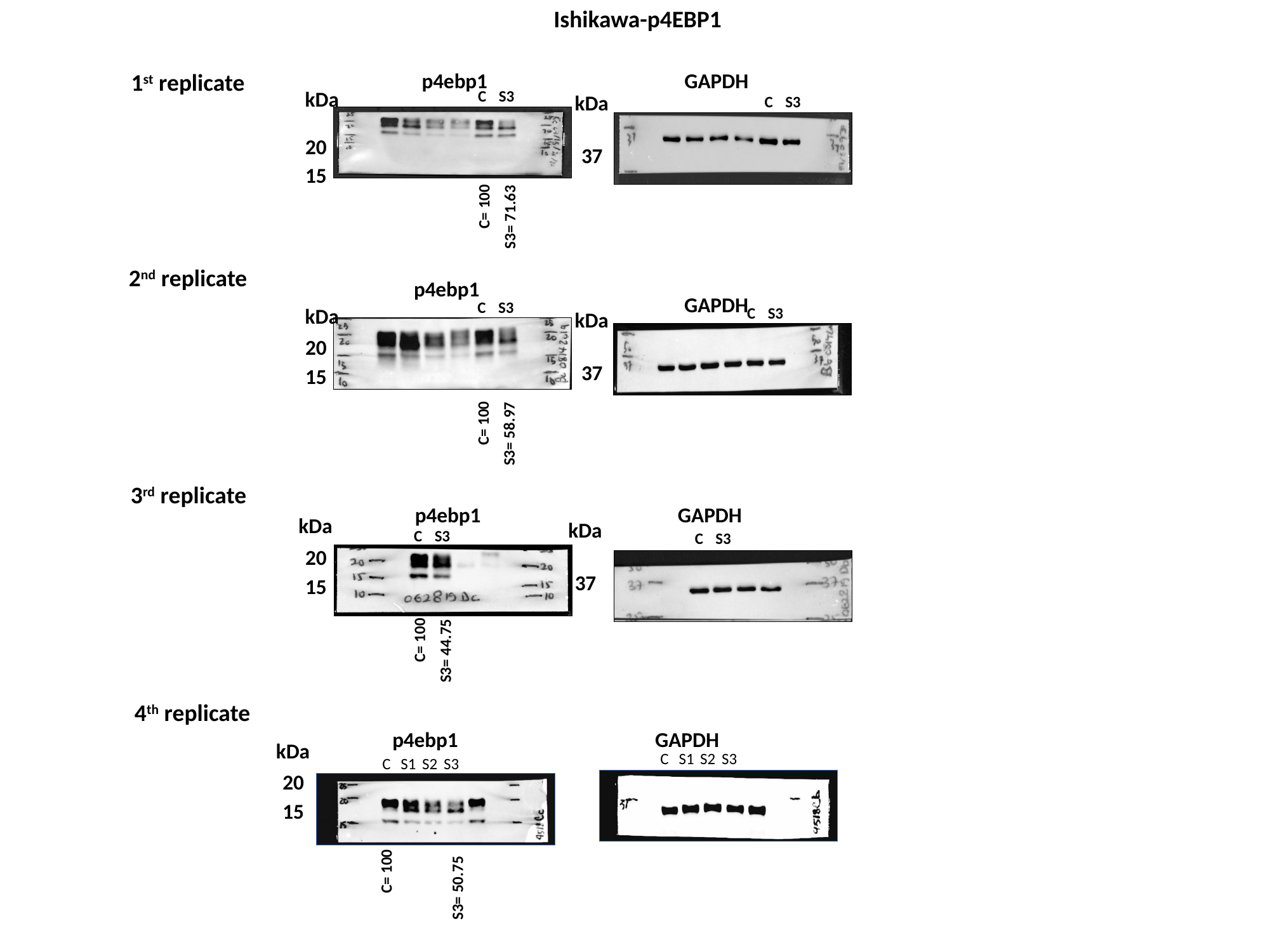

Ishikawa-p4EBP1
1st replicate
p4ebp1
GAPDH
kDa
C
S3
kDa
C
S3
20
37
15
C= 100
S3= 71.63
2nd replicate
p4ebp1
GAPDH
C
S3
kDa
C
S3
kDa
20
37
15
C= 100
S3= 58.97
3rd replicate
p4ebp1
GAPDH
kDa
kDa
C
S3
C
S3
20
37
15
C= 100
S3= 44.75
4th replicate
p4ebp1
GAPDH
kDa
S2
S1
C
S3
S2
S1
C
S3
20
15
C= 100
S3= 50.75

## Slide 12
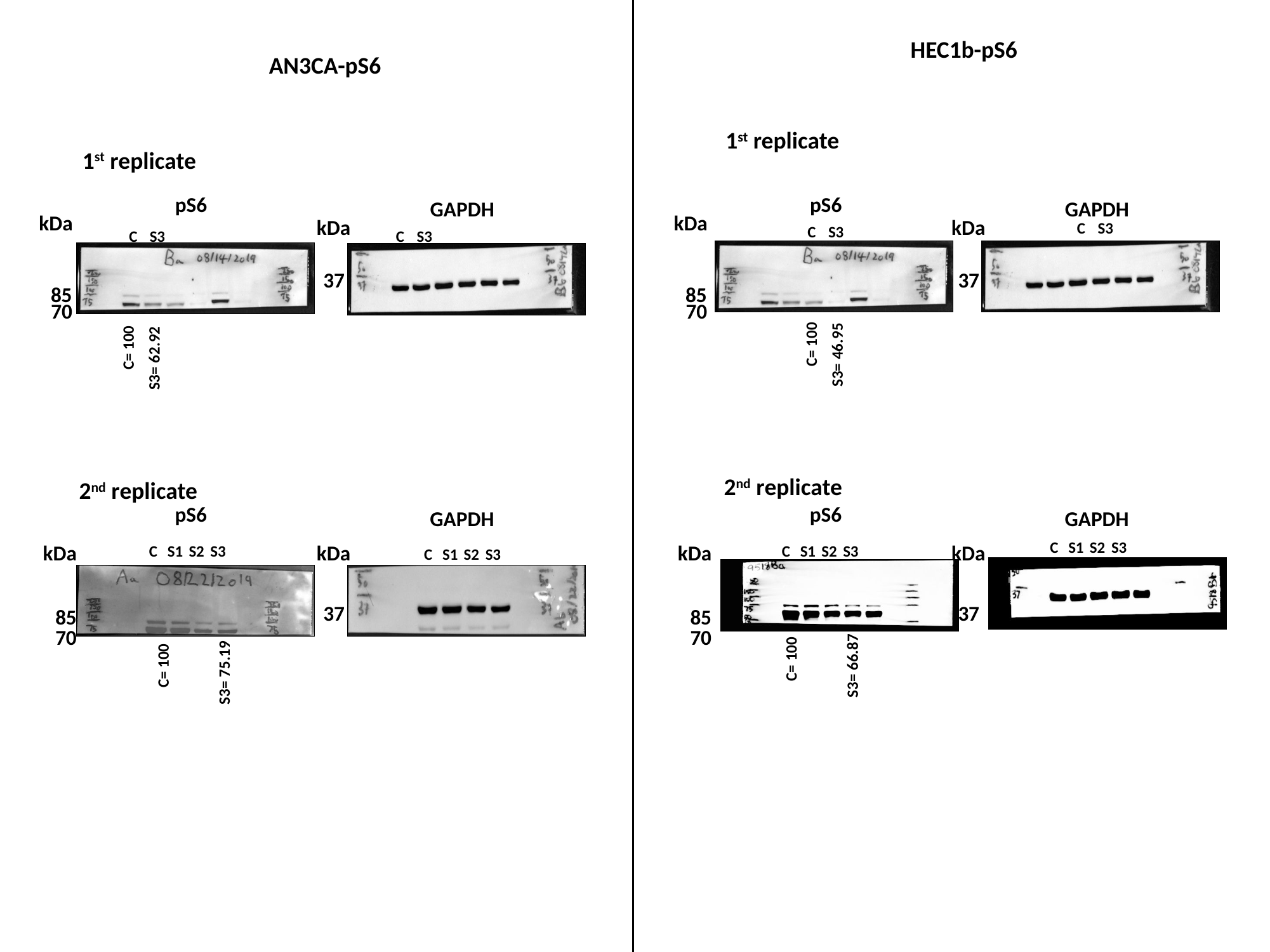

HEC1b-pS6
AN3CA-pS6
1st replicate
1st replicate
pS6
pS6
GAPDH
GAPDH
kDa
kDa
kDa
kDa
C
S3
C
S3
C
S3
C
S3
37
37
85
85
70
70
C= 100
C= 100
S3= 46.95
S3= 62.92
2nd replicate
2nd replicate
pS6
pS6
GAPDH
GAPDH
S2
S1
C
S3
kDa
kDa
kDa
kDa
S2
S1
C
S3
S2
S1
C
S3
S2
S1
C
S3
37
37
85
85
70
70
C= 100
C= 100
S3= 66.87
S3= 75.19

## Slide 13
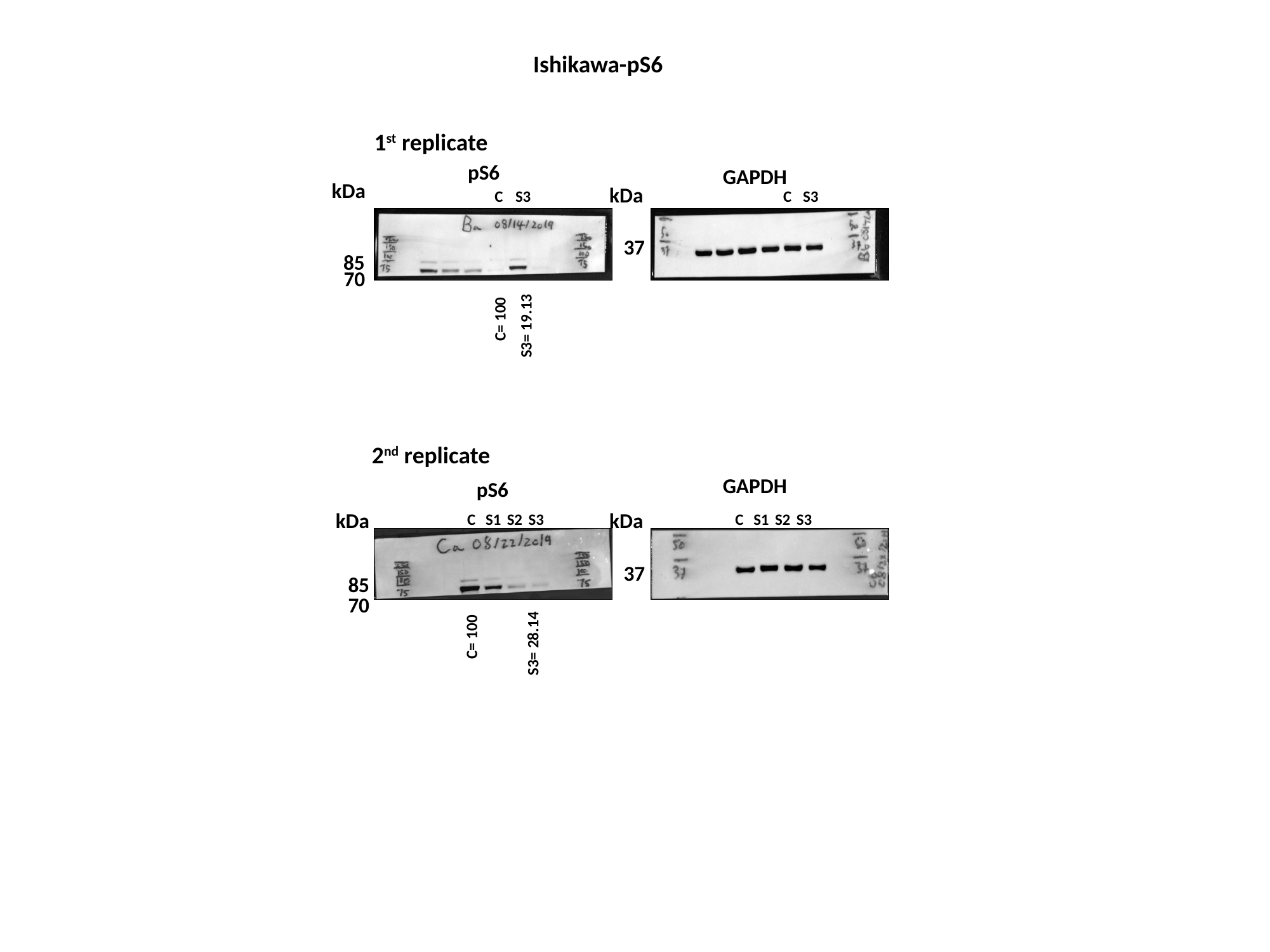

Ishikawa-pS6
1st replicate
pS6
GAPDH
kDa
kDa
C
C
S3
S3
37
85
70
C= 100
S3= 19.13
2nd replicate
GAPDH
pS6
kDa
kDa
S2
S1
C
S3
S2
S1
C
S3
37
85
70
C= 100
S3= 28.14

## Slide 14
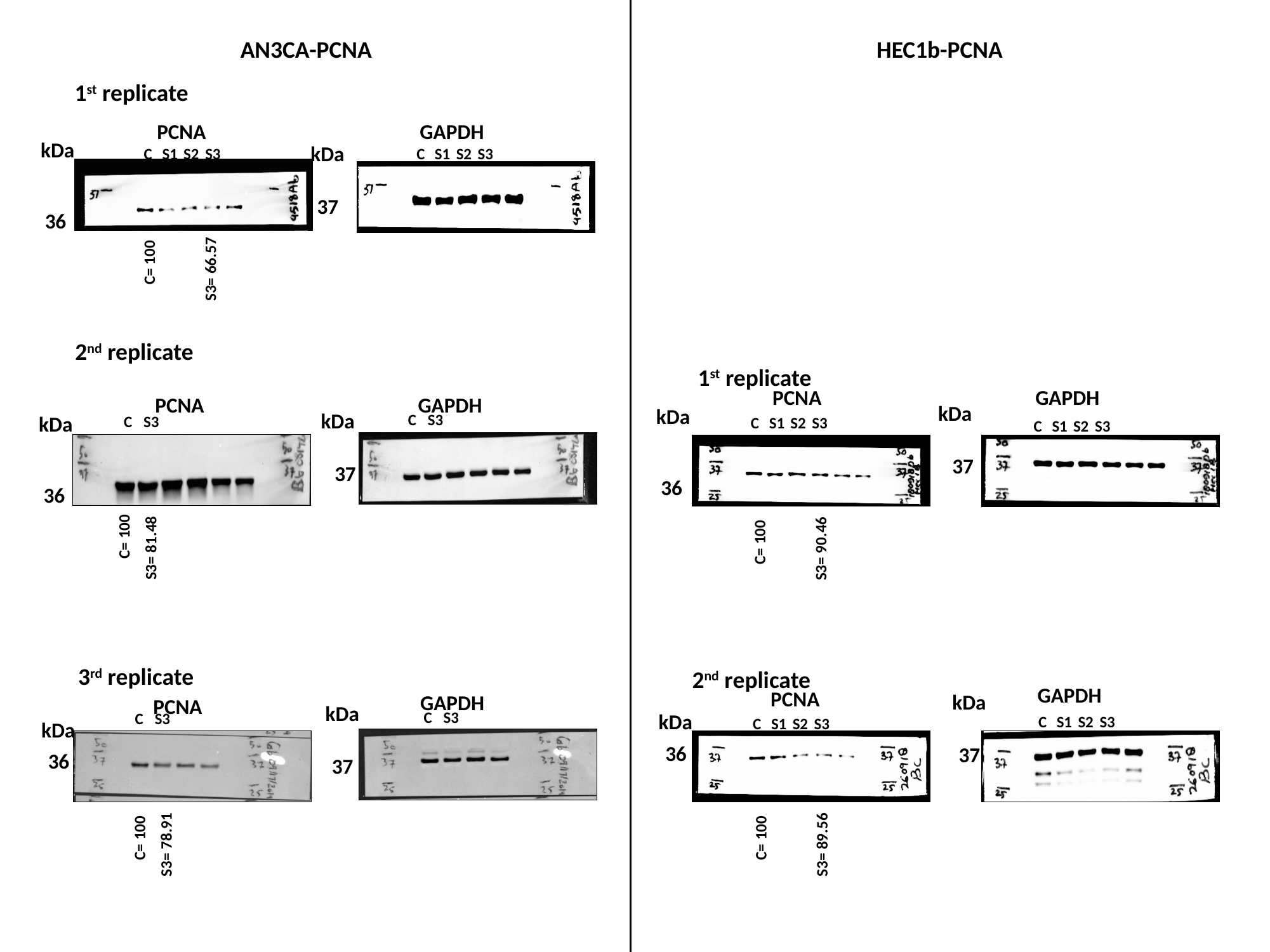

AN3CA-PCNA
HEC1b-PCNA
1st replicate
PCNA
GAPDH
kDa
kDa
S2
S1
C
S3
S2
S1
C
S3
37
36
C= 100
S3= 66.57
2nd replicate
1st replicate
PCNA
GAPDH
PCNA
GAPDH
kDa
kDa
kDa
C
S3
kDa
C
S3
S2
S1
C
S3
S2
S1
C
S3
37
37
36
36
C= 100
C= 100
S3= 81.48
S3= 90.46
3rd replicate
2nd replicate
GAPDH
PCNA
kDa
GAPDH
PCNA
kDa
C
S3
kDa
C
S3
S2
S1
C
S3
S2
S1
C
S3
kDa
36
37
36
37
C= 100
C= 100
S3= 78.91
S3= 89.56

## Slide 15
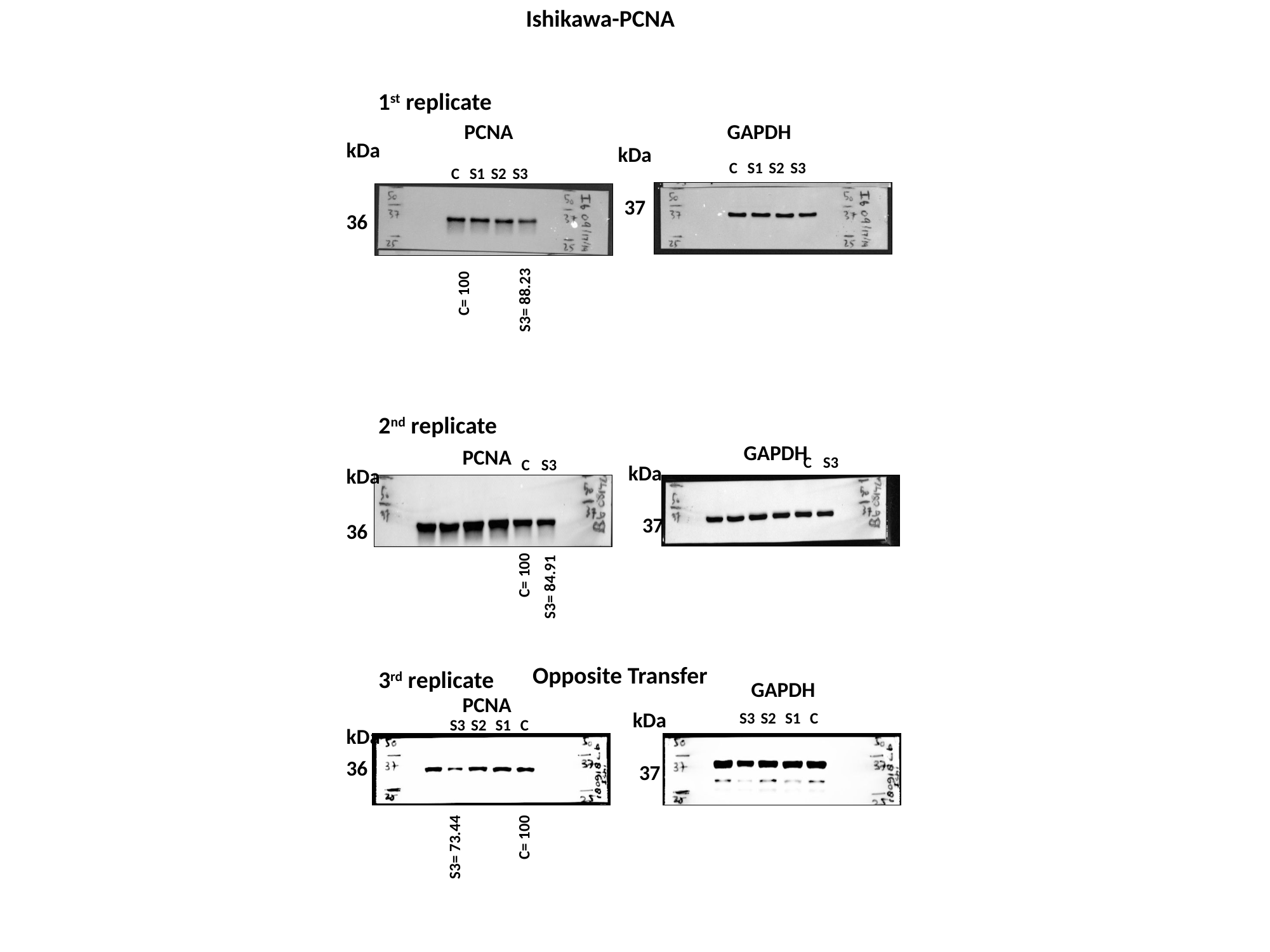

Ishikawa-PCNA
1st replicate
PCNA
GAPDH
kDa
kDa
S2
S1
C
S3
S2
S1
C
S3
37
36
C= 100
S3= 88.23
2nd replicate
GAPDH
PCNA
C
S3
C
S3
kDa
kDa
37
36
C= 100
S3= 84.91
Opposite Transfer
3rd replicate
GAPDH
PCNA
kDa
S1
S2
S3
C
S1
S2
S3
C
kDa
36
37
C= 100
S3= 73.44

## Slide 16
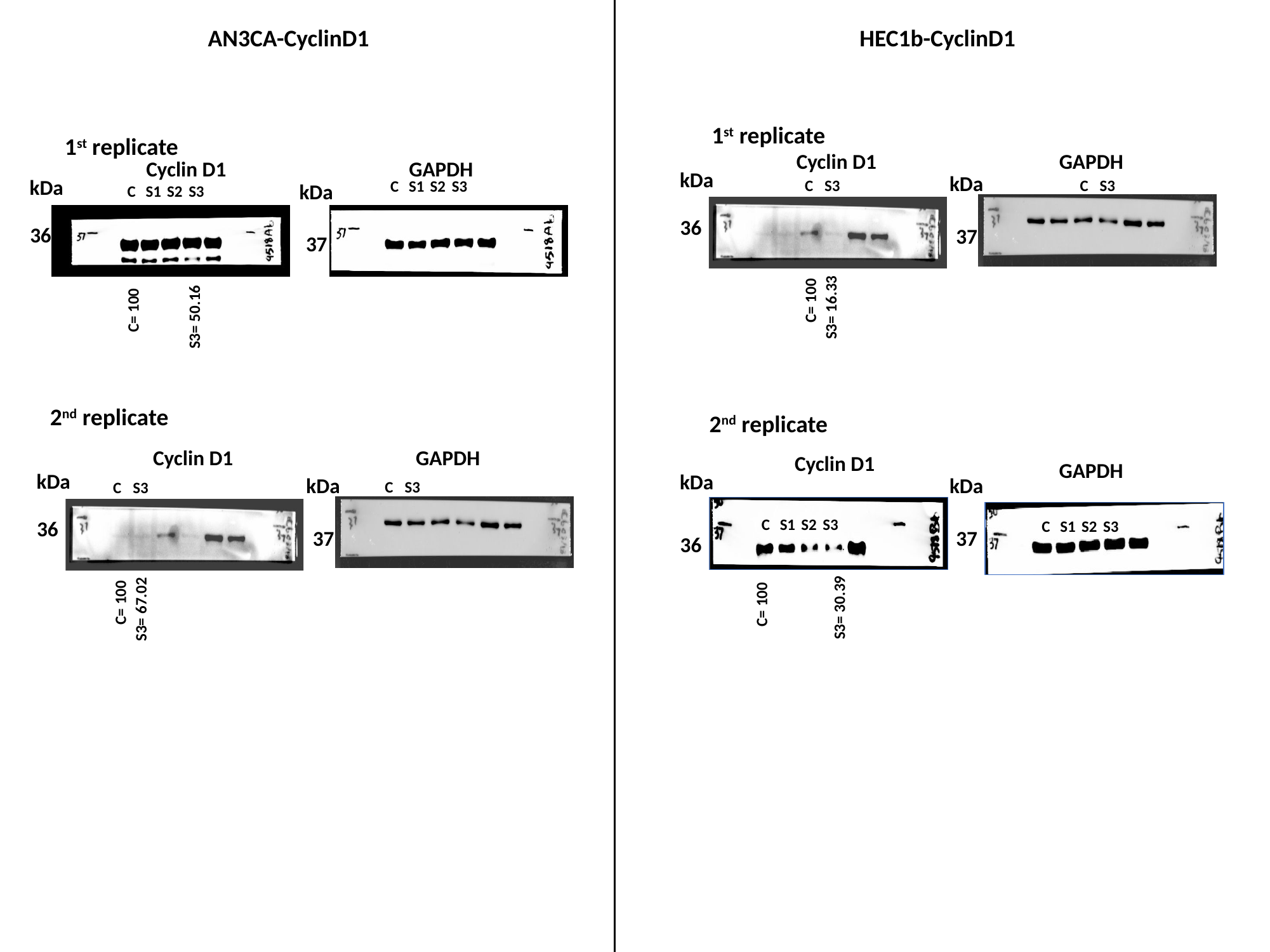

AN3CA-CyclinD1
HEC1b-CyclinD1
1st replicate
1st replicate
Cyclin D1
GAPDH
Cyclin D1
GAPDH
kDa
kDa
kDa
C
S3
C
S3
S2
S1
C
S3
kDa
S2
S1
C
S3
36
36
37
37
C= 100
S3= 16.33
C= 100
S3= 50.16
2nd replicate
2nd replicate
Cyclin D1
GAPDH
Cyclin D1
GAPDH
kDa
kDa
kDa
kDa
C
S3
C
S3
S2
S1
C
S3
36
S2
S1
C
S3
37
37
36
C= 100
C= 100
S3= 30.39
S3= 67.02

## Slide 17
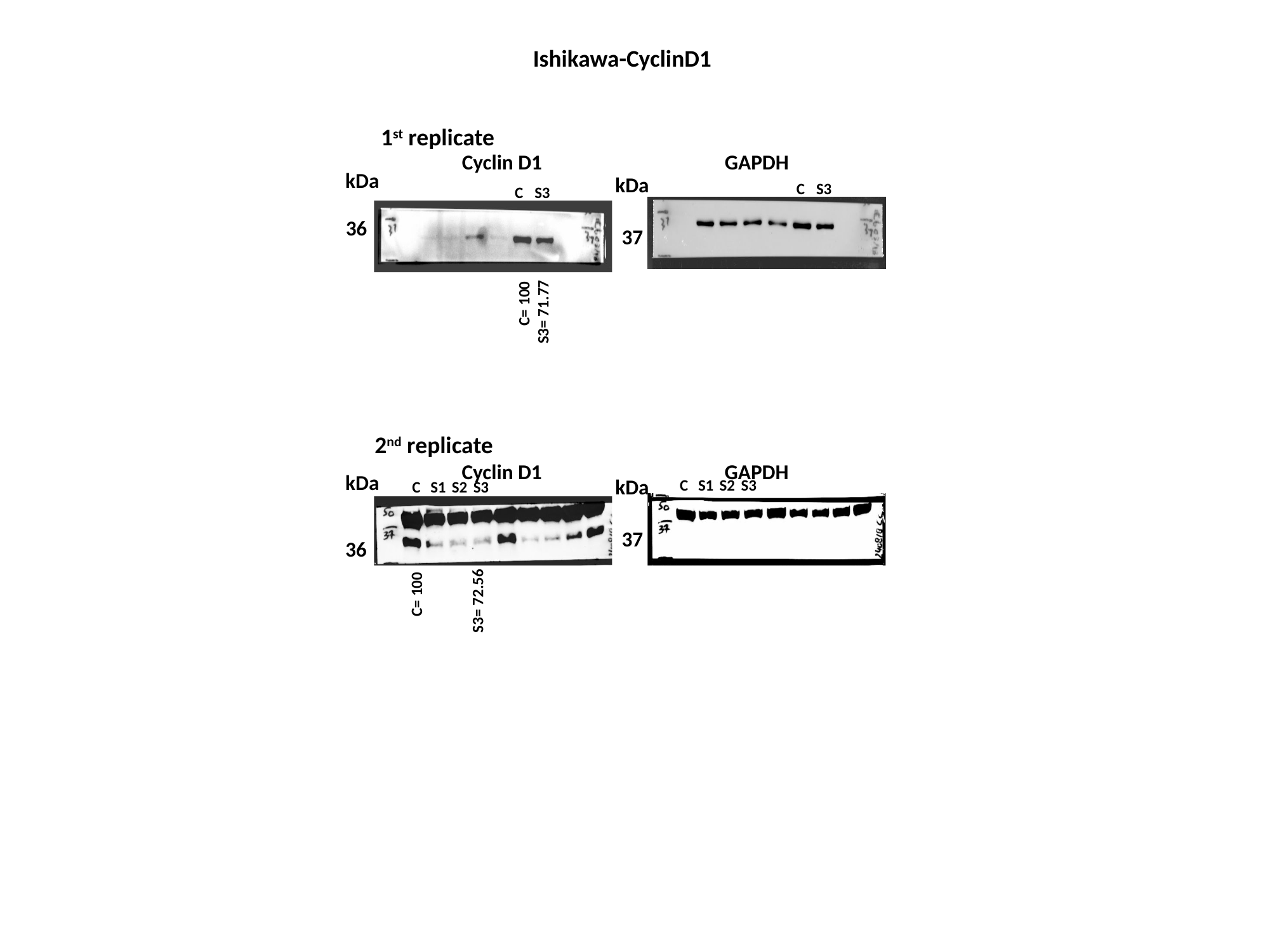

Ishikawa-CyclinD1
1st replicate
Cyclin D1
GAPDH
kDa
kDa
C
S3
C
S3
36
37
C= 100
S3= 71.77
2nd replicate
Cyclin D1
GAPDH
kDa
kDa
S2
S1
C
S3
S2
S1
C
S3
37
36
C= 100
S3= 72.56

## Slide 18
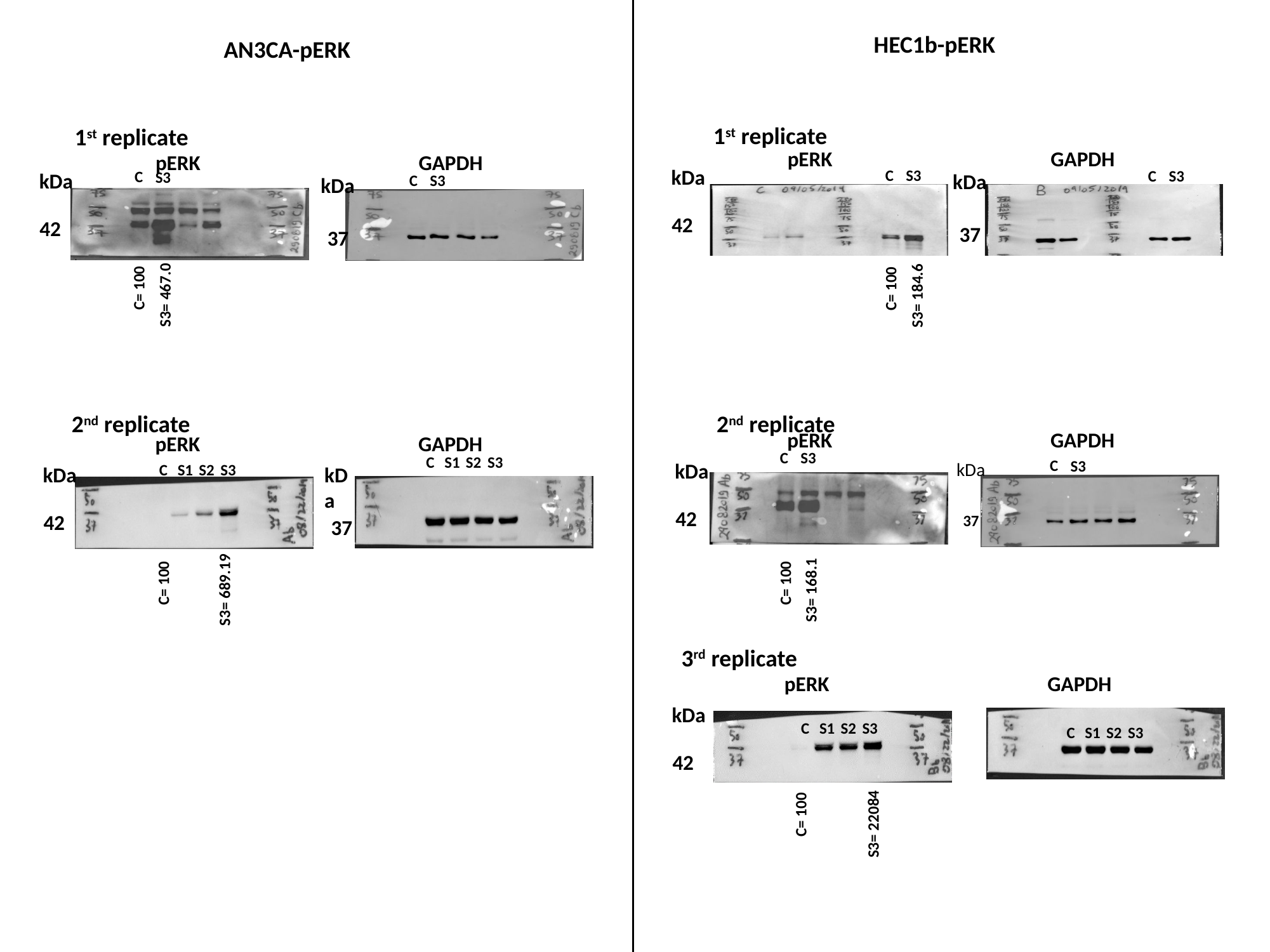

HEC1b-pERK
AN3CA-pERK
1st replicate
1st replicate
pERK
GAPDH
pERK
GAPDH
kDa
C
S3
C
S3
C
S3
kDa
kDa
C
S3
kDa
42
42
37
37
C= 100
C= 100
S3= 467.0
S3= 184.6
2nd replicate
2nd replicate
pERK
GAPDH
pERK
GAPDH
C
S3
S2
S1
C
S3
C
S3
kDa
kDa
S2
S1
C
S3
kDa
kDa
42
42
37
37
C= 100
C= 100
S3= 689.19
S3= 168.1
3rd replicate
pERK
GAPDH
kDa
S2
S1
C
S3
S2
S1
C
S3
42
C= 100
S3= 22084

## Slide 19
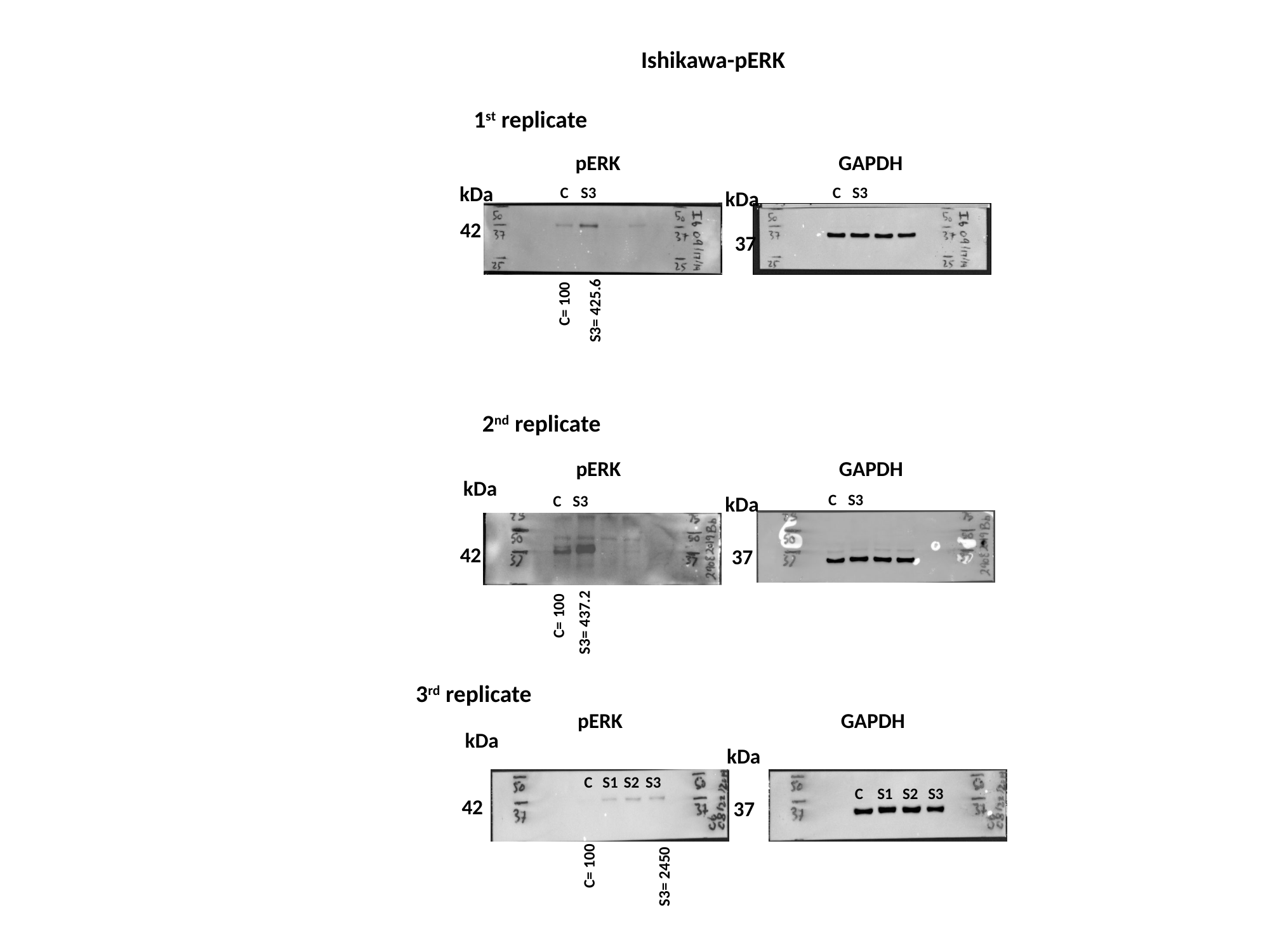

Ishikawa-pERK
1st replicate
pERK
GAPDH
kDa
C
C
S3
S3
kDa
42
37
C= 100
S3= 425.6
2nd replicate
pERK
GAPDH
kDa
C
S3
C
S3
kDa
42
37
C= 100
S3= 437.2
3rd replicate
pERK
GAPDH
kDa
kDa
S2
S1
C
S3
S2
S1
C
S3
42
37
C= 100
S3= 2450

## Slide 20
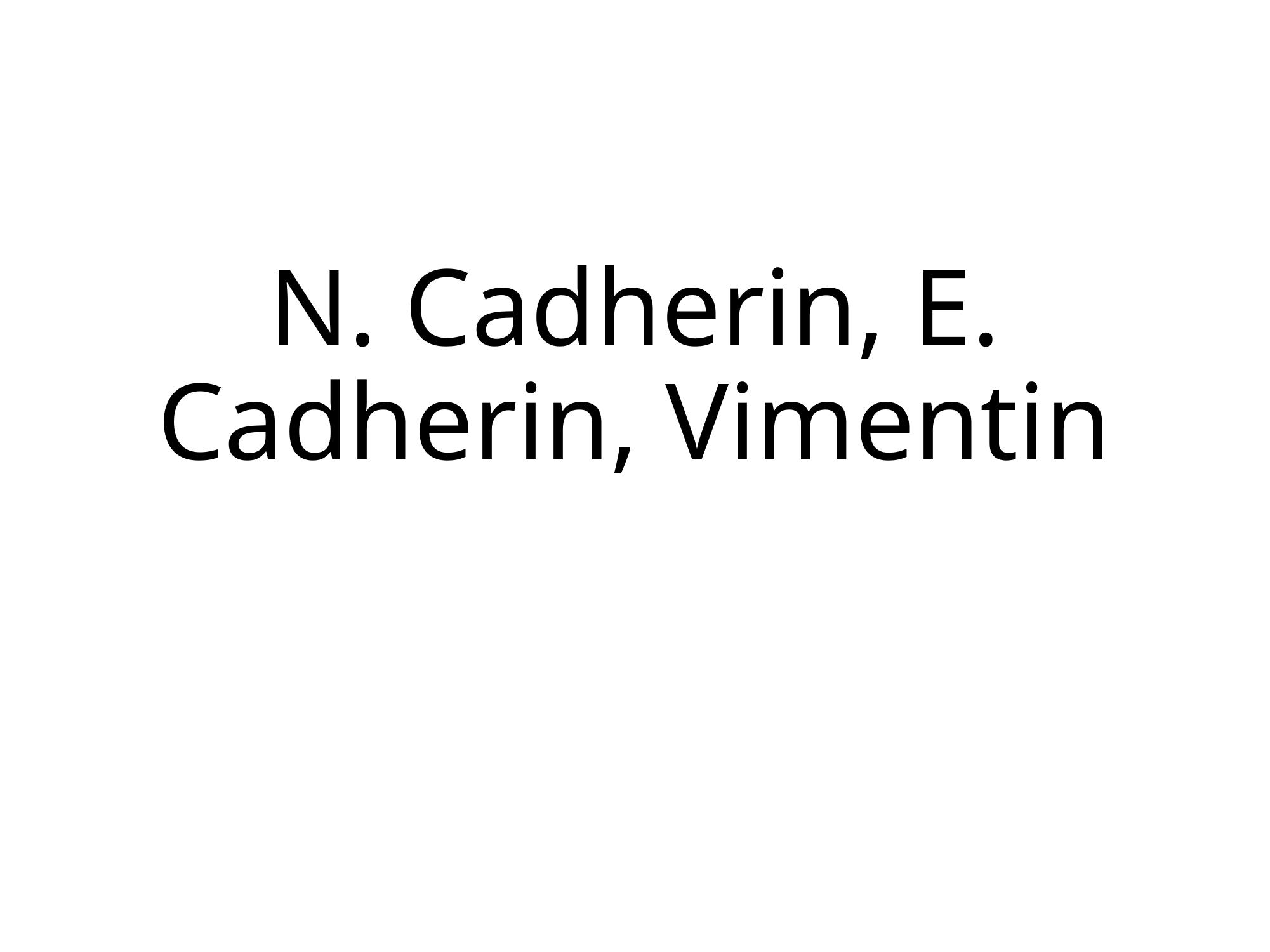

# N. Cadherin, E. Cadherin, Vimentin

## Slide 21
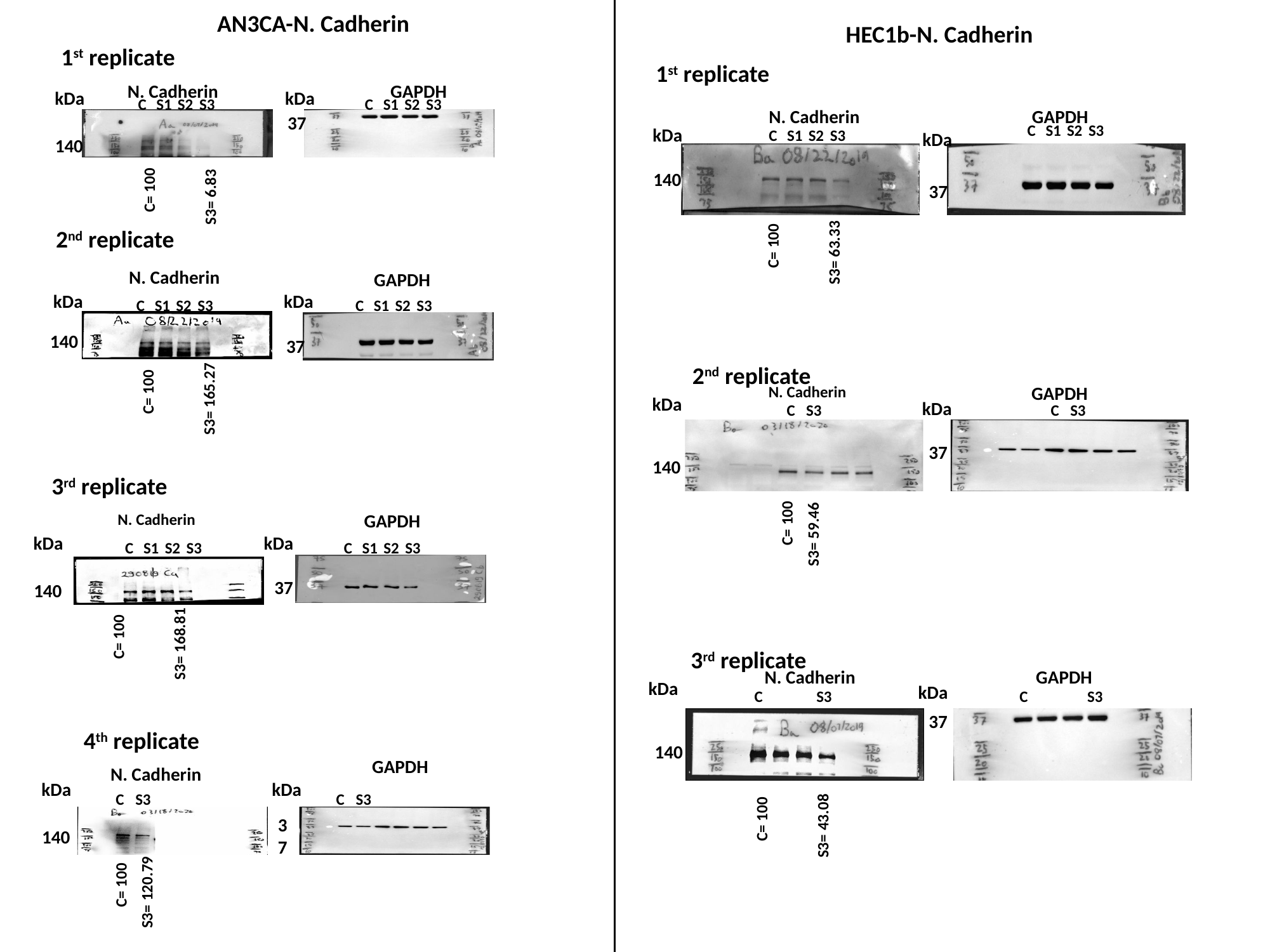

AN3CA-N. Cadherin
HEC1b-N. Cadherin
1st replicate
1st replicate
N. Cadherin
GAPDH
kDa
kDa
S2
S1
C
S3
S2
S1
C
S3
N. Cadherin
GAPDH
37
S2
S1
C
S3
kDa
S2
S1
C
S3
kDa
140
140
C= 100
37
S3= 6.83
2nd replicate
C= 100
S3= 63.33
N. Cadherin
GAPDH
kDa
kDa
S2
S1
C
S3
S2
S1
C
S3
140
37
2nd replicate
C= 100
N. Cadherin
GAPDH
S3= 165.27
kDa
kDa
C
S3
C
S3
37
140
3rd replicate
N. Cadherin
GAPDH
C= 100
S3= 59.46
kDa
kDa
S2
S1
C
S3
S2
S1
C
S3
37
140
C= 100
S3= 168.81
3rd replicate
GAPDH
N. Cadherin
kDa
kDa
C
S3
C
S3
37
4th replicate
140
GAPDH
N. Cadherin
kDa
kDa
C
S3
C
S3
C= 100
37
S3= 43.08
140
C= 100
S3= 120.79

## Slide 22
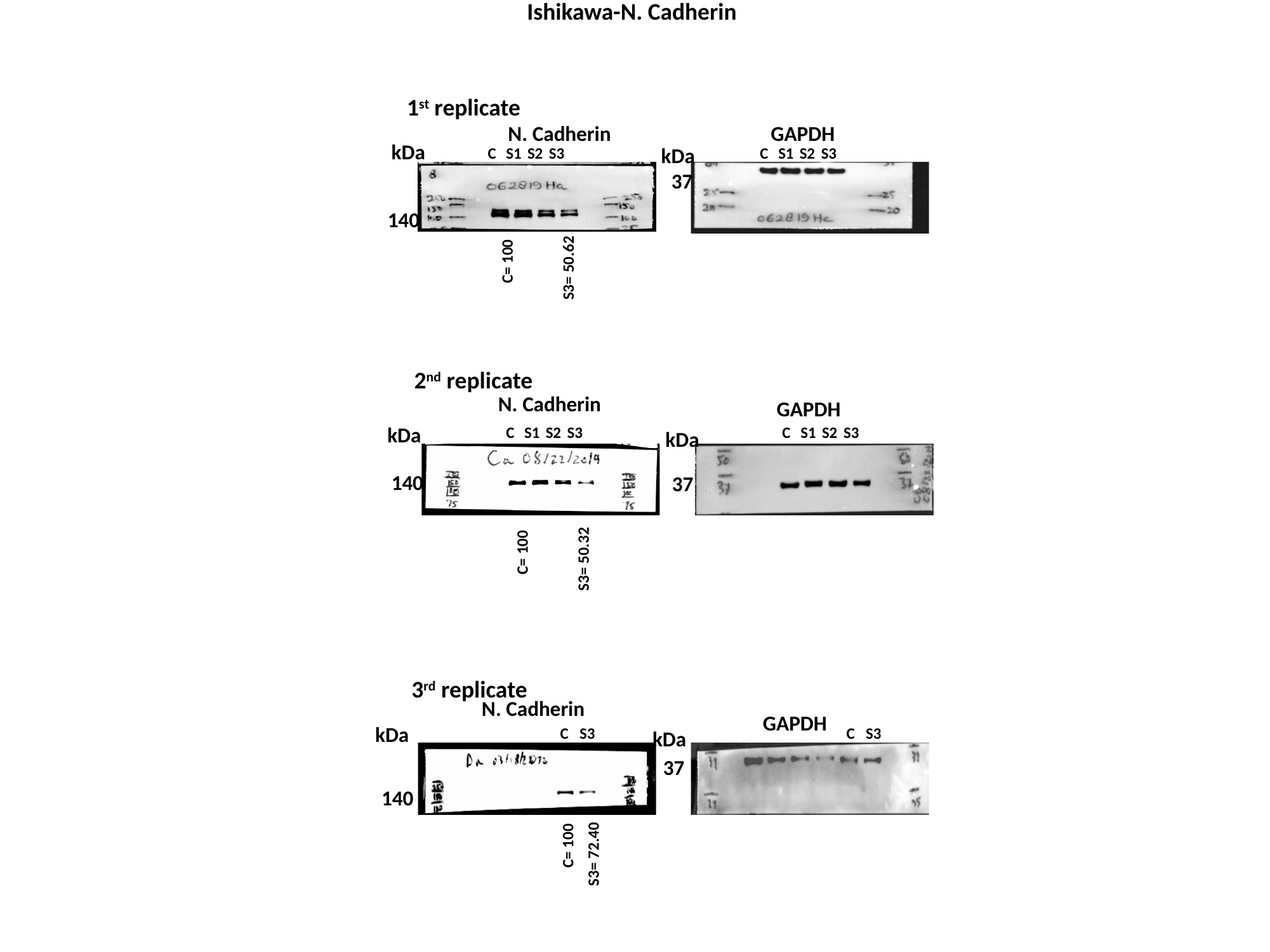

Ishikawa-N. Cadherin
1st replicate
N. Cadherin
GAPDH
kDa
kDa
S2
S1
C
S3
S2
S1
C
S3
37
140
C= 100
S3= 50.62
2nd replicate
N. Cadherin
GAPDH
kDa
S2
S1
C
S3
S2
S1
C
S3
kDa
140
37
C= 100
S3= 50.32
3rd replicate
N. Cadherin
GAPDH
kDa
C
S3
C
S3
kDa
37
140
C= 100
S3= 72.40

## Slide 23
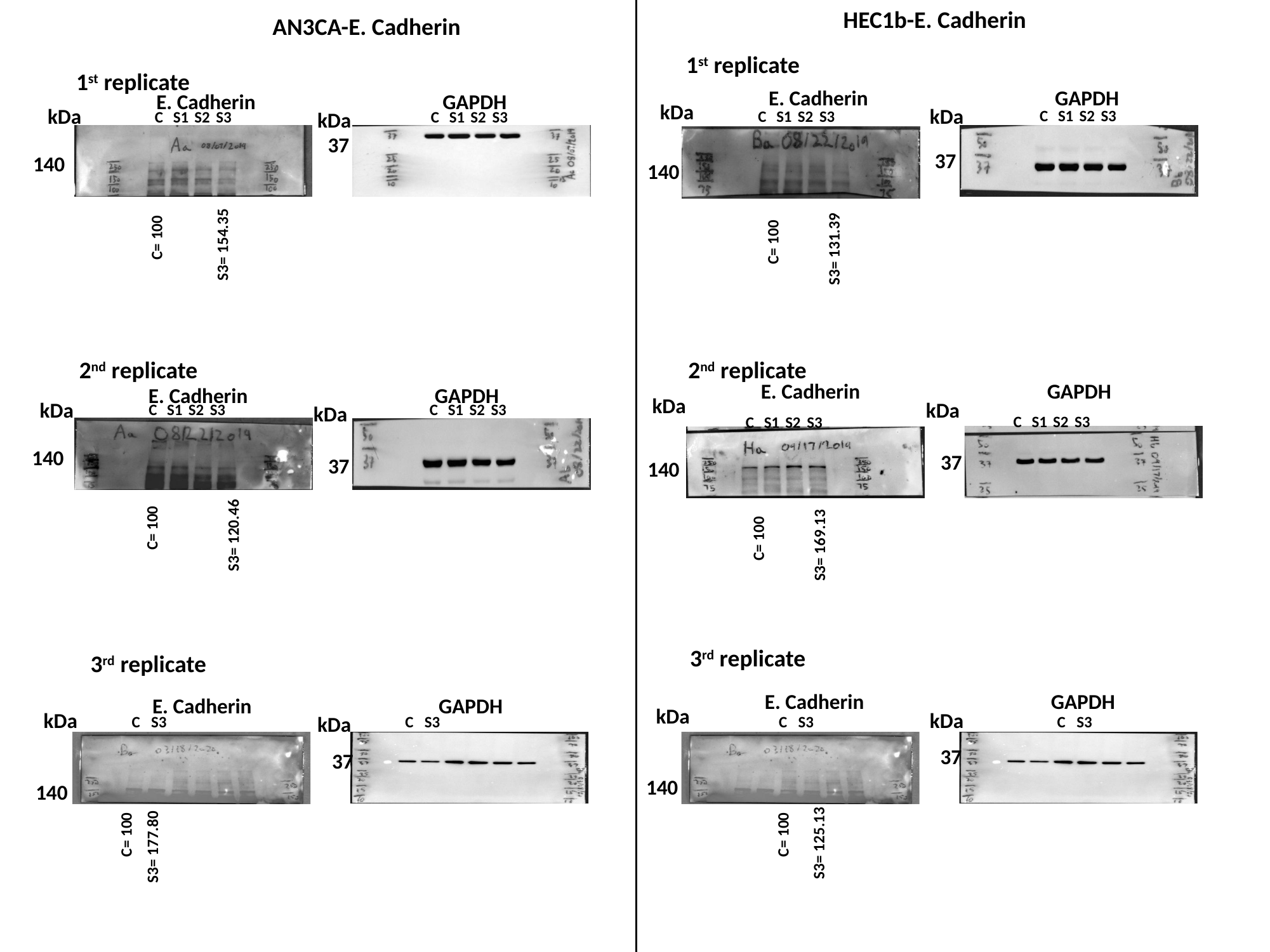

HEC1b-E. Cadherin
AN3CA-E. Cadherin
1st replicate
1st replicate
E. Cadherin
GAPDH
E. Cadherin
GAPDH
kDa
kDa
kDa
S2
S1
C
S3
S2
S1
C
S3
kDa
S2
S1
C
S3
S2
S1
C
S3
37
37
140
140
C= 100
C= 100
S3= 154.35
S3= 131.39
2nd replicate
2nd replicate
E. Cadherin
GAPDH
E. Cadherin
GAPDH
kDa
kDa
kDa
S2
S1
C
S3
S2
S1
C
S3
kDa
S2
S1
C
S3
S2
S1
C
S3
140
37
37
140
C= 100
S3= 120.46
C= 100
S3= 169.13
3rd replicate
3rd replicate
E. Cadherin
GAPDH
E. Cadherin
GAPDH
kDa
kDa
kDa
kDa
C
S3
C
S3
C
S3
C
S3
37
37
140
140
C= 100
C= 100
S3= 125.13
S3= 177.80

## Slide 24
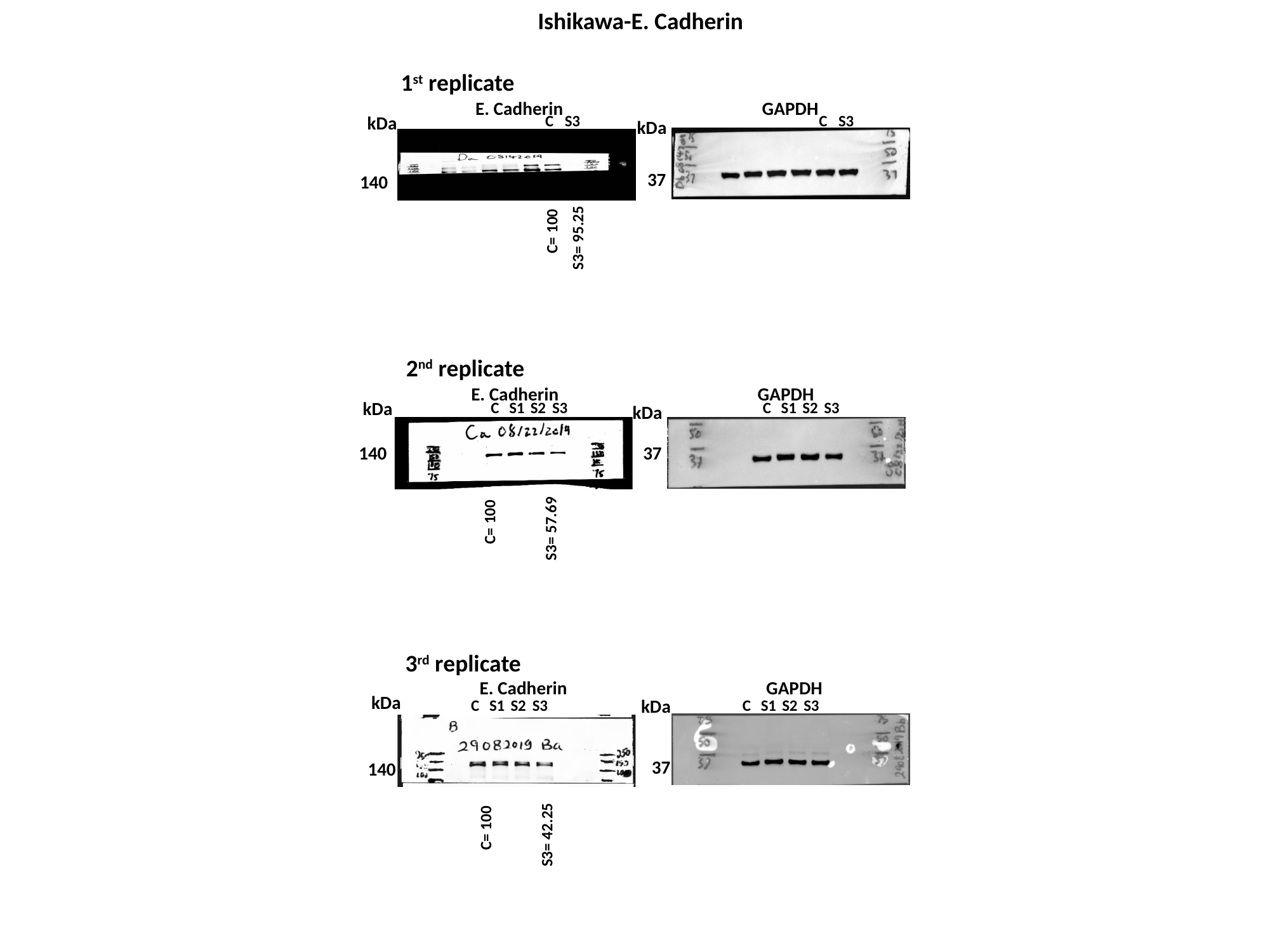

Ishikawa-E. Cadherin
1st replicate
E. Cadherin
GAPDH
C
S3
C
S3
kDa
kDa
37
140
C= 100
S3= 95.25
2nd replicate
E. Cadherin
GAPDH
kDa
S2
S1
C
S3
S2
S1
C
S3
kDa
140
37
C= 100
S3= 57.69
3rd replicate
E. Cadherin
GAPDH
kDa
kDa
S2
S1
C
S3
S2
S1
C
S3
37
140
C= 100
S3= 42.25

## Slide 25
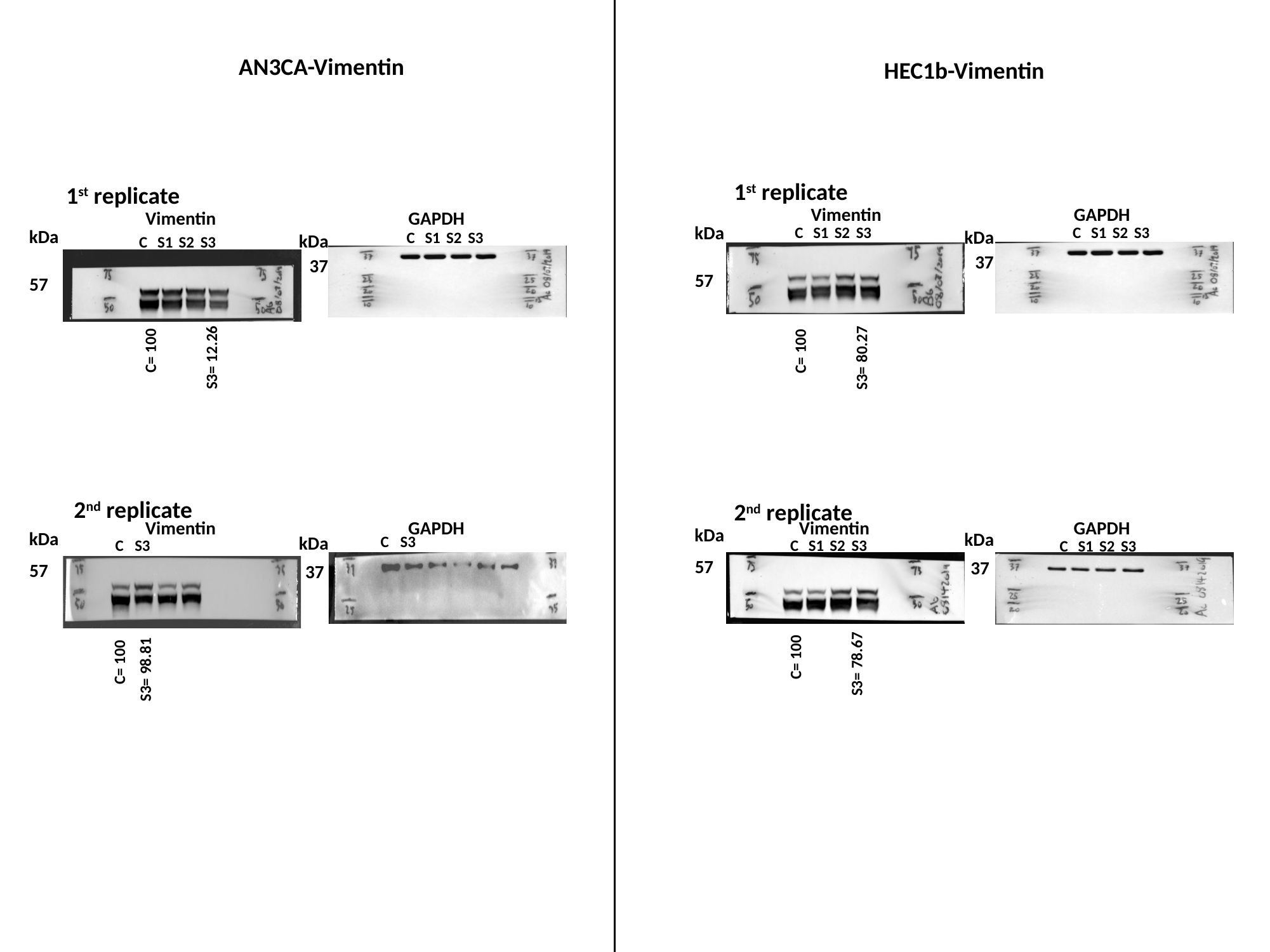

AN3CA-Vimentin
HEC1b-Vimentin
1st replicate
1st replicate
Vimentin
GAPDH
Vimentin
GAPDH
kDa
S2
S1
C
S3
S2
S1
C
S3
kDa
kDa
S2
S1
C
S3
kDa
S2
S1
C
S3
37
37
57
57
C= 100
C= 100
S3= 12.26
S3= 80.27
2nd replicate
2nd replicate
Vimentin
GAPDH
Vimentin
GAPDH
kDa
kDa
kDa
kDa
C
S3
C
S3
S2
S1
C
S3
S2
S1
C
S3
57
37
57
37
C= 100
C= 100
S3= 78.67
S3= 98.81

## Slide 26
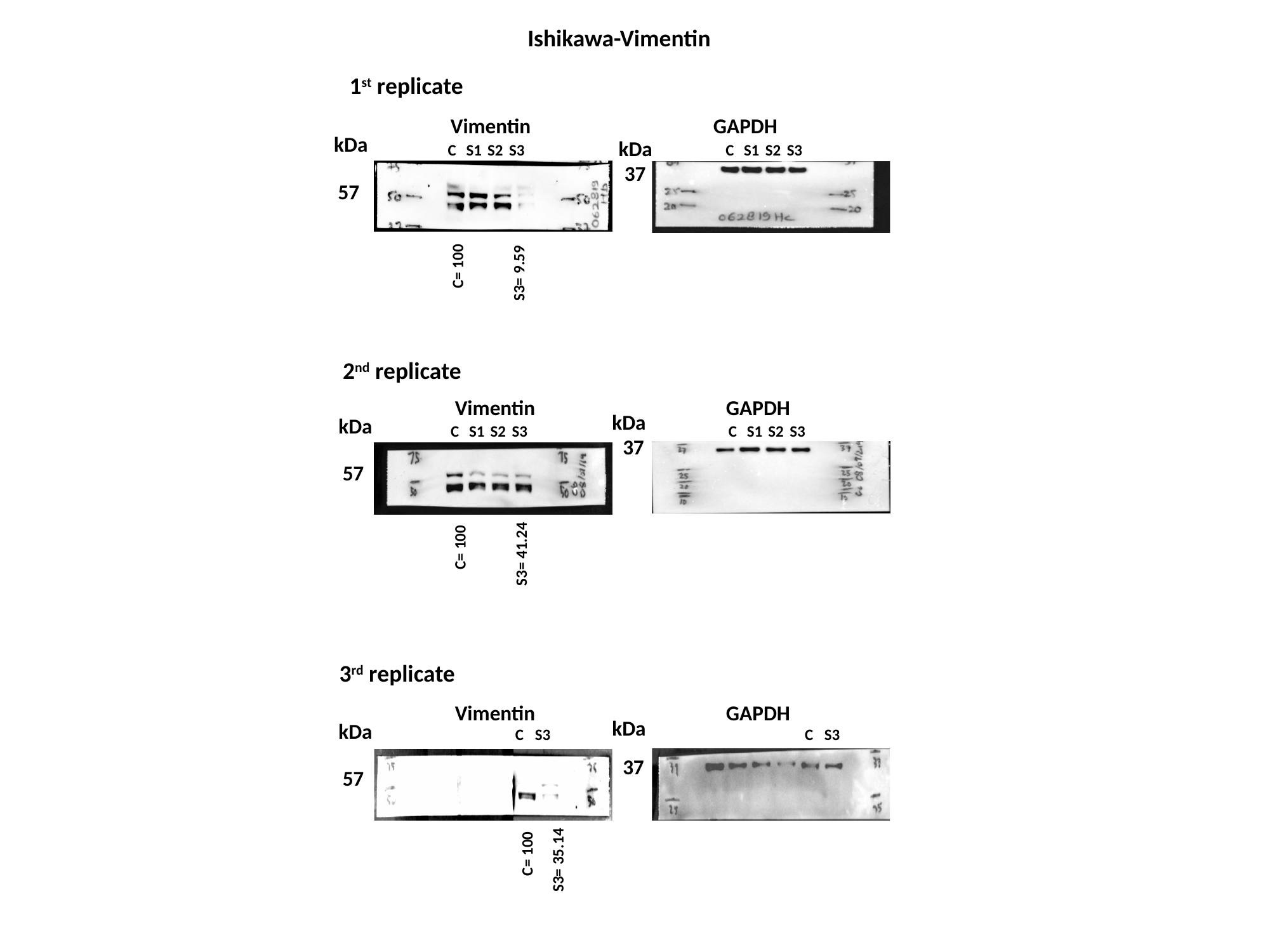

Ishikawa-Vimentin
1st replicate
Vimentin
GAPDH
kDa
kDa
S2
S1
C
S3
S2
S1
C
S3
37
57
C= 100
S3= 9.59
2nd replicate
Vimentin
GAPDH
kDa
kDa
S2
S1
C
S3
S2
S1
C
S3
37
57
C= 100
S3= 41.24
3rd replicate
Vimentin
GAPDH
kDa
kDa
C
S3
C
S3
37
57
C= 100
S3= 35.14

## Slide 27
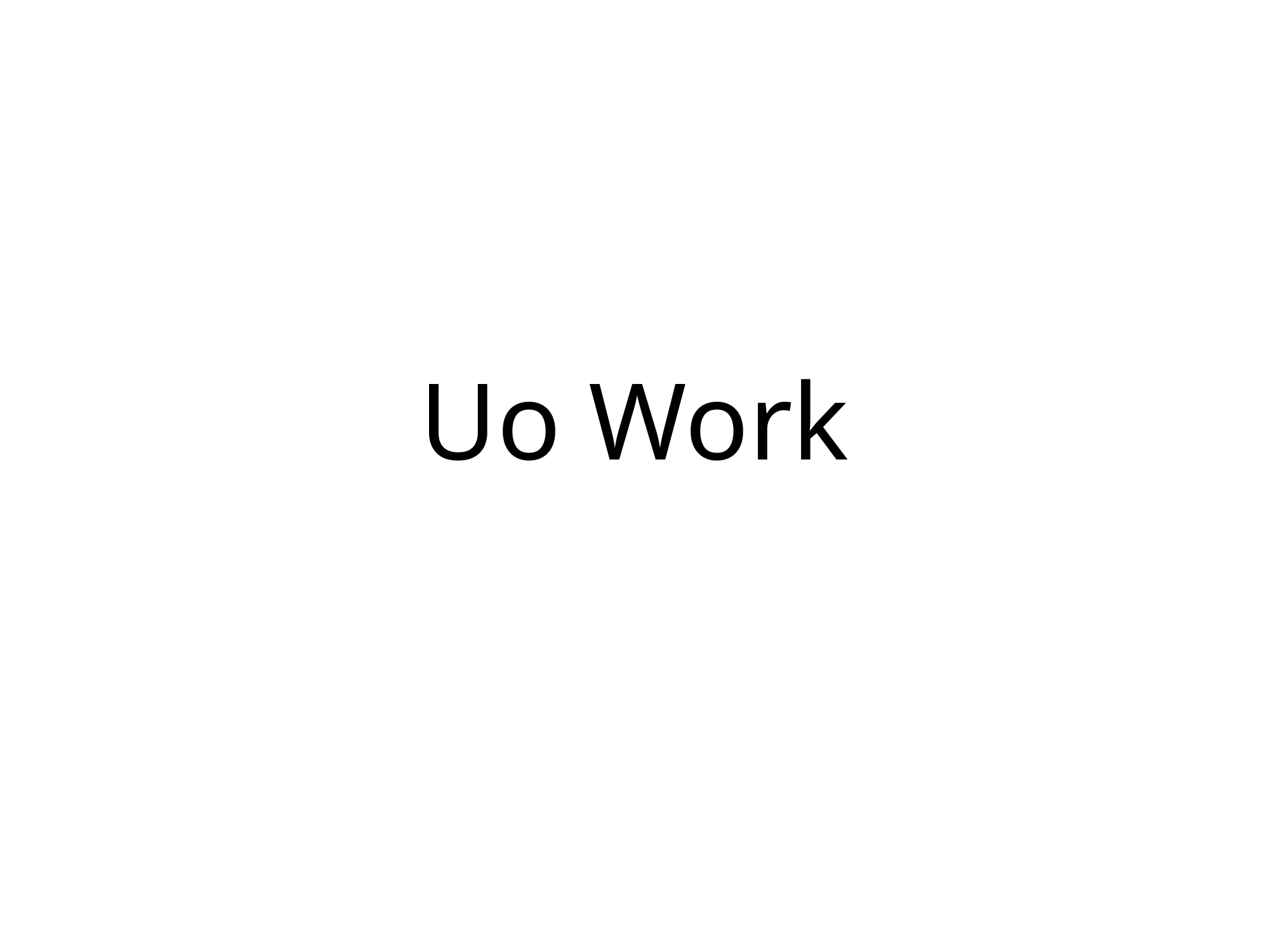

# Uo Work

## Slide 28
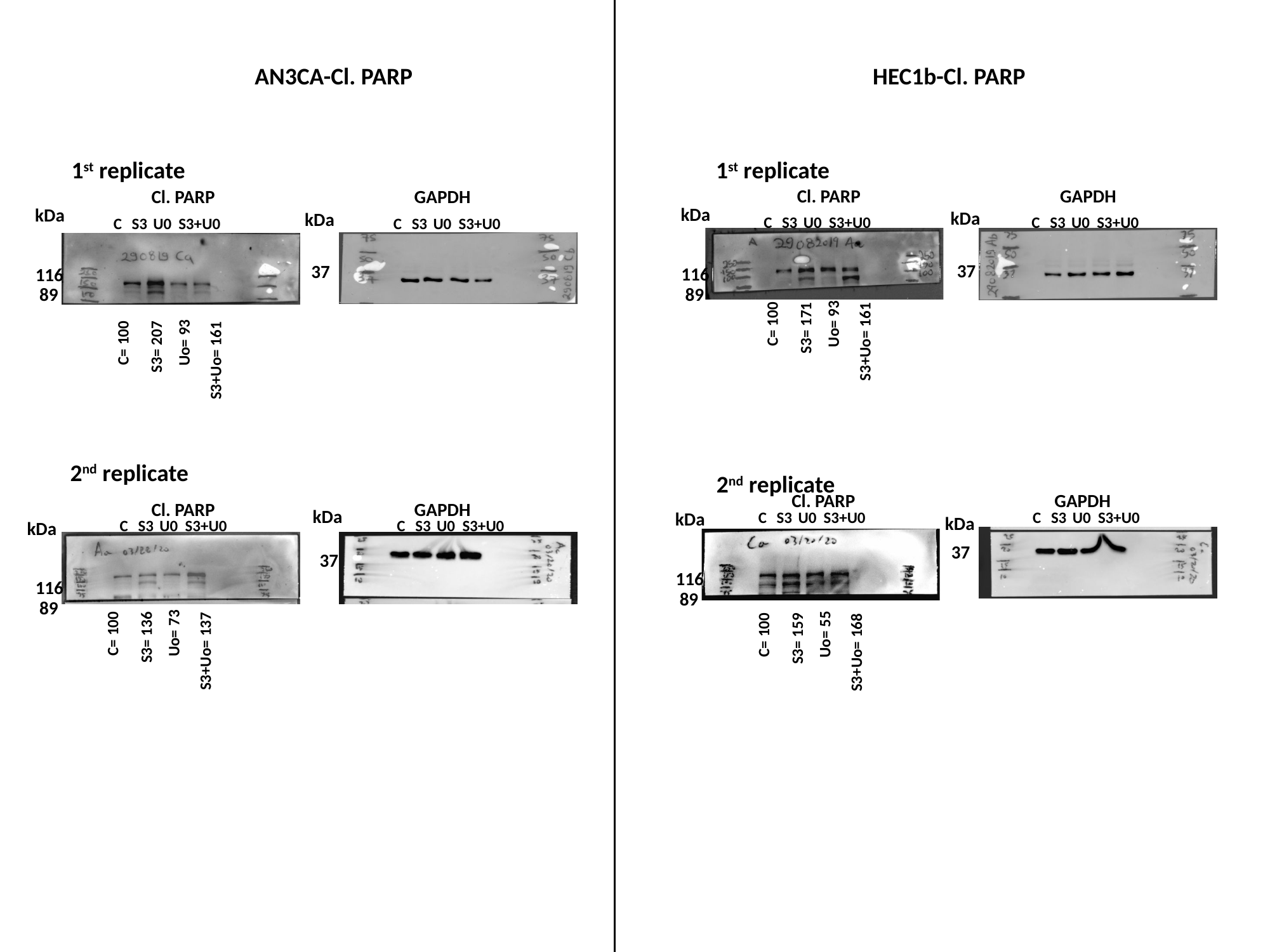

AN3CA-Cl. PARP
HEC1b-Cl. PARP
1st replicate
1st replicate
Cl. PARP
GAPDH
Cl. PARP
GAPDH
kDa
kDa
kDa
kDa
U0
S3+U0
S3
C
U0
S3+U0
S3
C
U0
S3+U0
S3
C
U0
S3+U0
S3
C
37
37
116
116
89
89
C= 100
Uo= 93
S3= 171
S3+Uo= 161
C= 100
Uo= 93
S3= 207
S3+Uo= 161
2nd replicate
2nd replicate
Cl. PARP
GAPDH
Cl. PARP
GAPDH
kDa
U0
S3+U0
S3
C
U0
S3+U0
S3
C
kDa
kDa
U0
S3+U0
S3
C
U0
S3+U0
S3
C
kDa
37
37
116
116
89
89
C= 100
Uo= 73
C= 100
S3= 136
Uo= 55
S3= 159
S3+Uo= 137
S3+Uo= 168

## Slide 29
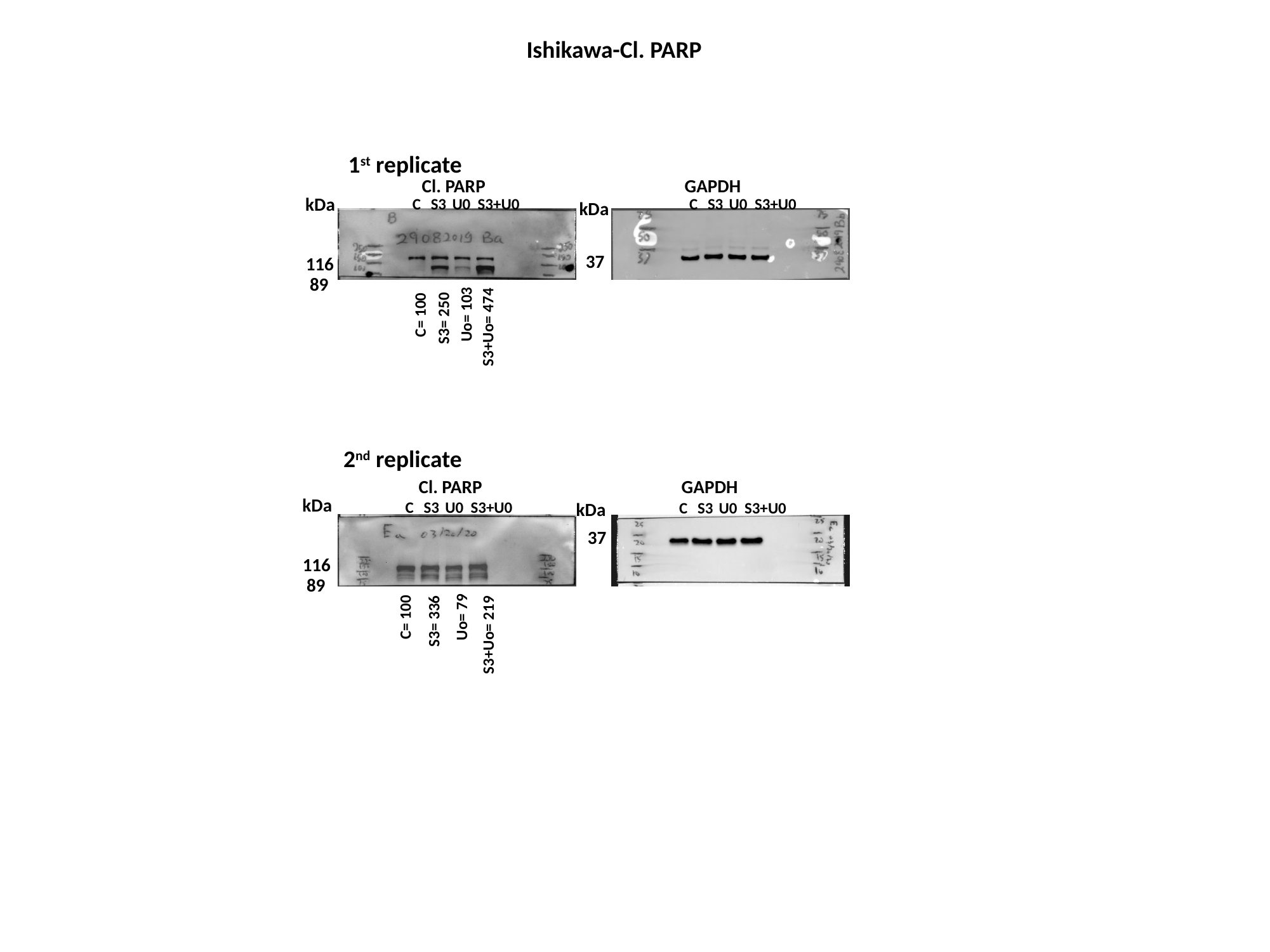

Ishikawa-Cl. PARP
1st replicate
Cl. PARP
GAPDH
kDa
U0
S3+U0
S3
C
U0
S3+U0
S3
C
kDa
37
116
89
C= 100
Uo= 103
S3= 250
S3+Uo= 474
2nd replicate
Cl. PARP
GAPDH
kDa
U0
S3+U0
S3
C
U0
S3+U0
S3
C
kDa
37
116
89
C= 100
Uo= 79
S3= 336
S3+Uo= 219

## Slide 30
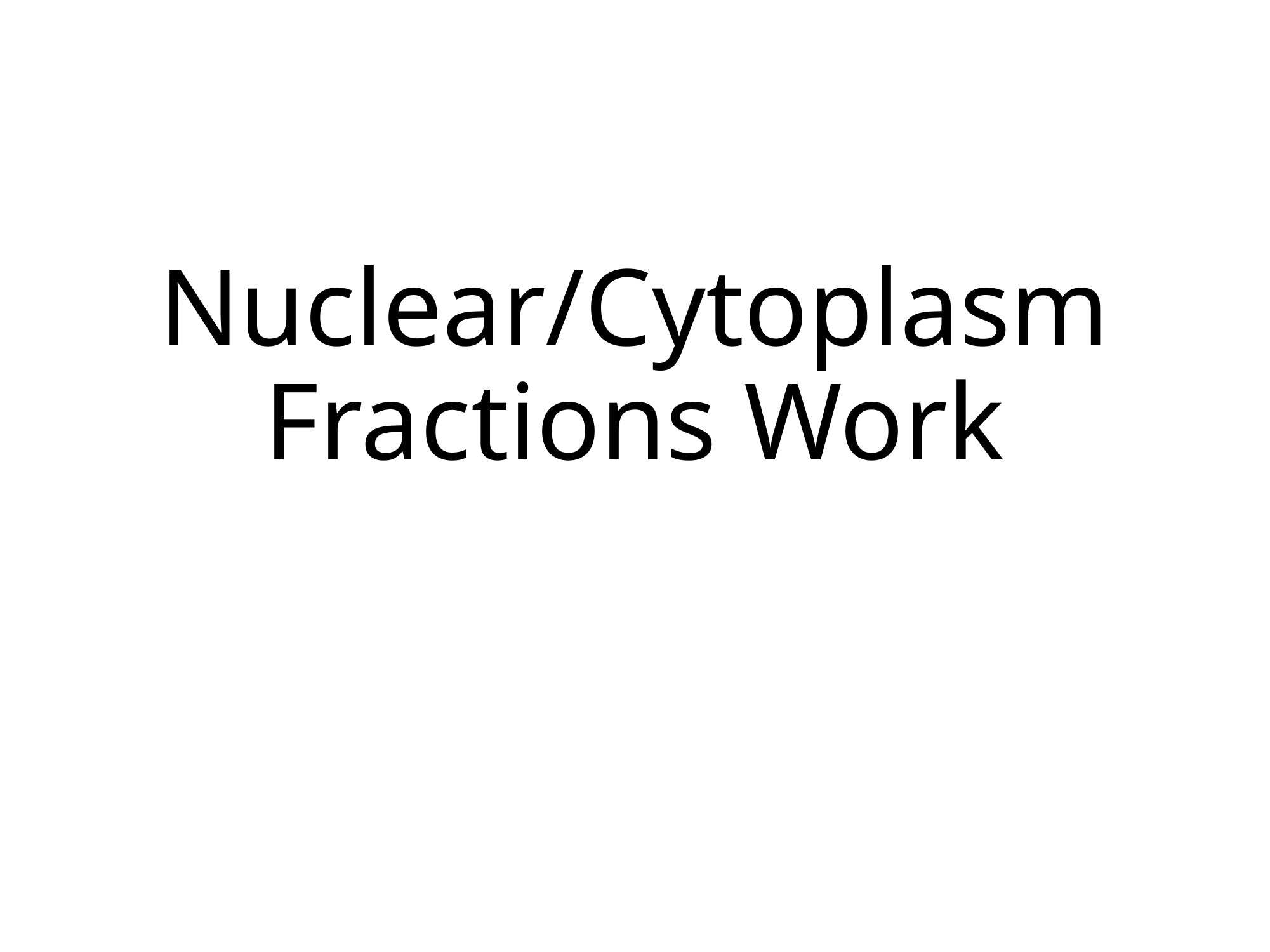

# Nuclear/Cytoplasm Fractions Work

## Slide 31
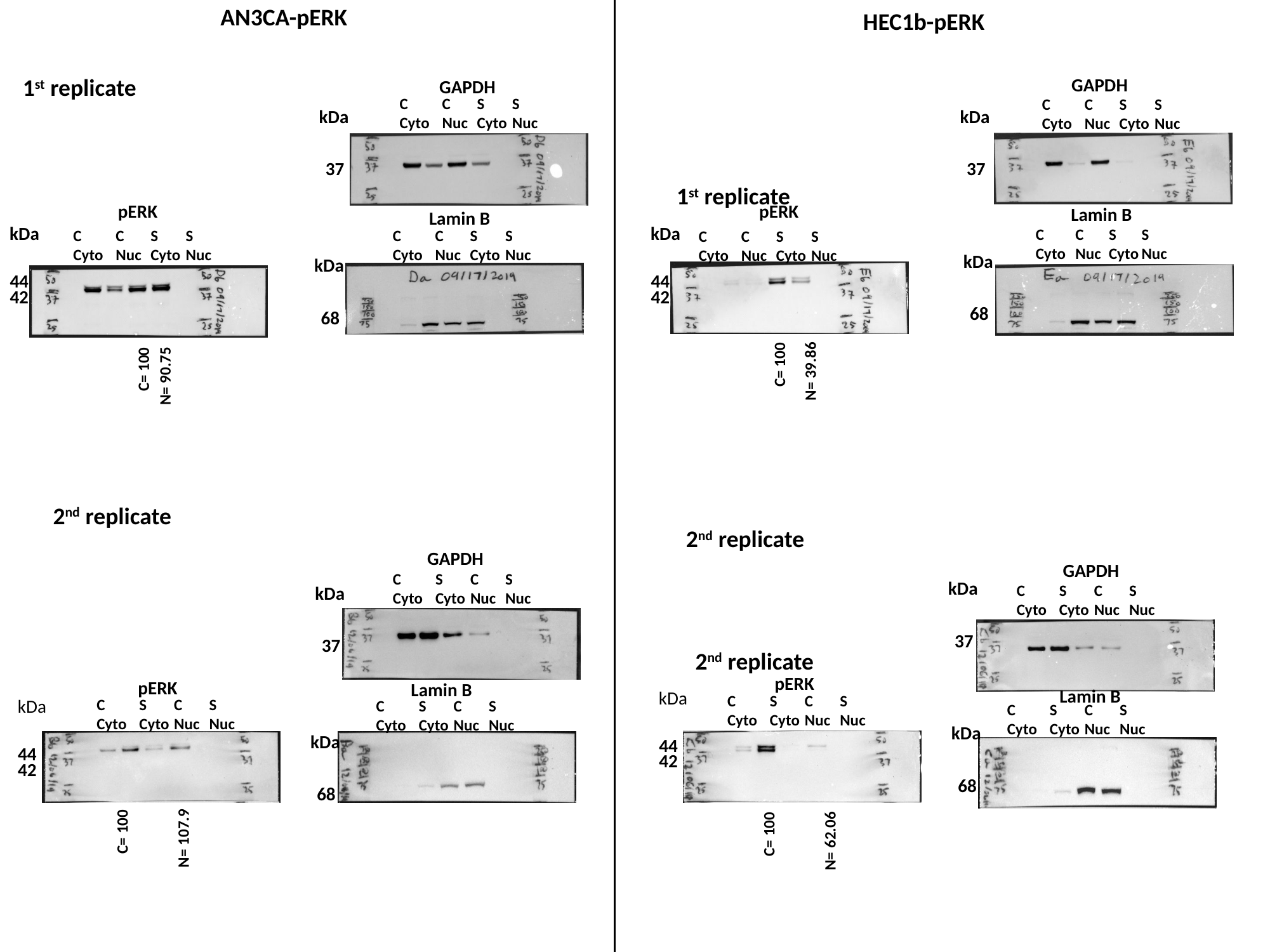

AN3CA-pERK
HEC1b-pERK
1st replicate
GAPDH
GAPDH
S
Cyto
C
Nuc
C
Cyto
S
Nuc
S
Cyto
C
Nuc
C
Cyto
S
Nuc
kDa
kDa
37
37
1st replicate
pERK
pERK
Lamin B
Lamin B
kDa
kDa
S
Cyto
C
Nuc
C
Cyto
S
Nuc
S
Cyto
C
Nuc
C
Cyto
S
Nuc
S
Cyto
C
Nuc
C
Cyto
S
Nuc
S
Cyto
C
Nuc
C
Cyto
S
Nuc
kDa
kDa
44
44
42
42
68
68
C= 100
C= 100
N= 39.86
N= 90.75
2nd replicate
2nd replicate
GAPDH
GAPDH
C
Nuc
S
Cyto
C
Cyto
S
Nuc
kDa
C
Nuc
S
Cyto
C
Cyto
S
Nuc
kDa
37
37
2nd replicate
pERK
pERK
Lamin B
Lamin B
kDa
C
Nuc
S
Cyto
C
Cyto
S
Nuc
kDa
C
Nuc
S
Cyto
C
Cyto
S
Nuc
C
Nuc
S
Cyto
C
Cyto
S
Nuc
C
Nuc
S
Cyto
C
Cyto
S
Nuc
kDa
kDa
44
44
42
42
68
68
C= 100
C= 100
N= 107.9
N= 62.06

## Slide 32
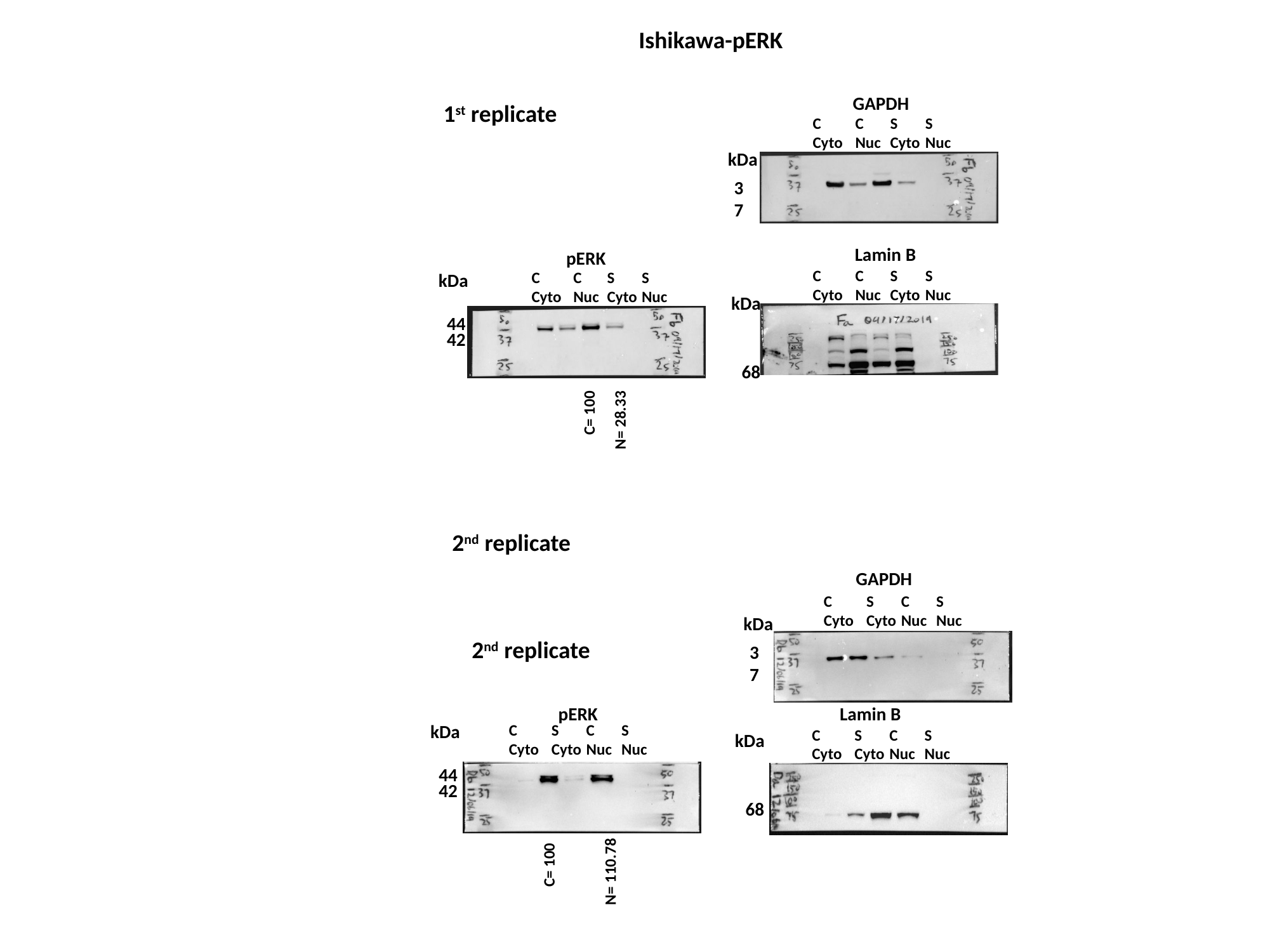

Ishikawa-pERK
GAPDH
1st replicate
S
Cyto
C
Nuc
C
Cyto
S
Nuc
kDa
37
Lamin B
pERK
S
Cyto
C
Nuc
C
Cyto
S
Nuc
S
Cyto
C
Nuc
C
Cyto
S
Nuc
kDa
kDa
44
42
68
C= 100
N= 28.33
2nd replicate
GAPDH
C
Nuc
S
Cyto
C
Cyto
S
Nuc
kDa
2nd replicate
37
pERK
Lamin B
kDa
C
Nuc
S
Cyto
C
Cyto
S
Nuc
C
Nuc
S
Cyto
C
Cyto
S
Nuc
kDa
44
42
68
C= 100
N= 110.78

## Slide 33
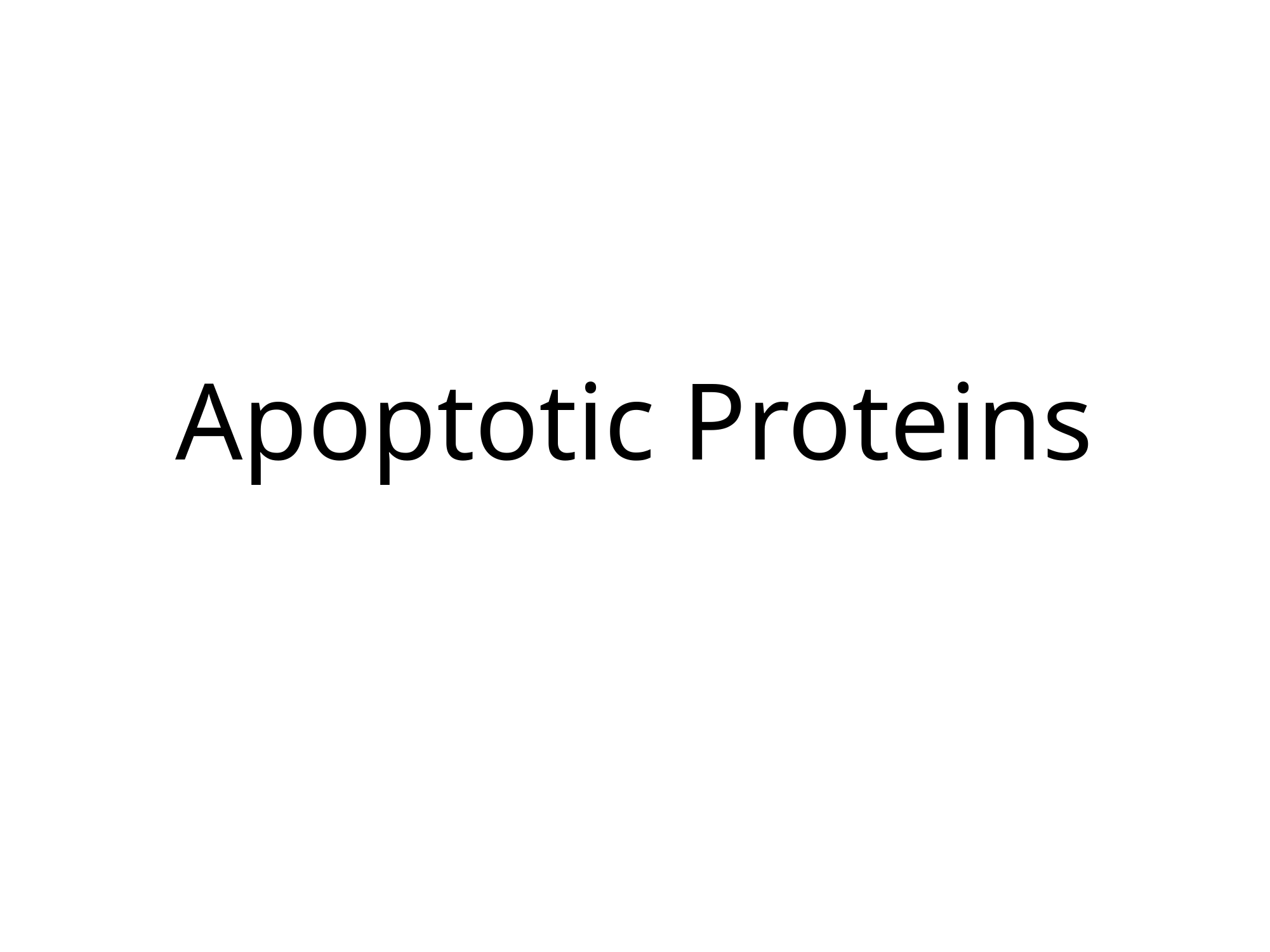

# Apoptotic Proteins

## Slide 34
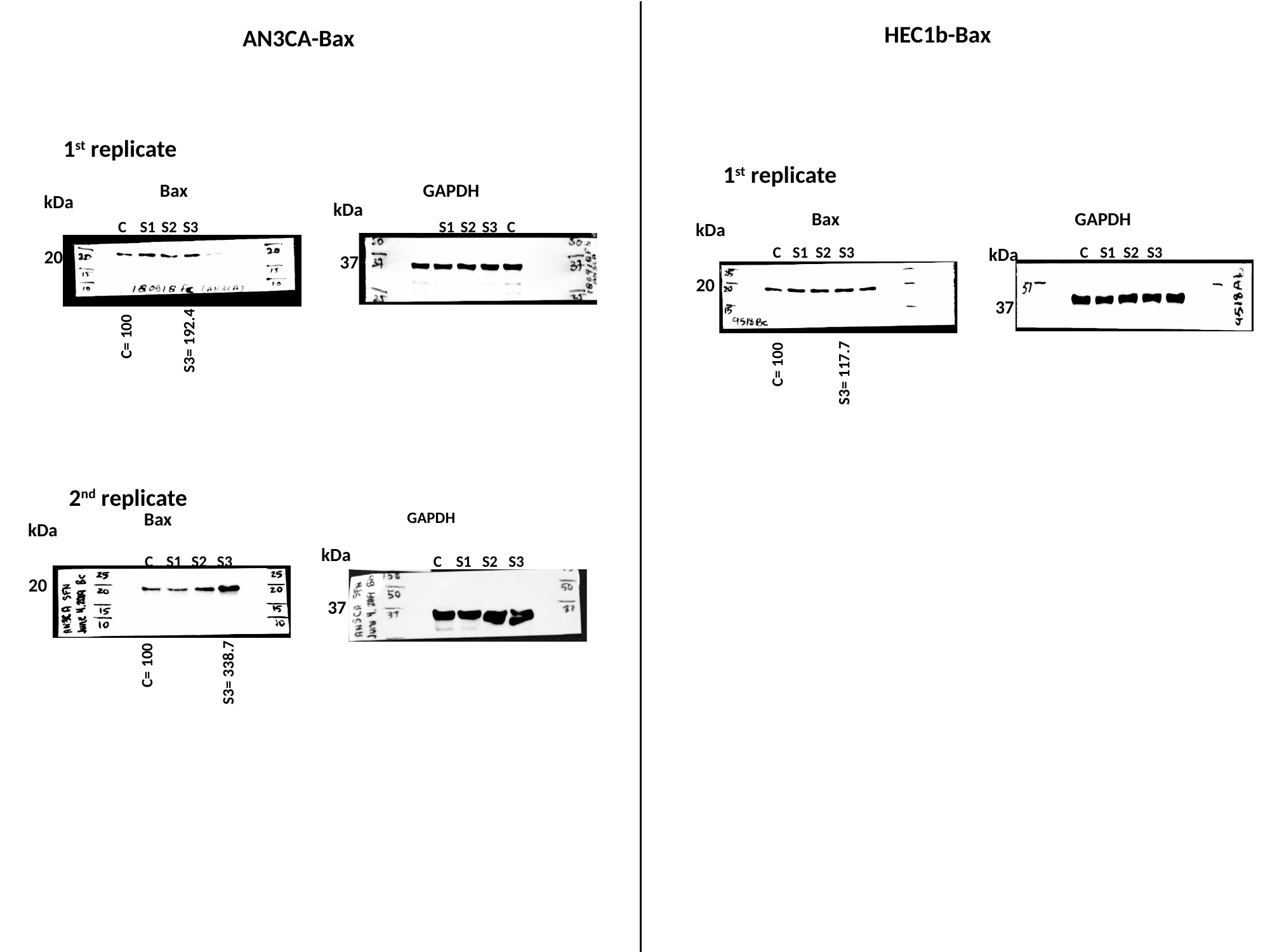

HEC1b-Bax
AN3CA-Bax
1st replicate
1st replicate
Bax
GAPDH
kDa
kDa
Bax
GAPDH
C
S1
S2
S3
C
S1
S2
S3
kDa
S2
S1
C
S3
S2
S1
C
S3
kDa
20
37
20
37
C= 100
S3= 192.4
C= 100
S3= 117.7
2nd replicate
Bax
GAPDH
kDa
kDa
S2
S1
C
S3
S2
S1
C
S3
20
37
C= 100
S3= 338.7

## Slide 35
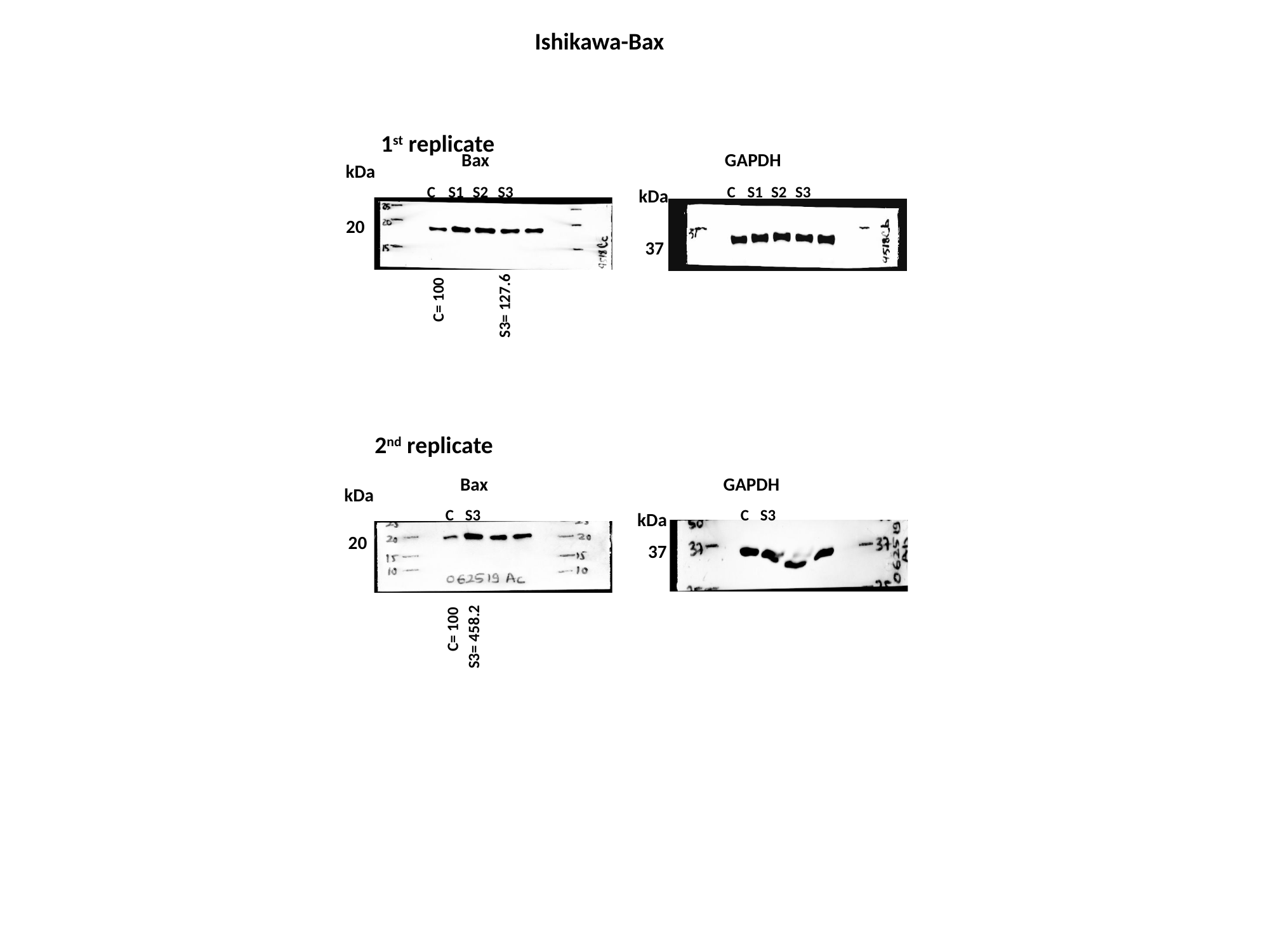

Ishikawa-Bax
1st replicate
Bax
GAPDH
kDa
S2
S1
C
S3
S2
S1
C
S3
kDa
20
37
C= 100
S3= 127.6
2nd replicate
Bax
GAPDH
kDa
C
S3
C
S3
kDa
20
37
C= 100
S3= 458.2

## Slide 36
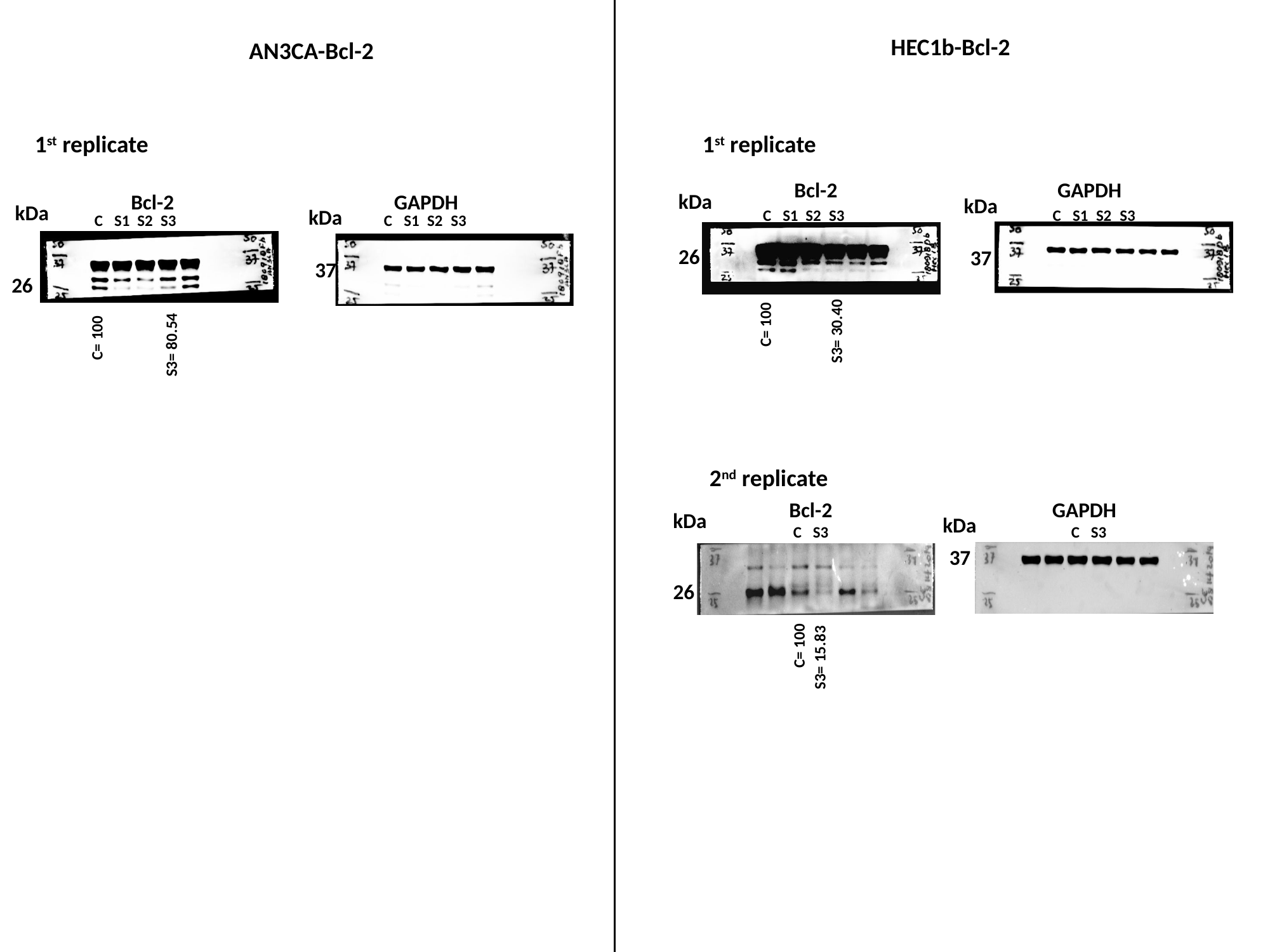

HEC1b-Bcl-2
AN3CA-Bcl-2
1st replicate
1st replicate
Bcl-2
GAPDH
kDa
Bcl-2
GAPDH
kDa
kDa
kDa
S2
S1
C
S3
S2
S1
C
S3
S2
S1
C
S3
S2
S1
C
S3
26
37
37
26
C= 100
S3= 30.40
C= 100
S3= 80.54
2nd replicate
Bcl-2
GAPDH
kDa
kDa
C
S3
C
S3
37
26
C= 100
S3= 15.83

## Slide 37
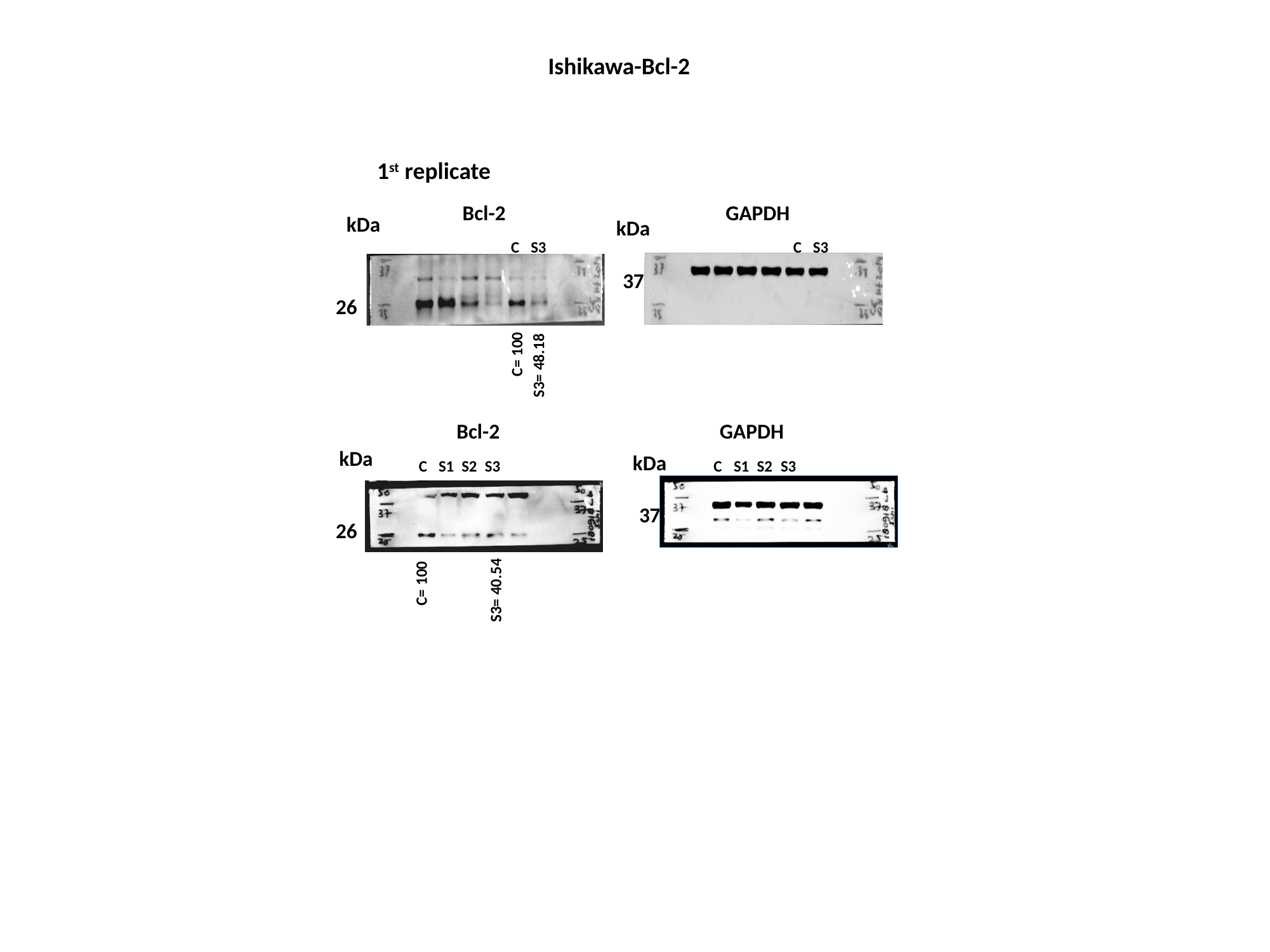

Ishikawa-Bcl-2
1st replicate
Bcl-2
GAPDH
kDa
kDa
C
S3
C
S3
37
26
C= 100
S3= 48.18
Bcl-2
GAPDH
kDa
kDa
S2
S1
C
S3
S2
S1
C
S3
37
26
C= 100
S3= 40.54

## Slide 38
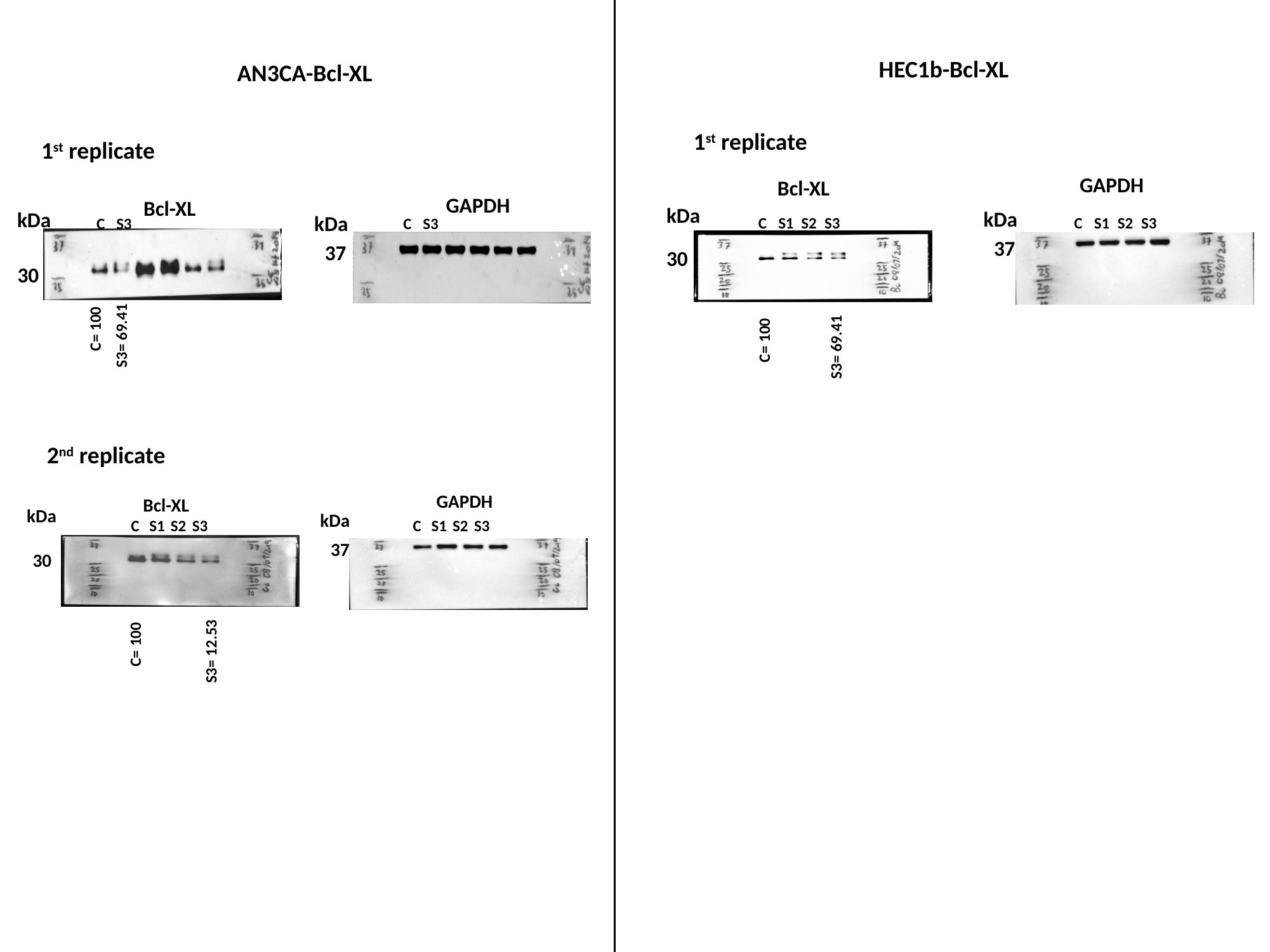

HEC1b-Bcl-XL
AN3CA-Bcl-XL
1st replicate
1st replicate
GAPDH
Bcl-XL
GAPDH
Bcl-XL
kDa
kDa
kDa
kDa
S2
S1
C
S3
S2
S1
C
S3
C
S3
C
S3
37
37
30
30
C= 100
S3= 69.41
C= 100
S3= 69.41
2nd replicate
GAPDH
Bcl-XL
kDa
kDa
S2
S1
C
S3
S2
S1
C
S3
37
30
C= 100
S3= 12.53

## Slide 39
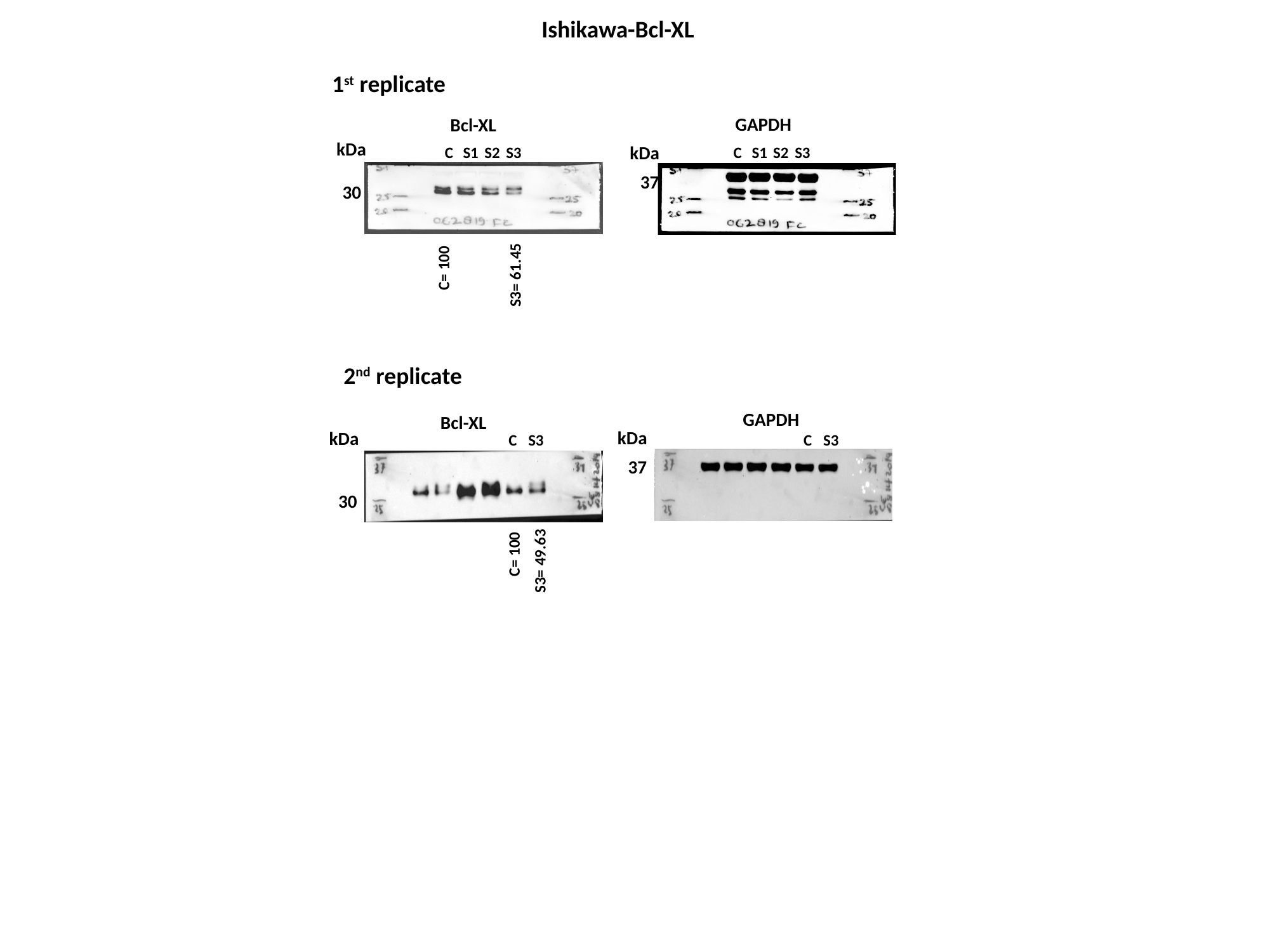

Ishikawa-Bcl-XL
1st replicate
GAPDH
Bcl-XL
kDa
kDa
S2
S1
C
S3
S2
S1
C
S3
37
30
C= 100
S3= 61.45
2nd replicate
GAPDH
Bcl-XL
kDa
kDa
C
S3
C
S3
37
30
C= 100
S3= 49.63

## Slide 40
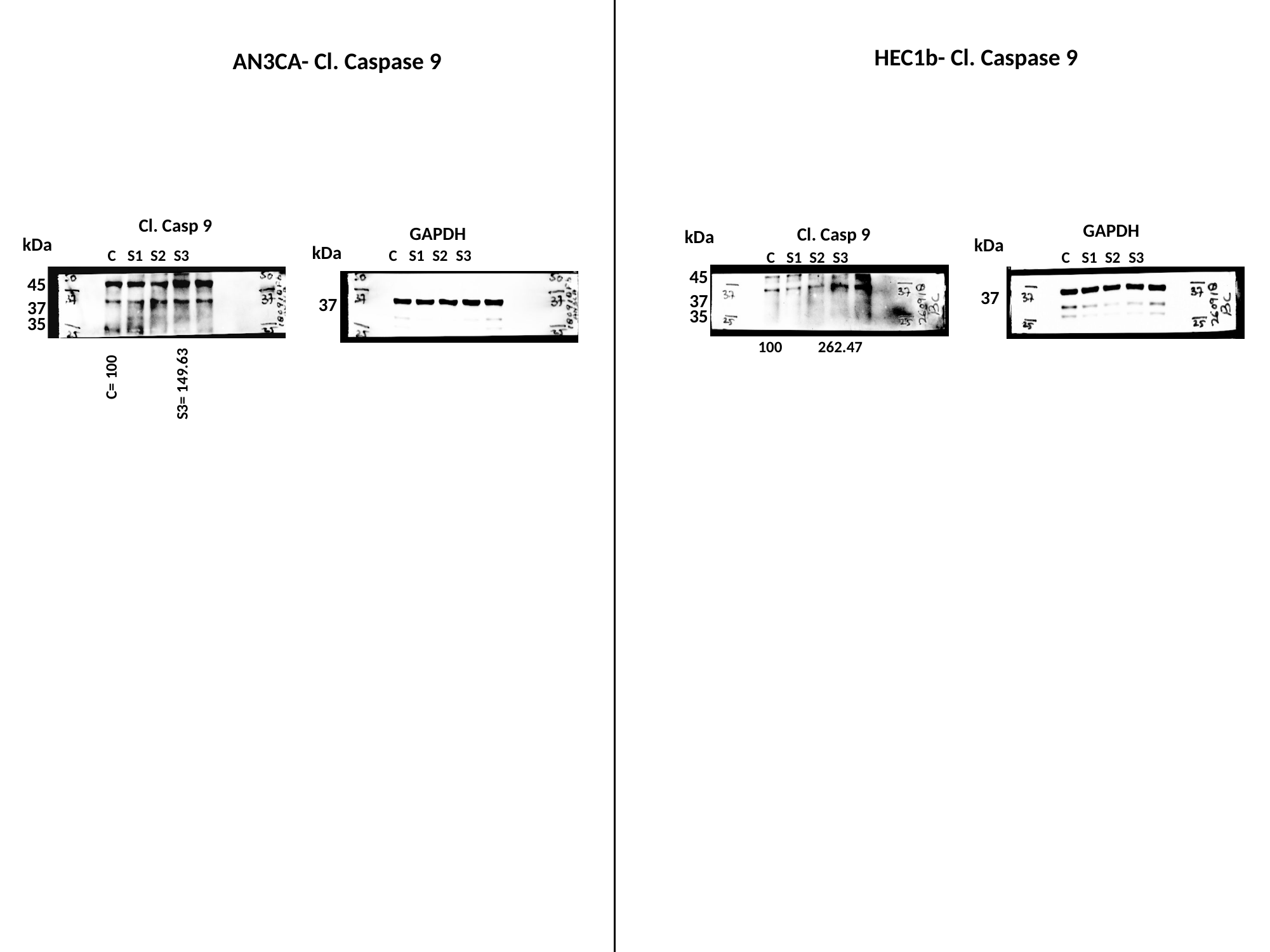

HEC1b- Cl. Caspase 9
AN3CA- Cl. Caspase 9
Cl. Casp 9
GAPDH
GAPDH
Cl. Casp 9
kDa
kDa
kDa
kDa
S2
S1
C
S3
S2
S1
C
S3
S2
S1
C
S3
S2
S1
C
S3
45
45
37
37
37
37
35
35
100
262.47
C= 100
S3= 149.63

## Slide 41
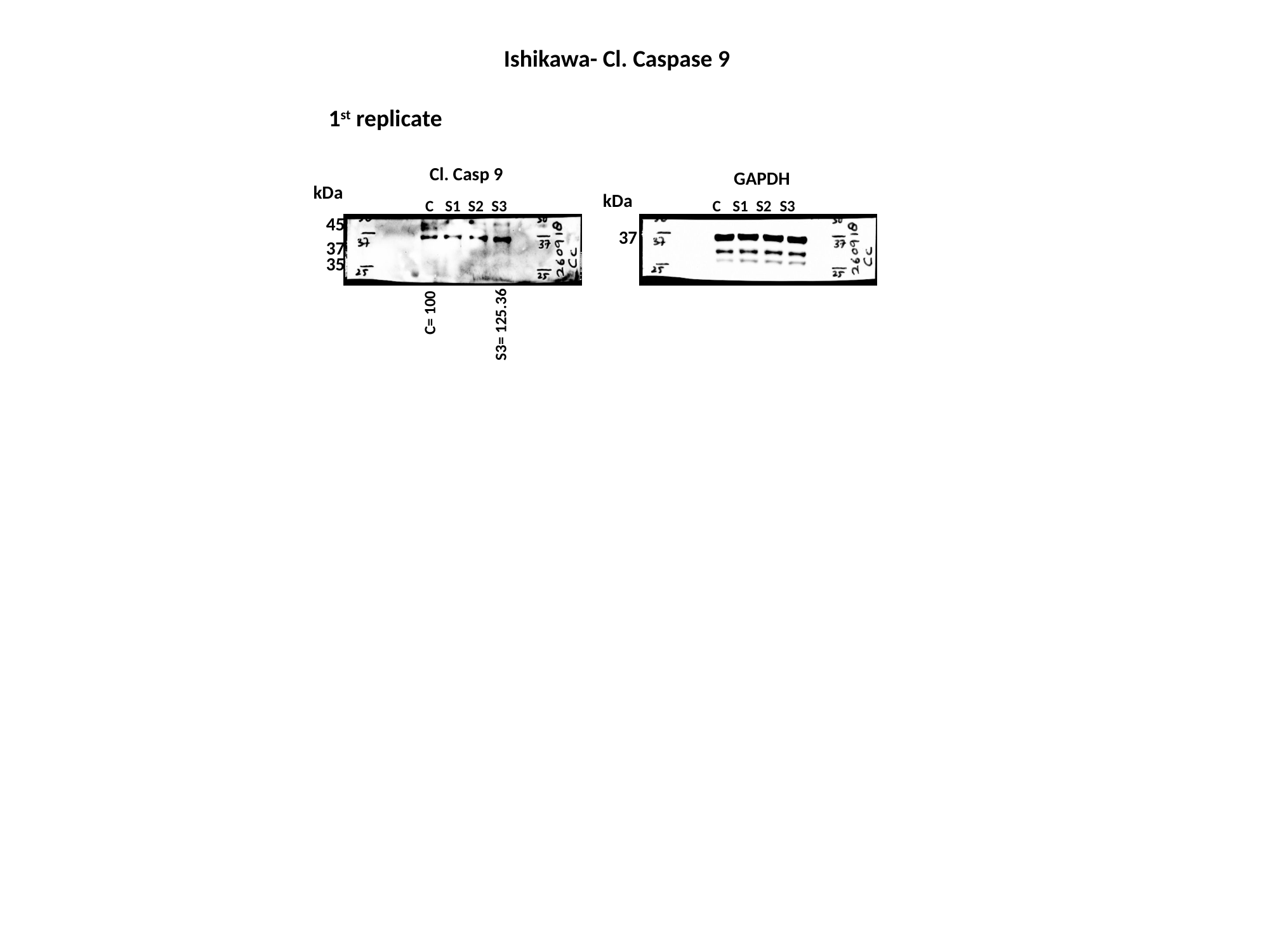

Ishikawa- Cl. Caspase 9
1st replicate
Cl. Casp 9
GAPDH
kDa
kDa
S2
S1
C
S3
S2
S1
C
S3
45
37
37
35
C= 100
S3= 125.36

## Slide 42
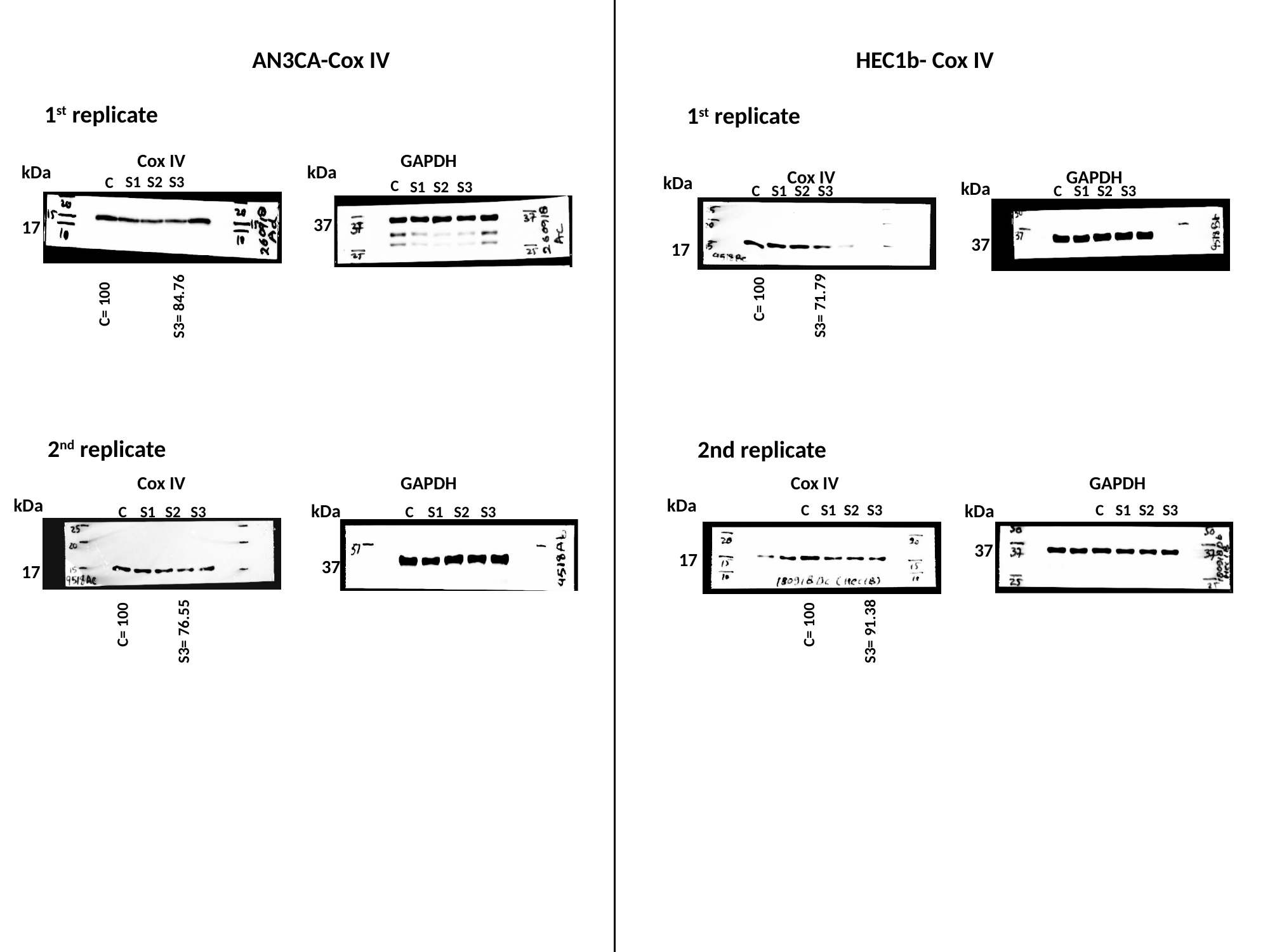

AN3CA-Cox IV
HEC1b- Cox IV
1st replicate
1st replicate
Cox IV
GAPDH
kDa
kDa
Cox IV
GAPDH
kDa
S1
S2
S3
C
C
S1
S2
S3
kDa
S2
S1
C
S3
S2
S1
C
S3
37
17
37
17
C= 100
C= 100
S3= 71.79
S3= 84.76
2nd replicate
2nd replicate
Cox IV
GAPDH
Cox IV
GAPDH
kDa
kDa
kDa
kDa
S2
S1
C
S3
S2
S1
C
S3
S2
S1
C
S3
S2
S1
C
S3
37
17
37
17
C= 100
C= 100
S3= 76.55
S3= 91.38

## Slide 43
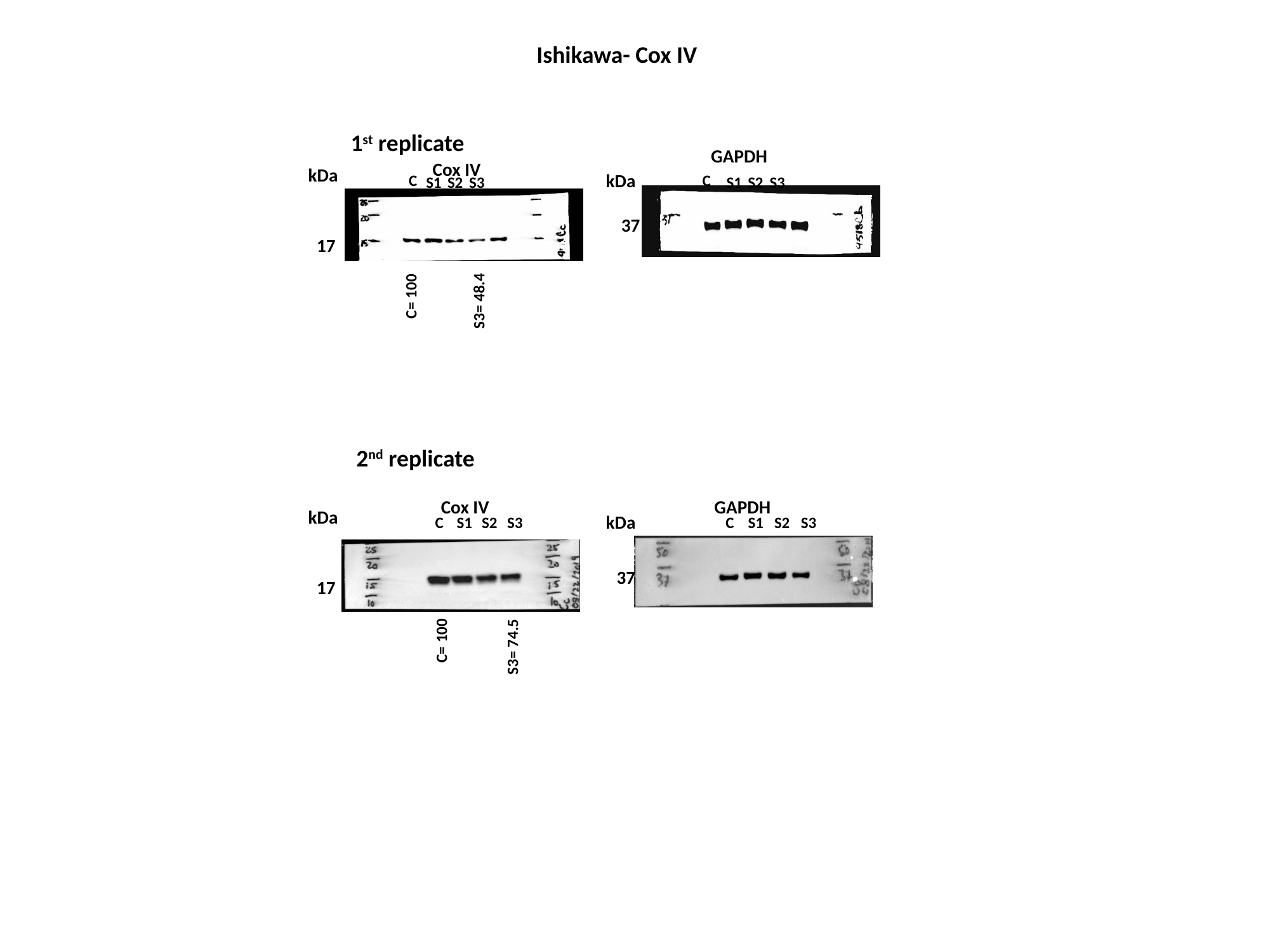

Ishikawa- Cox IV
1st replicate
GAPDH
Cox IV
kDa
kDa
C
S1
S2
S3
C
S1
S2
S3
37
17
C= 100
S3= 48.4
2nd replicate
Cox IV
GAPDH
kDa
kDa
S2
S1
C
S3
S2
S1
C
S3
37
17
C= 100
S3= 74.5

## Slide 44
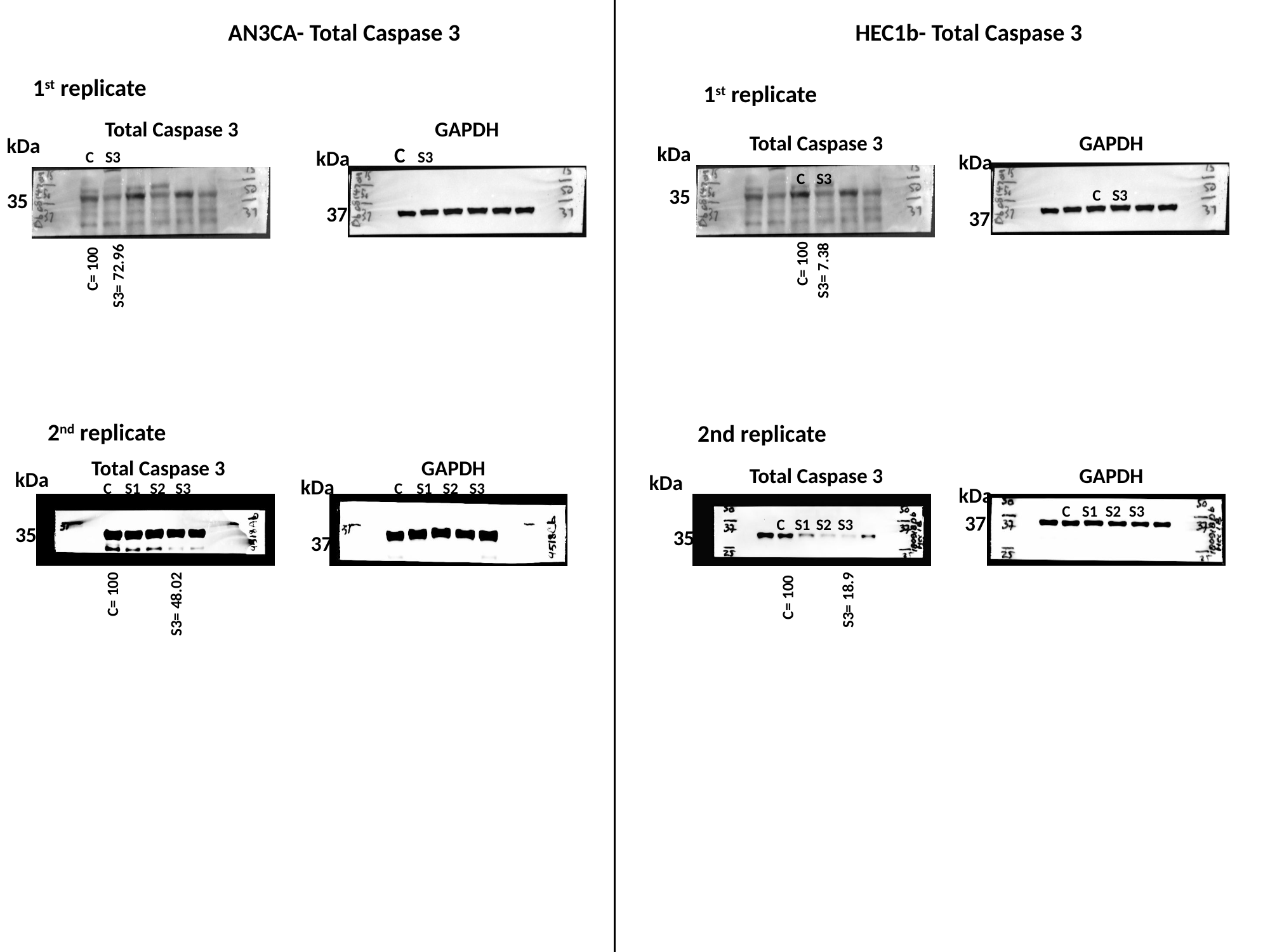

AN3CA- Total Caspase 3
HEC1b- Total Caspase 3
1st replicate
1st replicate
GAPDH
Total Caspase 3
GAPDH
Total Caspase 3
kDa
kDa
C
kDa
C
S3
S3
kDa
C
S3
35
C
S3
35
37
37
C= 100
C= 100
S3= 7.38
S3= 72.96
2nd replicate
2nd replicate
GAPDH
Total Caspase 3
GAPDH
Total Caspase 3
kDa
kDa
kDa
S2
S1
C
S3
S2
S1
C
S3
kDa
S2
S1
C
S3
37
S2
S1
C
S3
35
35
37
C= 100
C= 100
S3= 18.9
S3= 48.02

## Slide 45
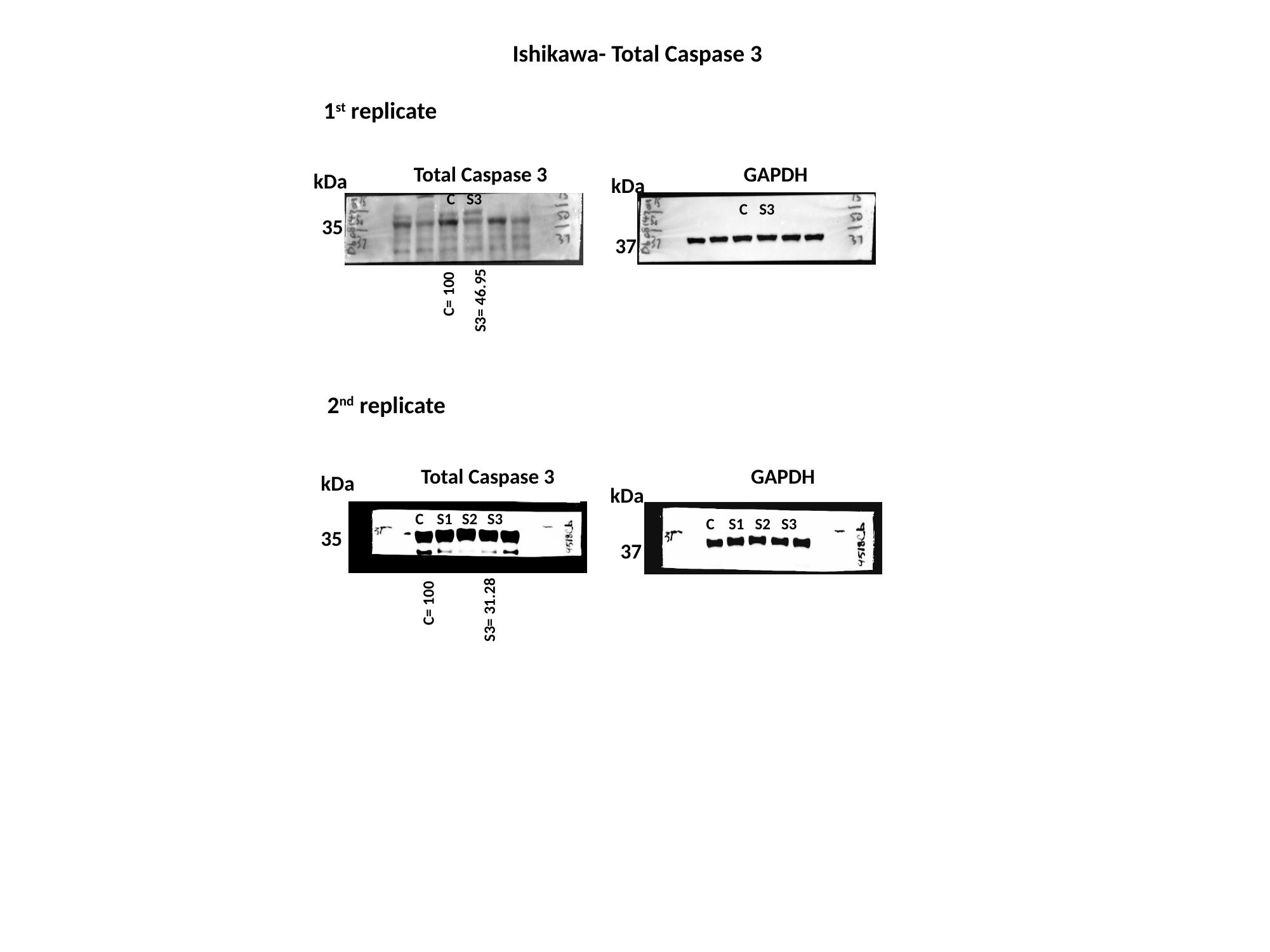

Ishikawa- Total Caspase 3
1st replicate
GAPDH
Total Caspase 3
kDa
kDa
C
S3
C
S3
35
37
C= 100
S3= 46.95
2nd replicate
GAPDH
Total Caspase 3
kDa
kDa
S2
S1
C
S3
S2
S1
C
S3
35
37
C= 100
S3= 31.28

## Slide 46
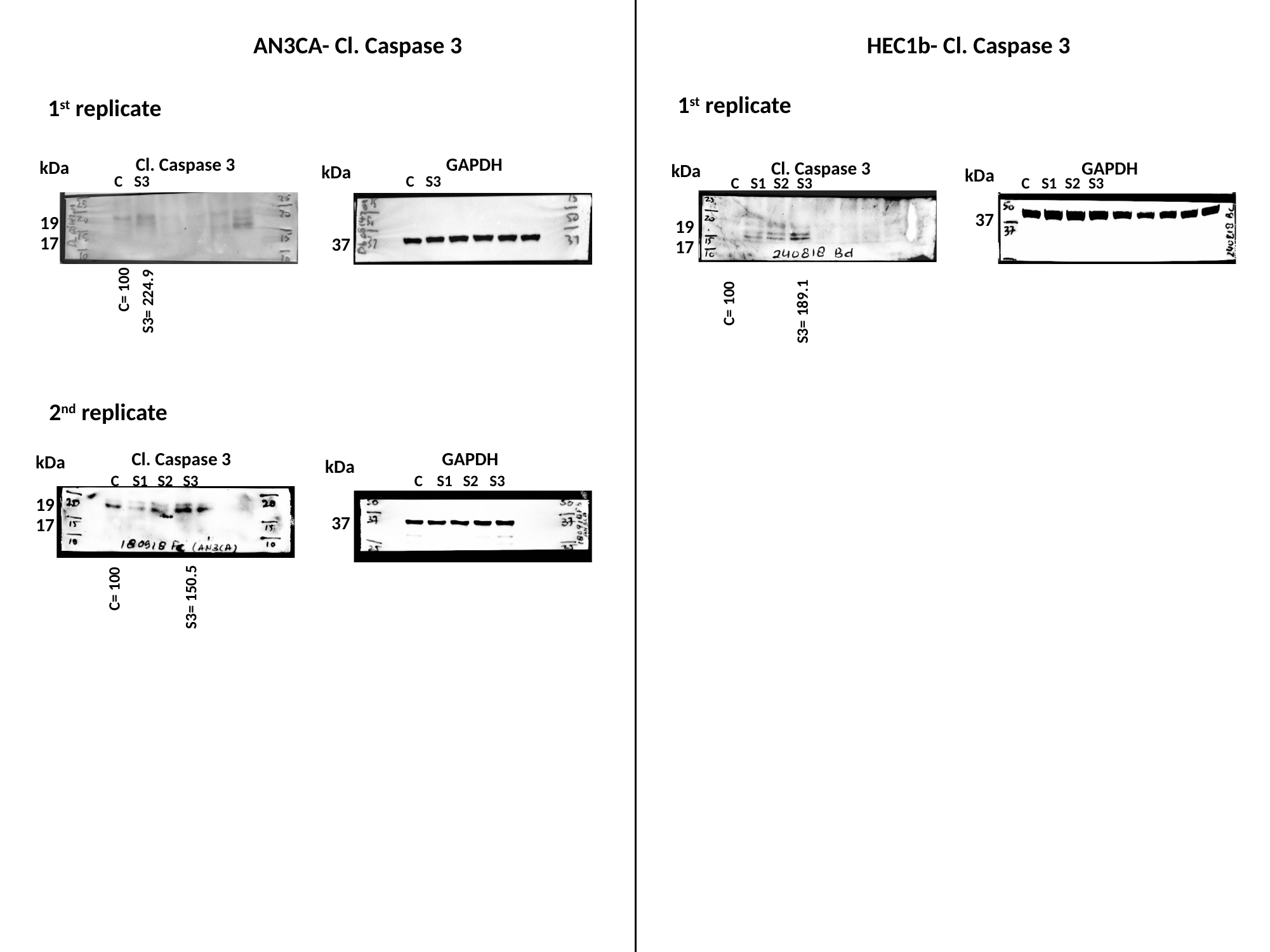

AN3CA- Cl. Caspase 3
HEC1b- Cl. Caspase 3
1st replicate
1st replicate
GAPDH
Cl. Caspase 3
kDa
GAPDH
Cl. Caspase 3
kDa
kDa
kDa
C
S3
C
S3
S2
S1
C
S3
S2
S1
C
S3
37
19
19
17
37
17
C= 100
S3= 224.9
C= 100
S3= 189.1
2nd replicate
GAPDH
Cl. Caspase 3
kDa
kDa
S2
S1
C
S3
S2
S1
C
S3
19
37
17
C= 100
S3= 150.5

## Slide 47
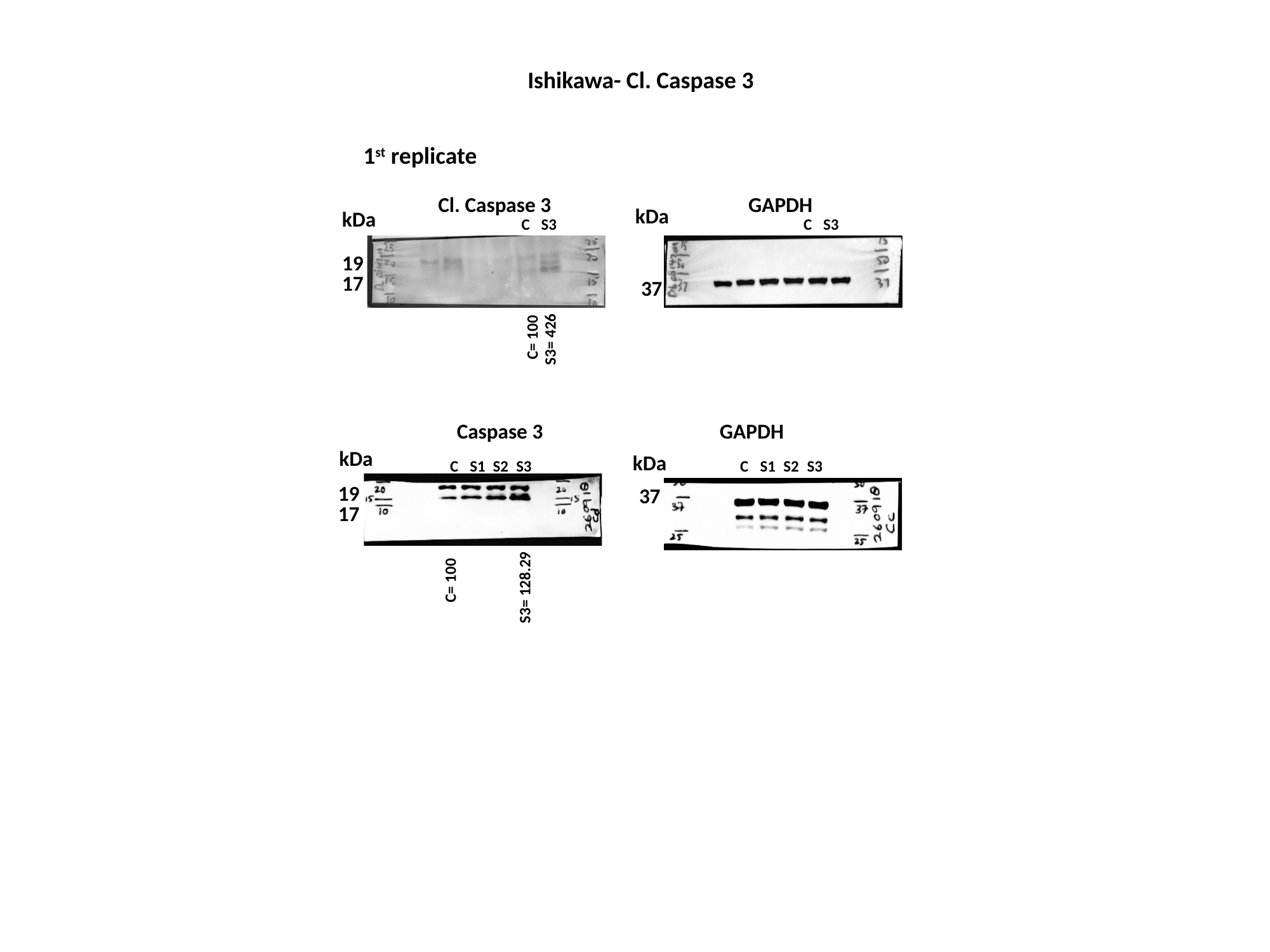

Ishikawa- Cl. Caspase 3
1st replicate
GAPDH
Cl. Caspase 3
kDa
kDa
C
S3
C
S3
19
17
37
C= 100
S3= 426
Caspase 3
GAPDH
kDa
kDa
S2
S1
C
S3
S2
S1
C
S3
19
37
17
C= 100
S3= 128.29

## Slide 48
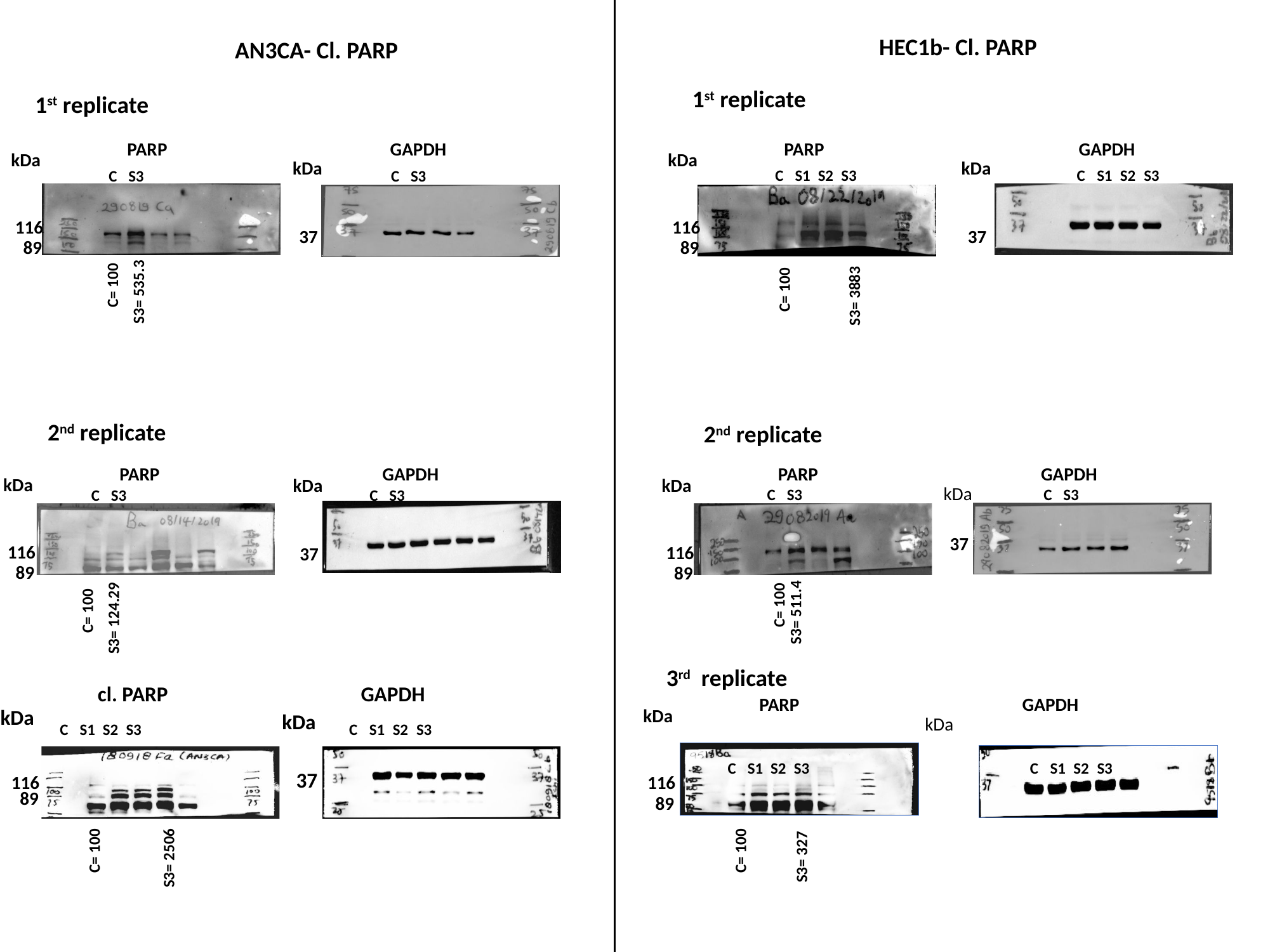

HEC1b- Cl. PARP
AN3CA- Cl. PARP
1st replicate
1st replicate
GAPDH
PARP
GAPDH
PARP
kDa
kDa
kDa
kDa
S2
S1
C
S3
S2
S1
C
S3
C
S3
C
S3
116
116
37
37
89
89
C= 100
C= 100
S3= 535.3
S3= 3883
2nd replicate
2nd replicate
PARP
GAPDH
PARP
GAPDH
kDa
kDa
kDa
kDa
C
S3
C
S3
C
S3
C
S3
37
116
116
37
89
89
C= 100
C= 100
S3= 511.4
S3= 124.29
3rd replicate
cl. PARP
GAPDH
PARP
GAPDH
kDa
kDa
kDa
kDa
S2
S1
C
S3
S2
S1
C
S3
S2
S1
C
S3
S2
S1
C
S3
37
116
116
89
89
C= 100
C= 100
S3= 327
S3= 2506

## Slide 49
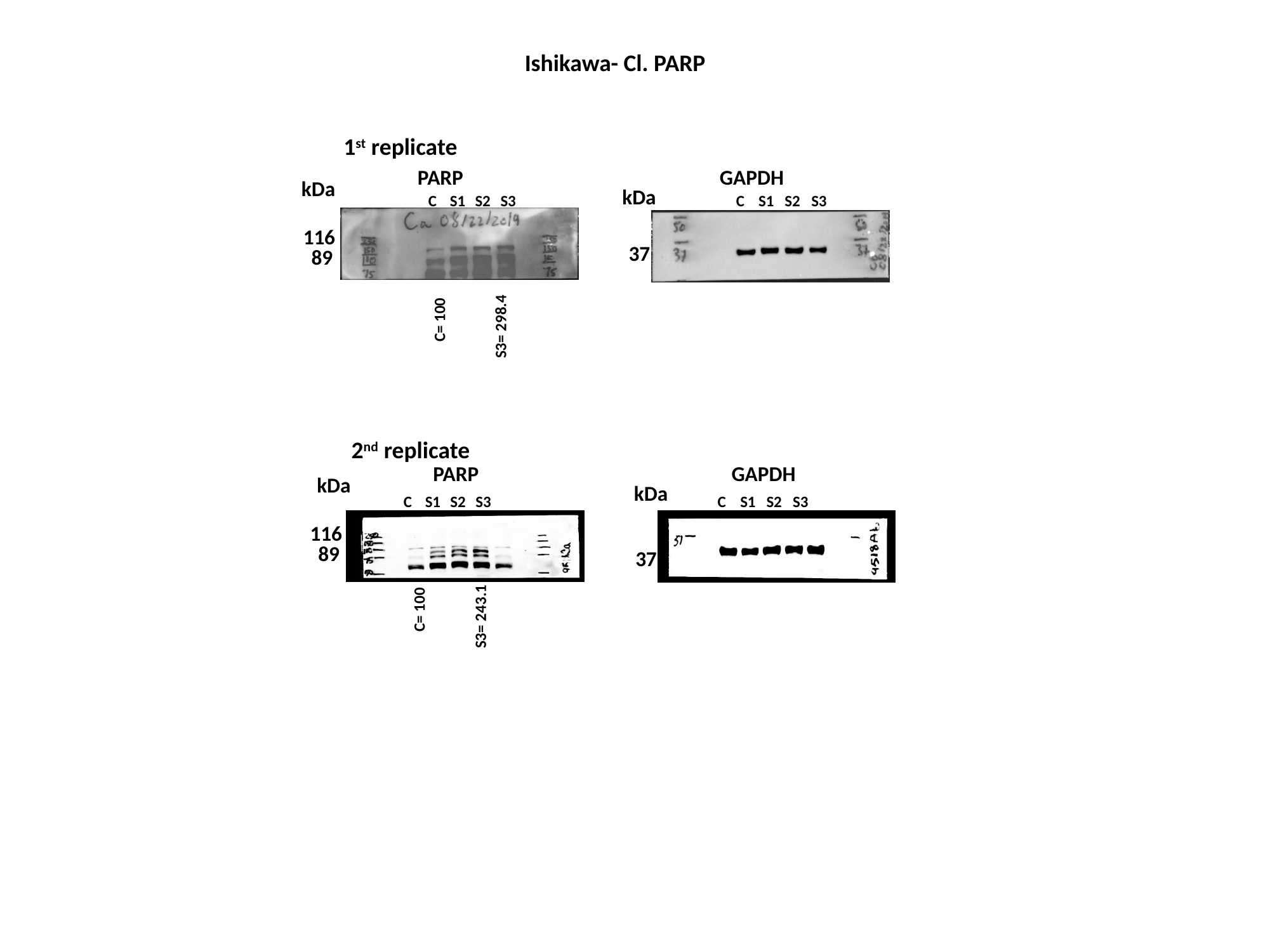

Ishikawa- Cl. PARP
1st replicate
PARP
GAPDH
kDa
kDa
S2
S1
C
S3
S2
S1
C
S3
116
37
89
C= 100
S3= 298.4
2nd replicate
PARP
GAPDH
kDa
kDa
S2
S1
C
S3
S2
S1
C
S3
116
89
37
C= 100
S3= 243.1

## Slide 50
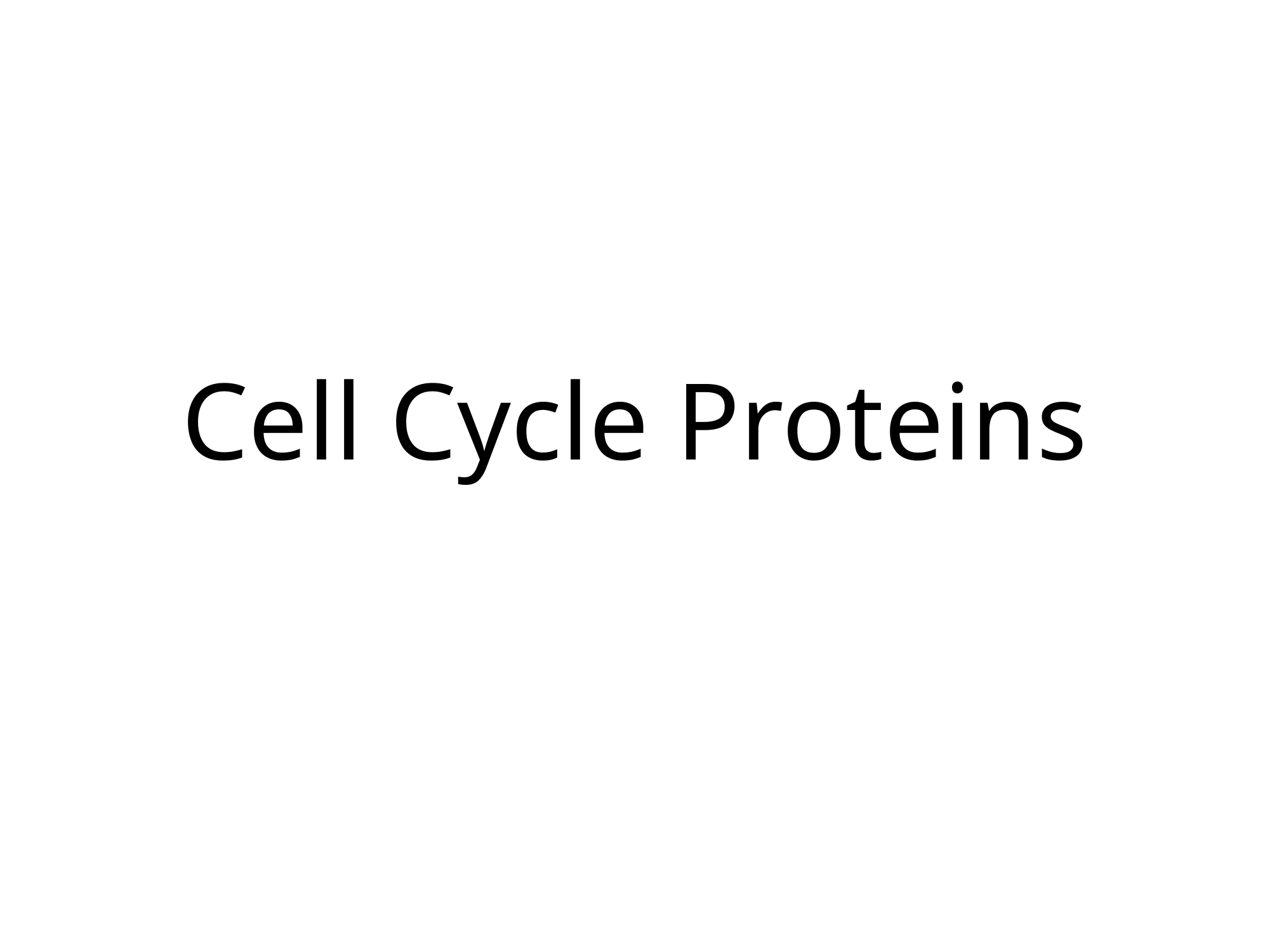

# Cell Cycle Proteins

## Slide 51
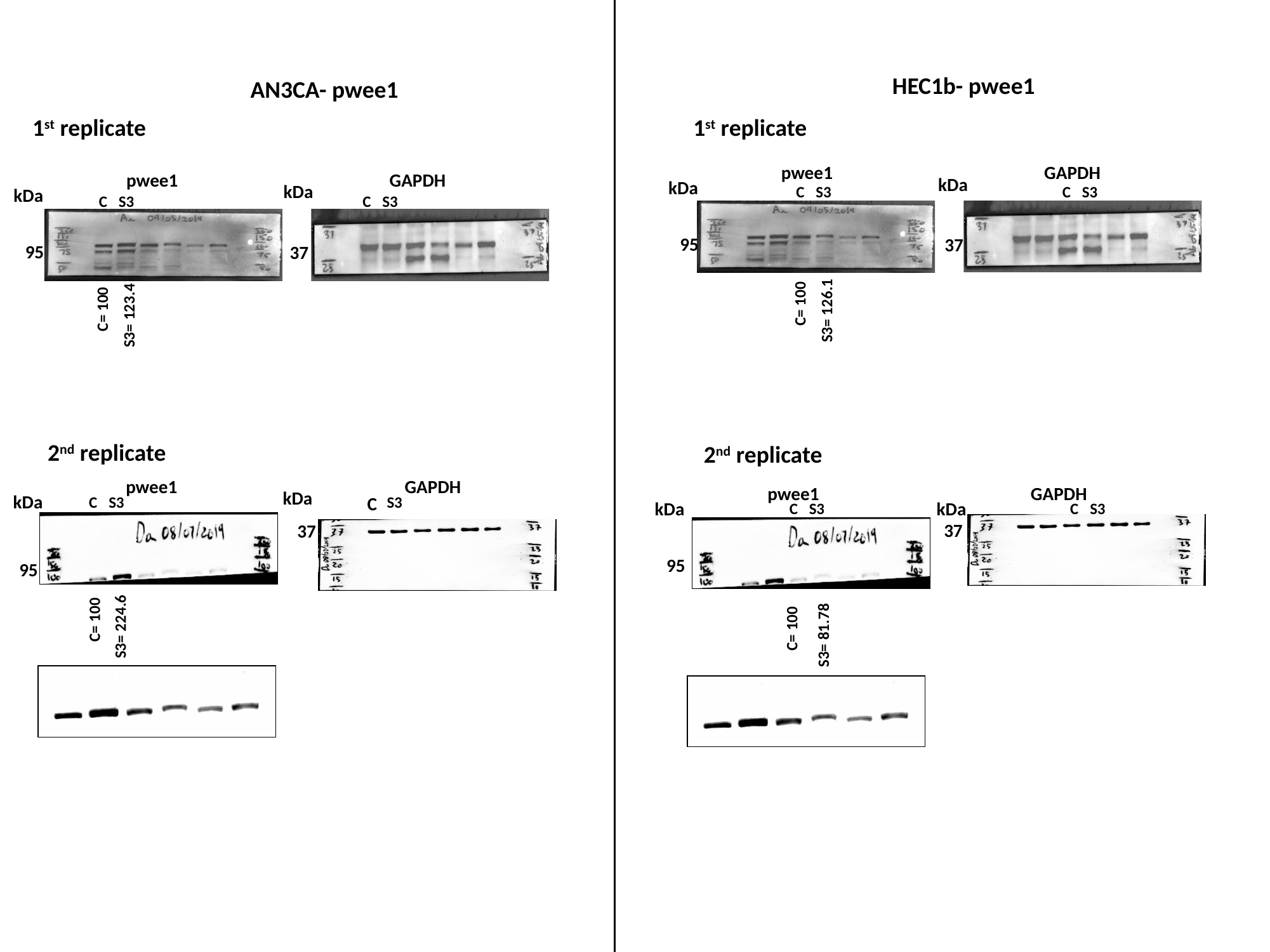

HEC1b- pwee1
AN3CA- pwee1
1st replicate
1st replicate
pwee1
GAPDH
pwee1
GAPDH
kDa
kDa
kDa
C
S3
C
S3
kDa
C
S3
C
S3
95
37
95
37
C= 100
C= 100
S3= 126.1
S3= 123.4
2nd replicate
2nd replicate
pwee1
GAPDH
pwee1
GAPDH
kDa
kDa
C
S3
C
S3
kDa
kDa
C
S3
C
S3
37
37
95
95
C= 100
S3= 224.6
C= 100
S3= 81.78

## Slide 52
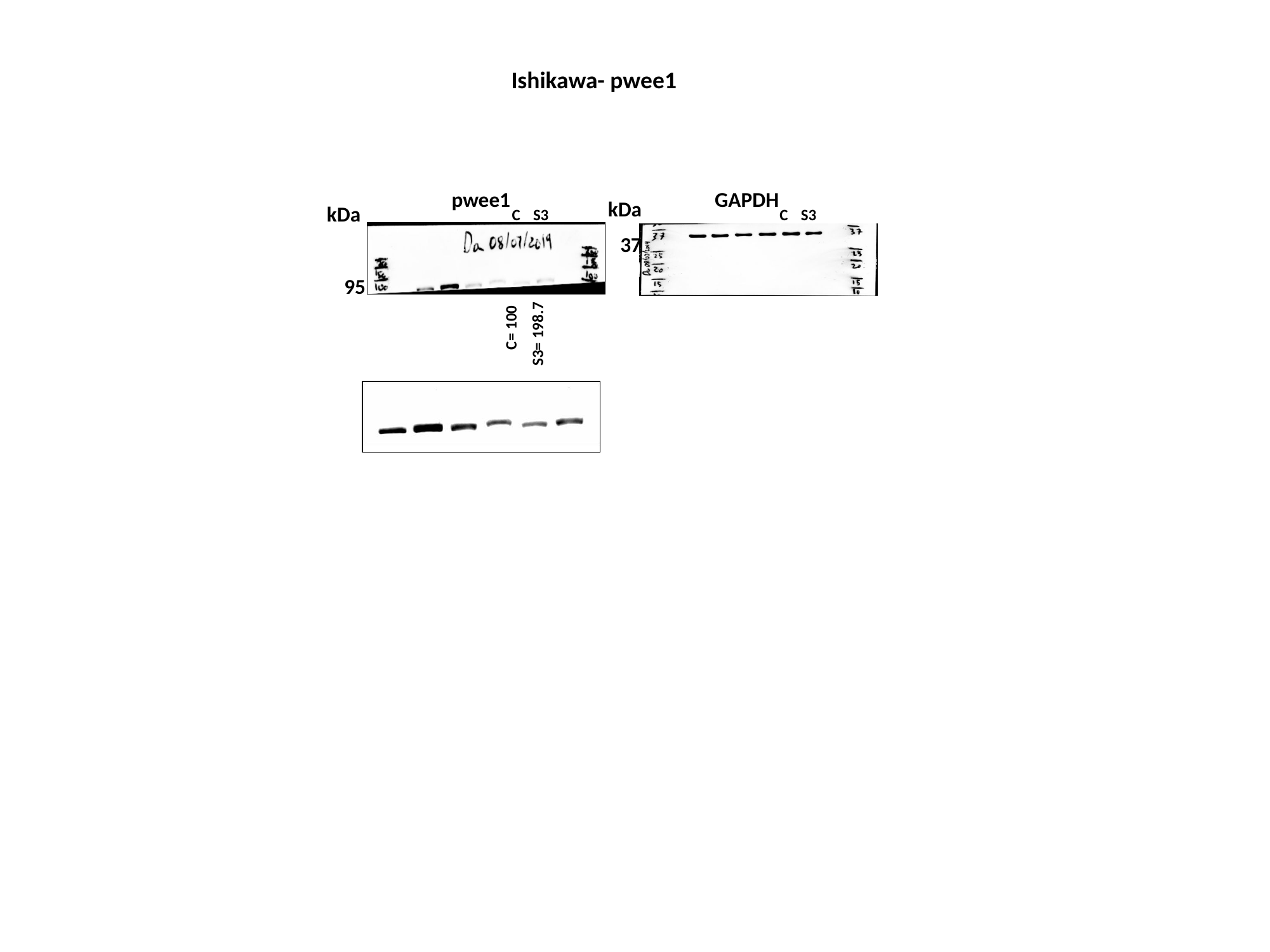

Ishikawa- pwee1
pwee1
GAPDH
kDa
kDa
S3
C
S3
C
37
95
C= 100
S3= 198.7

## Slide 53
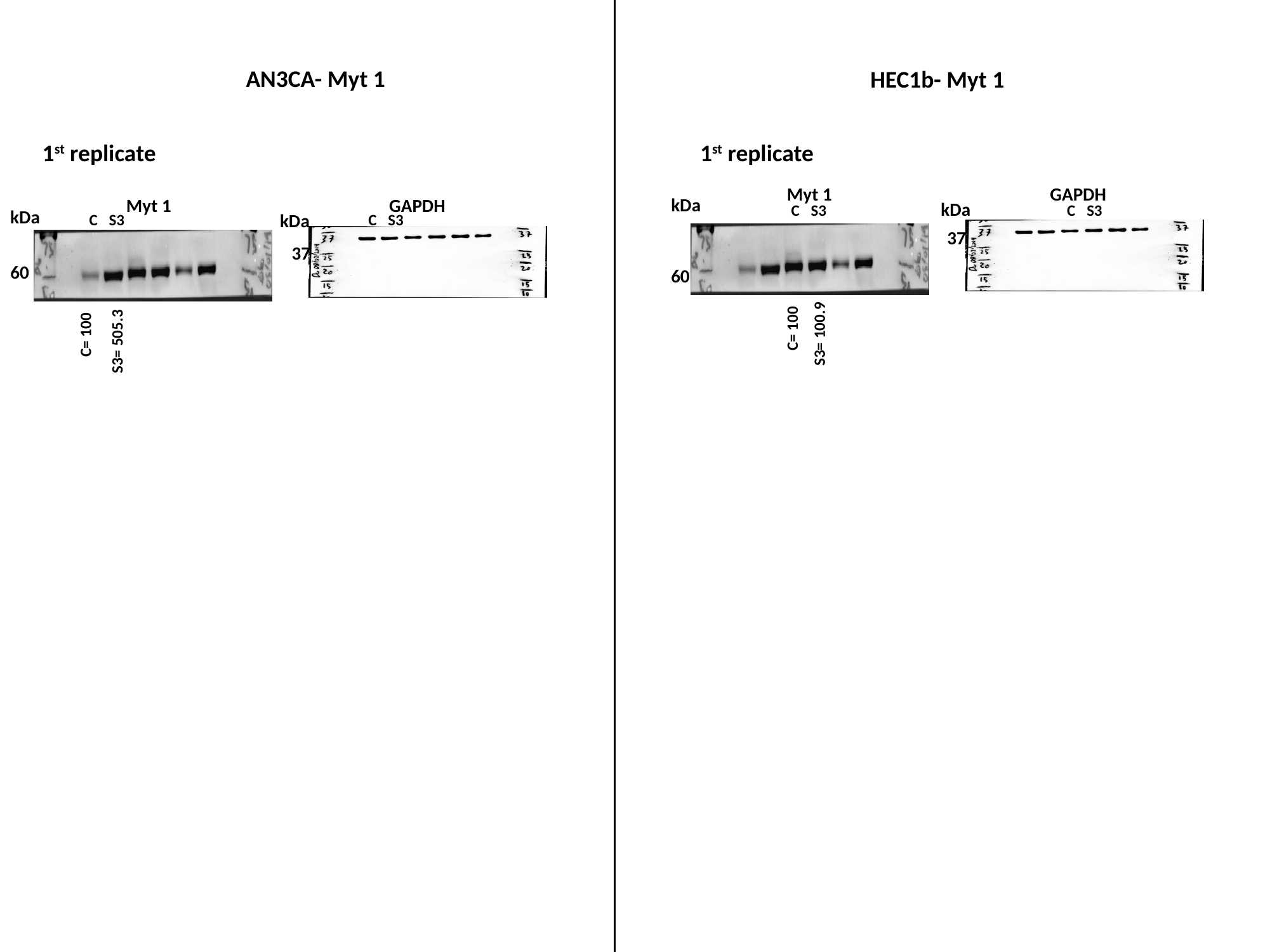

AN3CA- Myt 1
HEC1b- Myt 1
1st replicate
1st replicate
Myt 1
GAPDH
kDa
Myt 1
GAPDH
kDa
C
S3
C
S3
kDa
kDa
C
S3
C
S3
37
37
60
60
C= 100
S3= 100.9
C= 100
S3= 505.3

## Slide 54
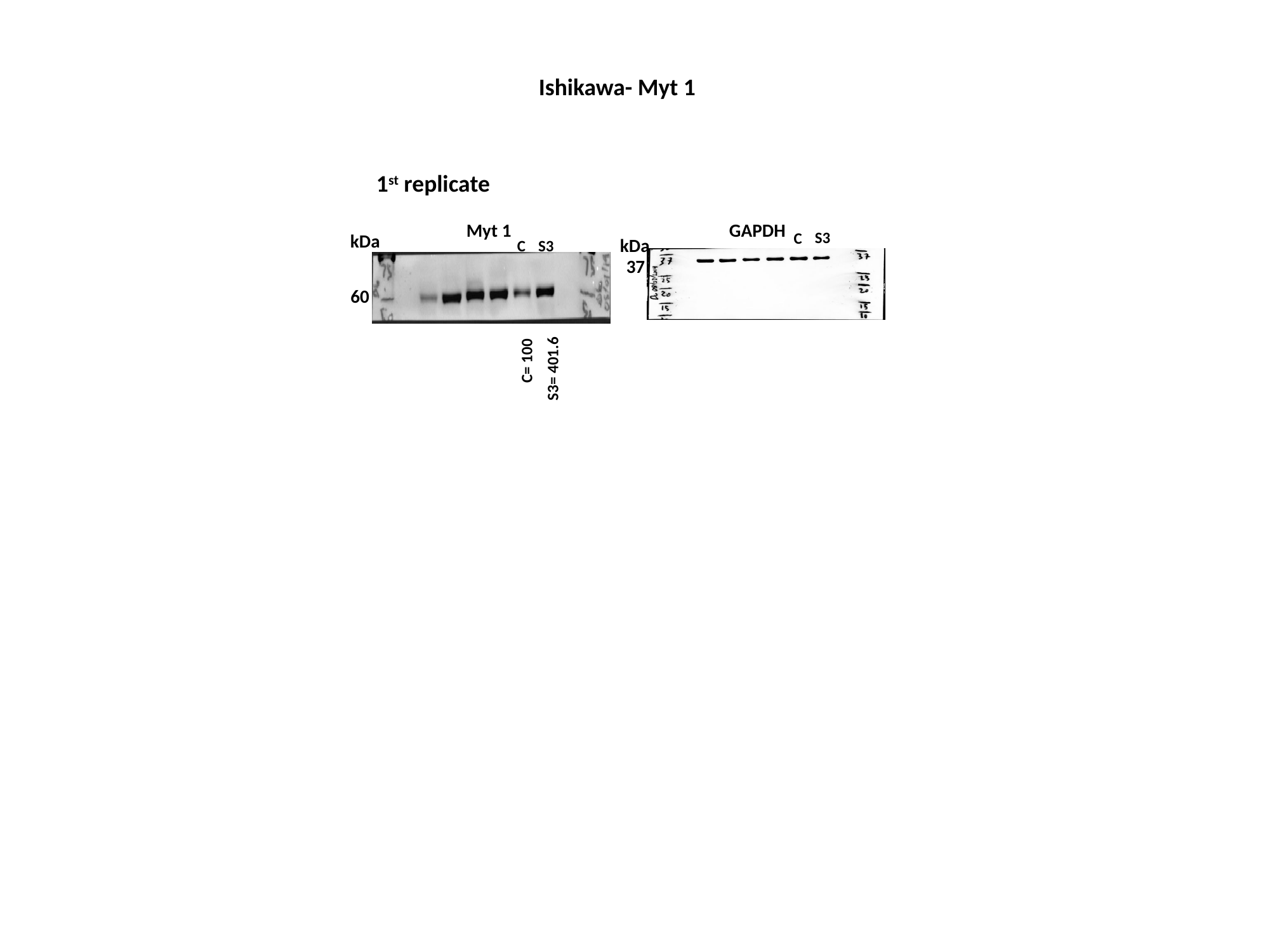

Ishikawa- Myt 1
1st replicate
Myt 1
GAPDH
S3
C
kDa
kDa
C
S3
37
60
C= 100
S3= 401.6

## Slide 55
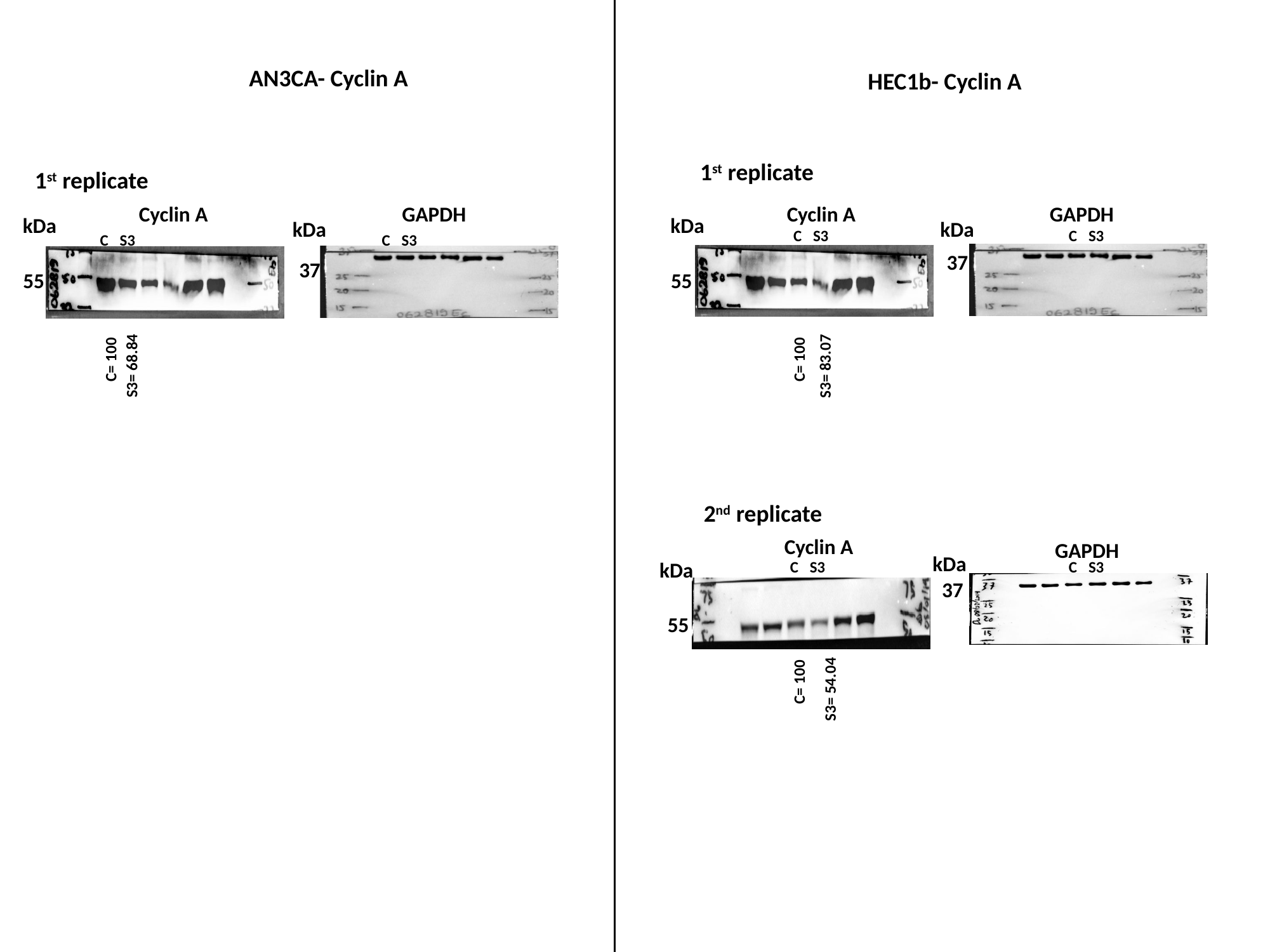

AN3CA- Cyclin A
HEC1b- Cyclin A
1st replicate
1st replicate
Cyclin A
GAPDH
Cyclin A
GAPDH
kDa
kDa
kDa
kDa
C
S3
C
S3
C
S3
C
S3
37
37
55
55
C= 100
C= 100
S3= 68.84
S3= 83.07
2nd replicate
Cyclin A
GAPDH
kDa
kDa
C
S3
C
S3
37
55
C= 100
S3= 54.04

## Slide 56
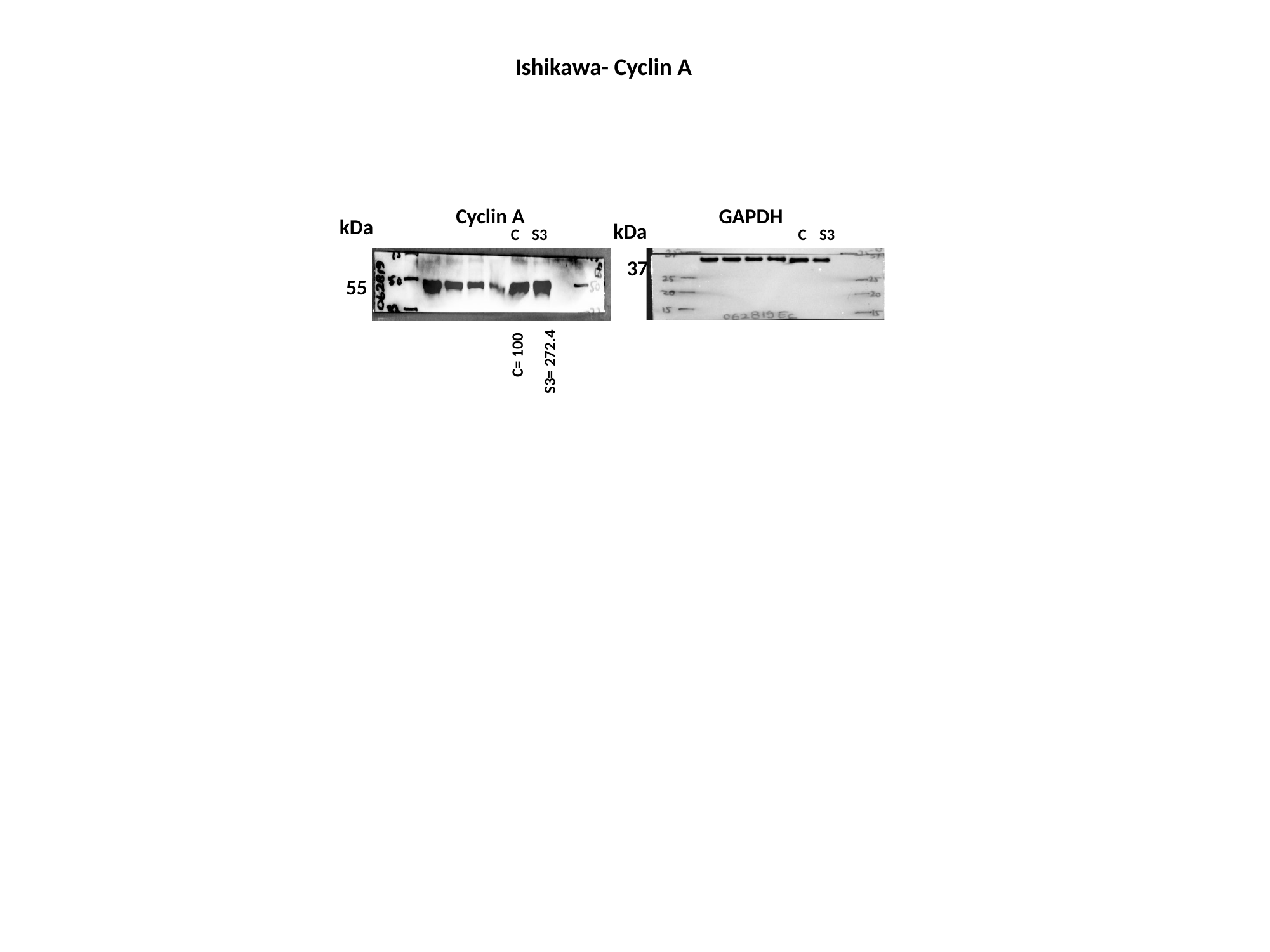

Ishikawa- Cyclin A
Cyclin A
GAPDH
kDa
kDa
S3
C
S3
C
37
55
C= 100
S3= 272.4

## Slide 57
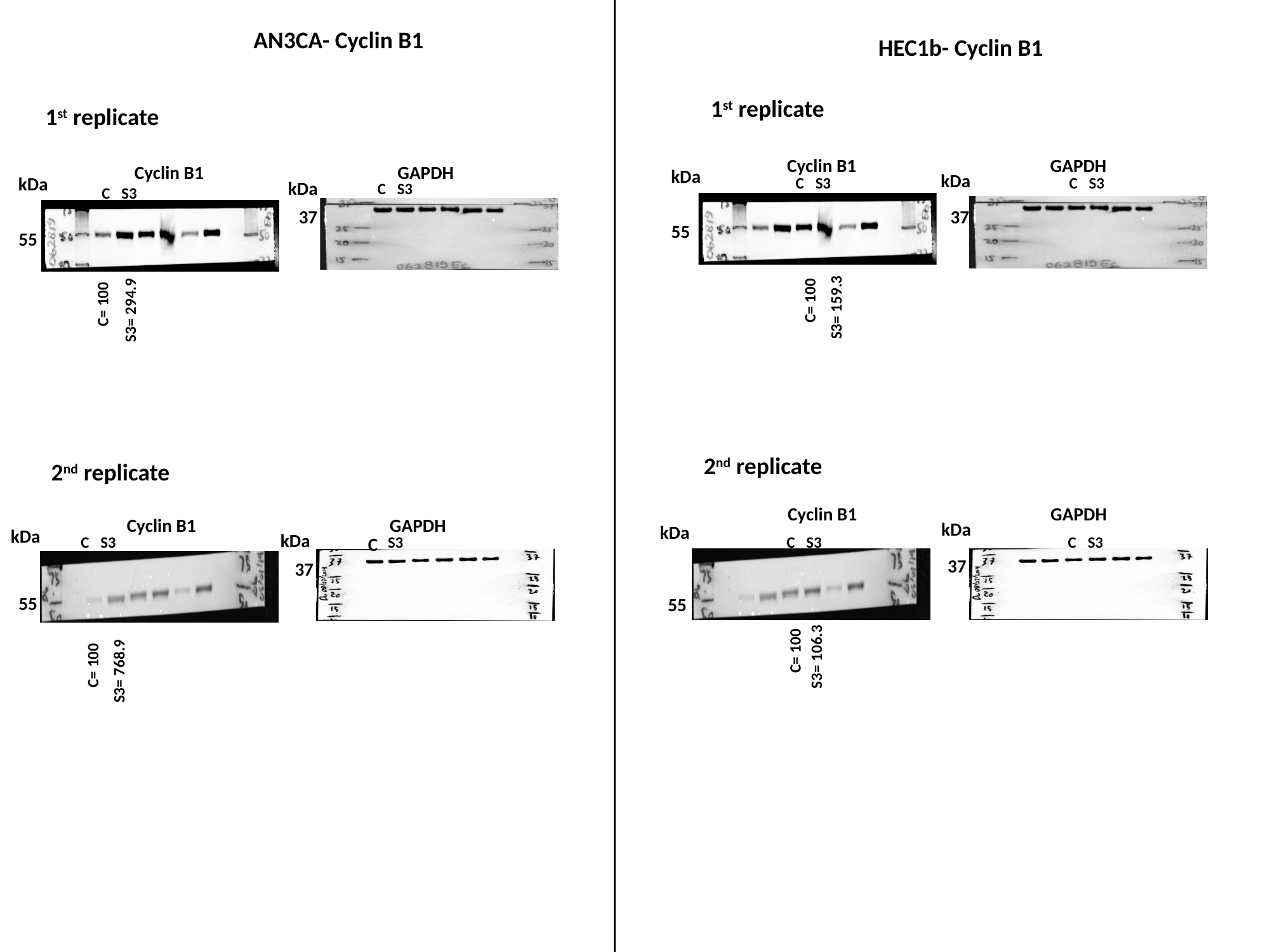

AN3CA- Cyclin B1
HEC1b- Cyclin B1
1st replicate
1st replicate
Cyclin B1
GAPDH
Cyclin B1
GAPDH
kDa
kDa
kDa
C
S3
C
S3
kDa
C
S3
C
S3
37
37
55
55
C= 100
C= 100
S3= 159.3
S3= 294.9
2nd replicate
2nd replicate
Cyclin B1
GAPDH
Cyclin B1
GAPDH
kDa
kDa
kDa
kDa
C
S3
C
S3
C
S3
C
S3
37
37
55
55
C= 100
S3= 106.3
C= 100
S3= 768.9

## Slide 58
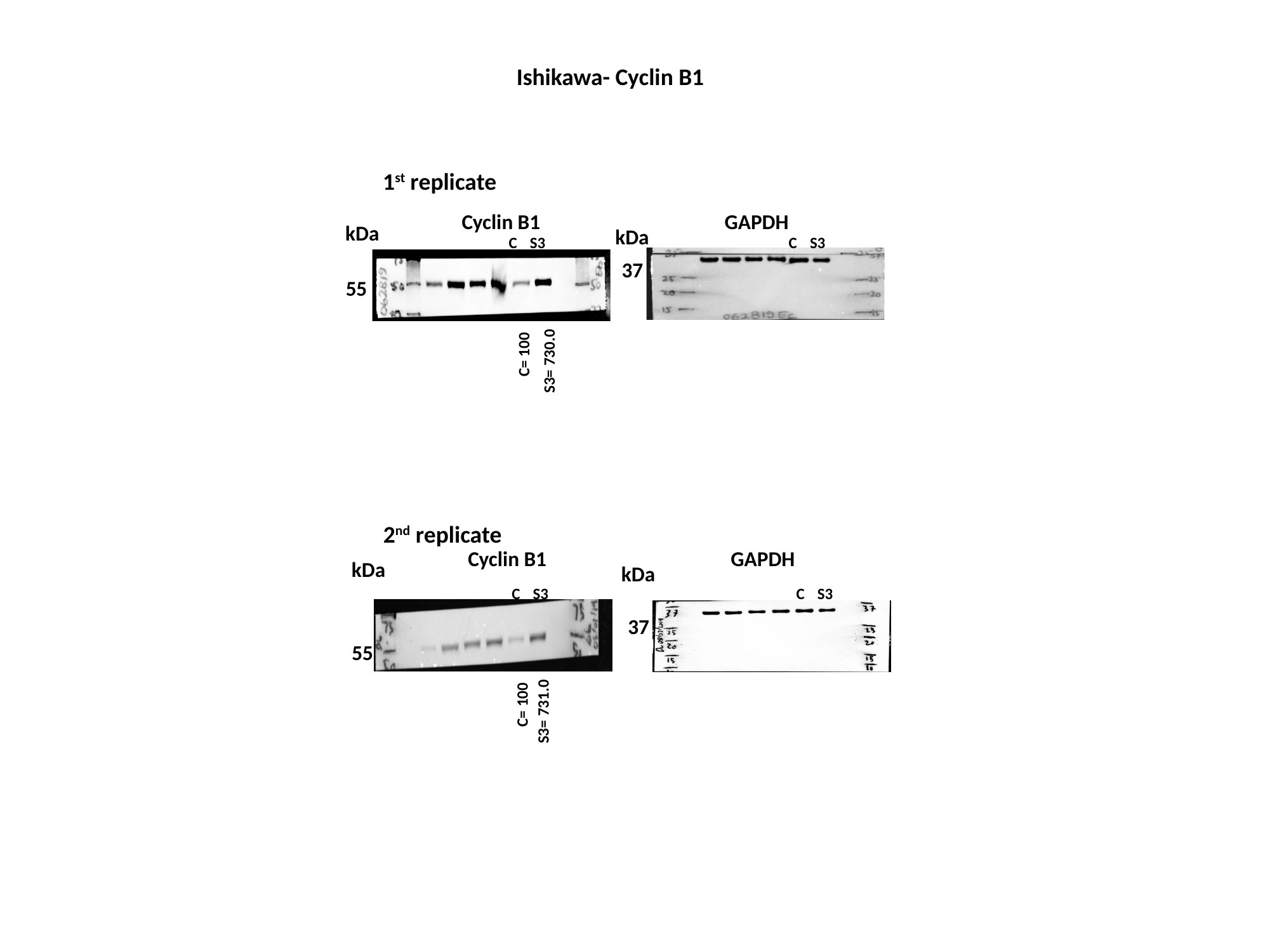

Ishikawa- Cyclin B1
1st replicate
Cyclin B1
GAPDH
kDa
kDa
S3
C
S3
C
37
55
C= 100
S3= 730.0
2nd replicate
Cyclin B1
GAPDH
kDa
kDa
S3
C
S3
C
37
55
C= 100
S3= 731.0

## Slide 59
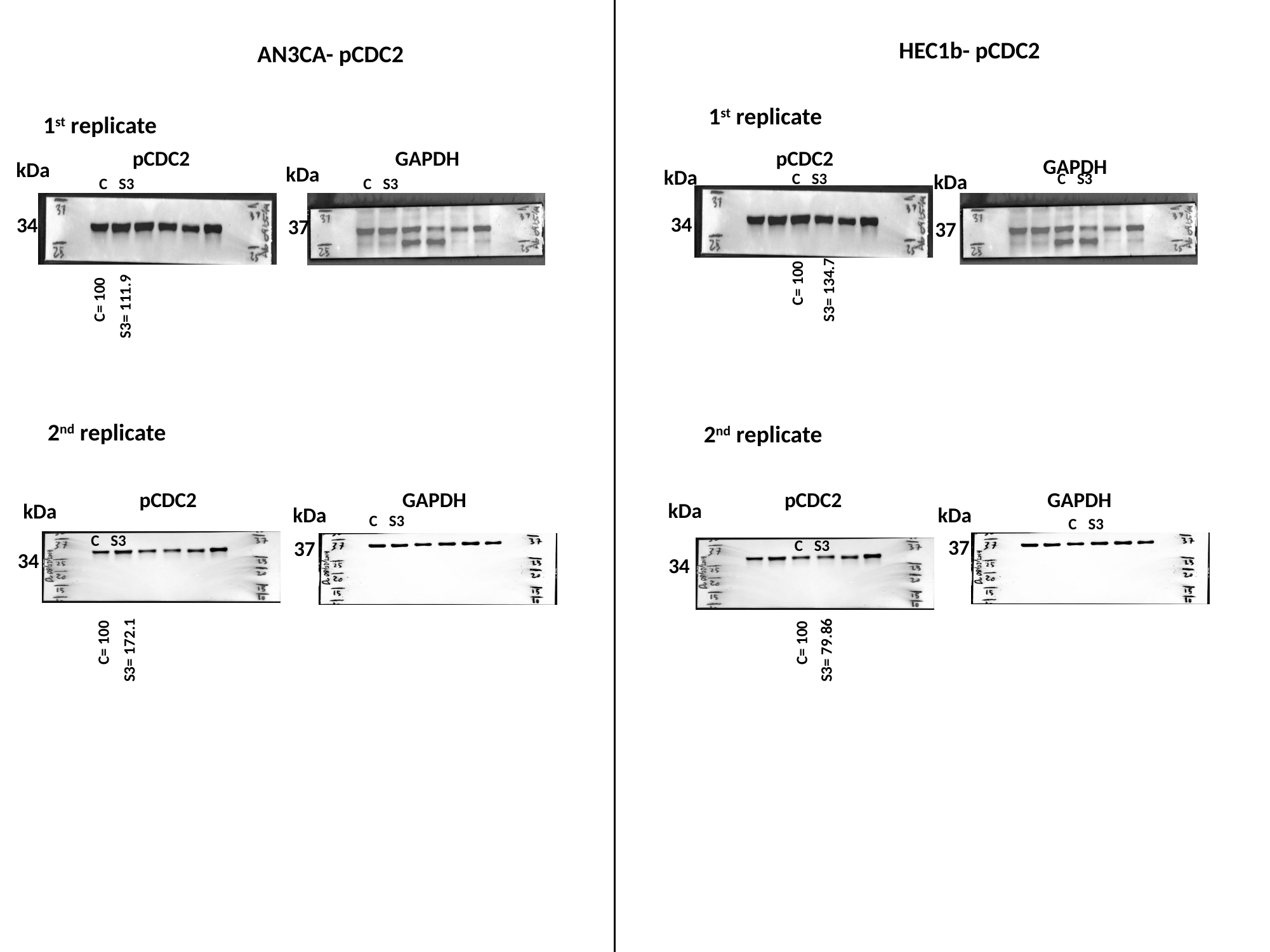

HEC1b- pCDC2
AN3CA- pCDC2
1st replicate
1st replicate
pCDC2
pCDC2
GAPDH
GAPDH
kDa
kDa
kDa
kDa
C
S3
C
S3
C
S3
C
S3
34
34
37
37
C= 100
S3= 134.7
C= 100
S3= 111.9
2nd replicate
2nd replicate
pCDC2
GAPDH
pCDC2
GAPDH
kDa
kDa
kDa
kDa
C
S3
C
S3
C
S3
37
37
C
S3
34
34
C= 100
C= 100
S3= 172.1
S3= 79.86

## Slide 60
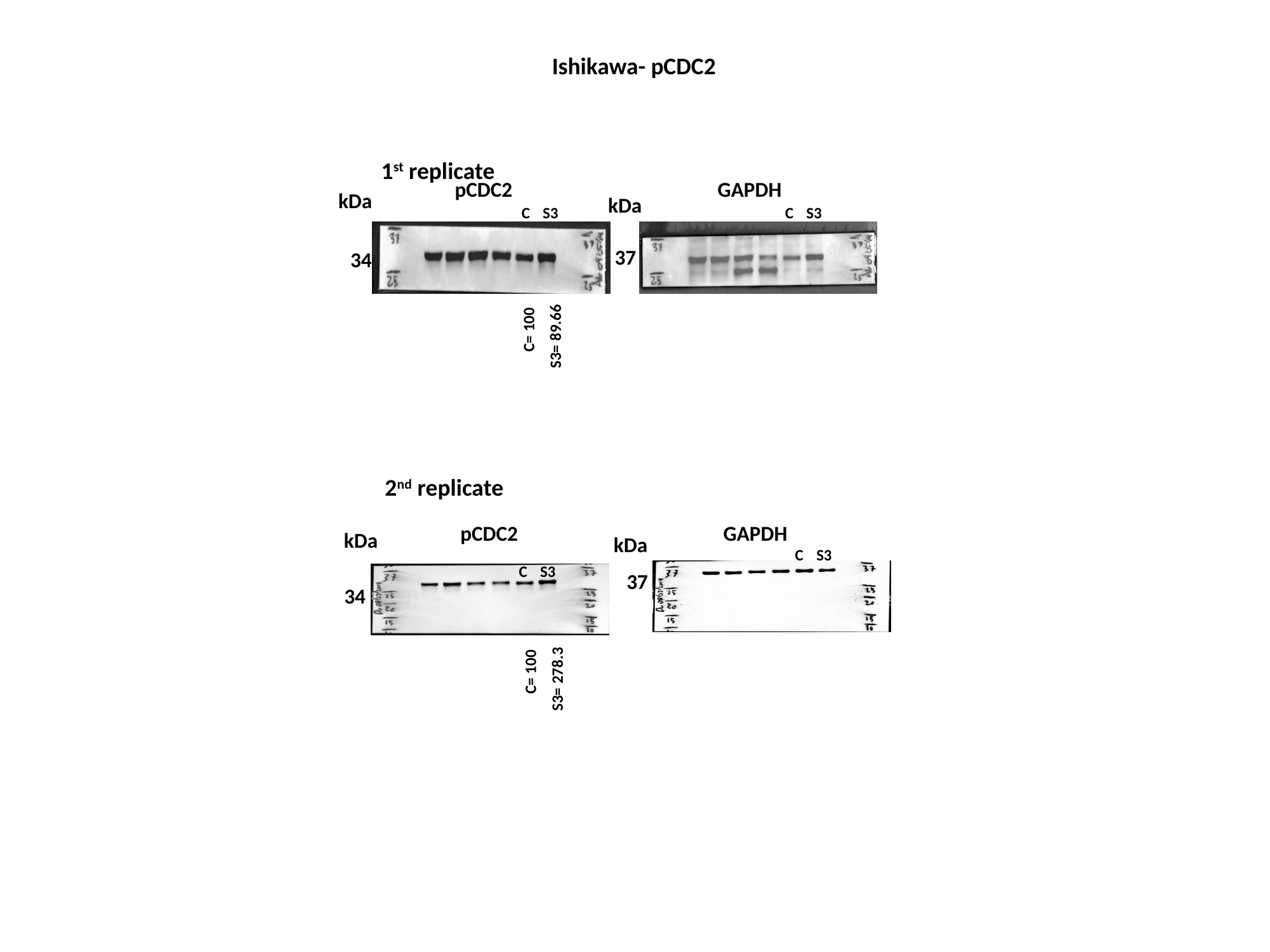

Ishikawa- pCDC2
1st replicate
pCDC2
GAPDH
kDa
kDa
S3
C
S3
C
37
34
C= 100
S3= 89.66
2nd replicate
pCDC2
GAPDH
kDa
kDa
S3
C
S3
C
37
34
C= 100
S3= 278.3

## Slide 61
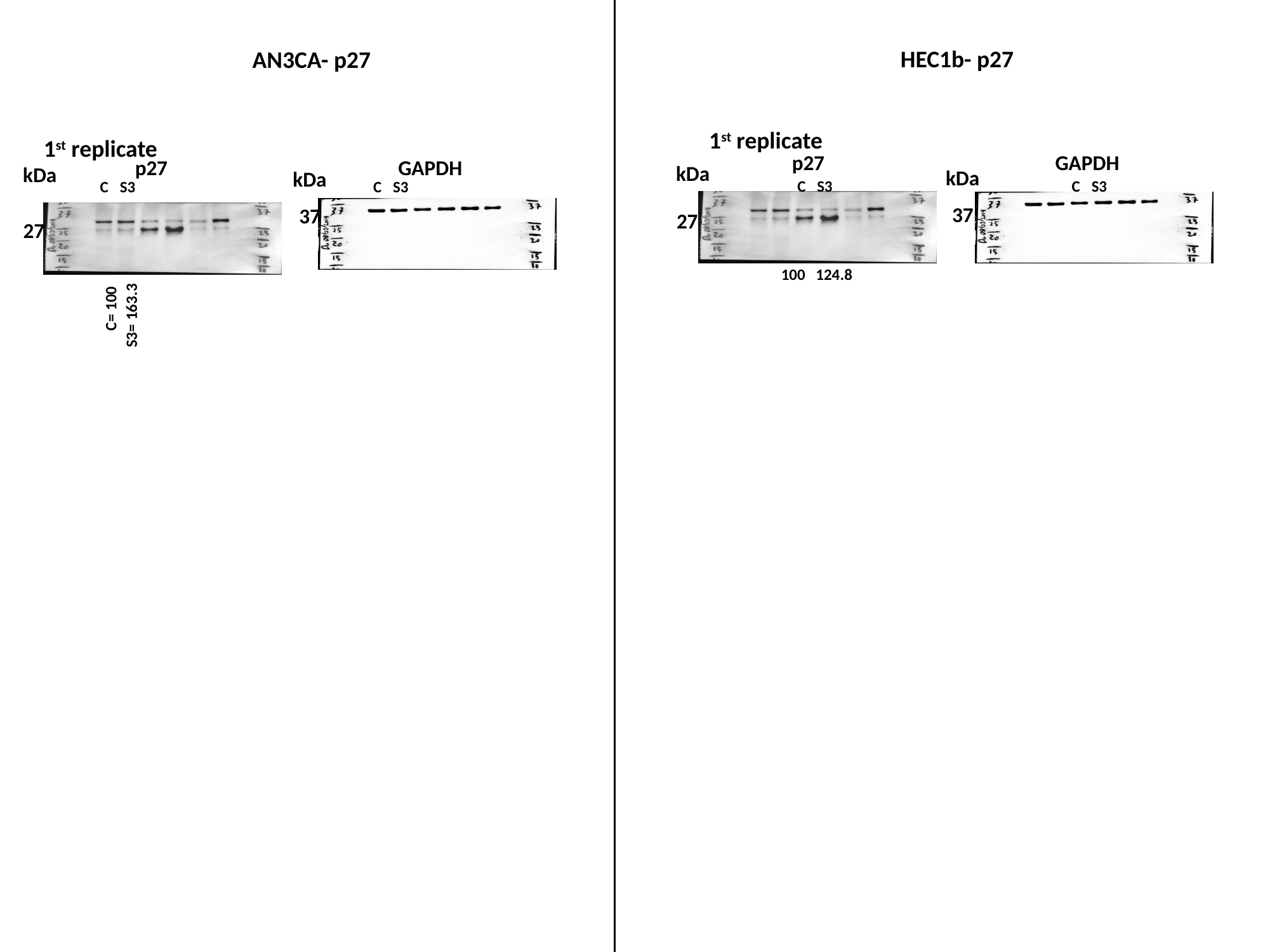

HEC1b- p27
AN3CA- p27
1st replicate
1st replicate
p27
GAPDH
p27
GAPDH
kDa
kDa
kDa
kDa
C
S3
C
S3
C
S3
C
S3
37
37
27
27
C
S3
C
S3
100
124.8
C= 100
S3= 163.3

## Slide 62
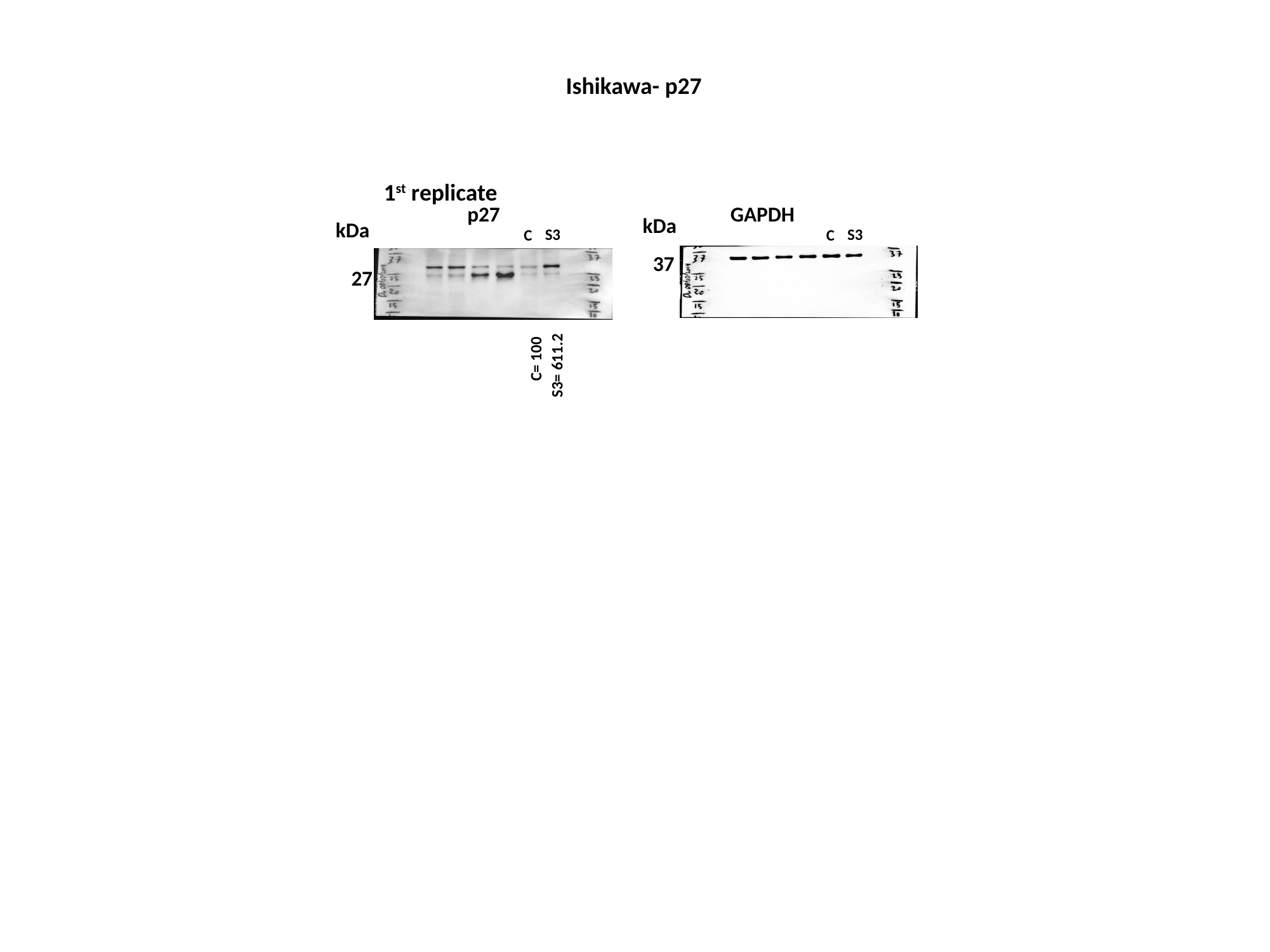

Ishikawa- p27
1st replicate
p27
GAPDH
kDa
kDa
S3
C
S3
C
37
27
C= 100
S3= 611.2

## Slide 63
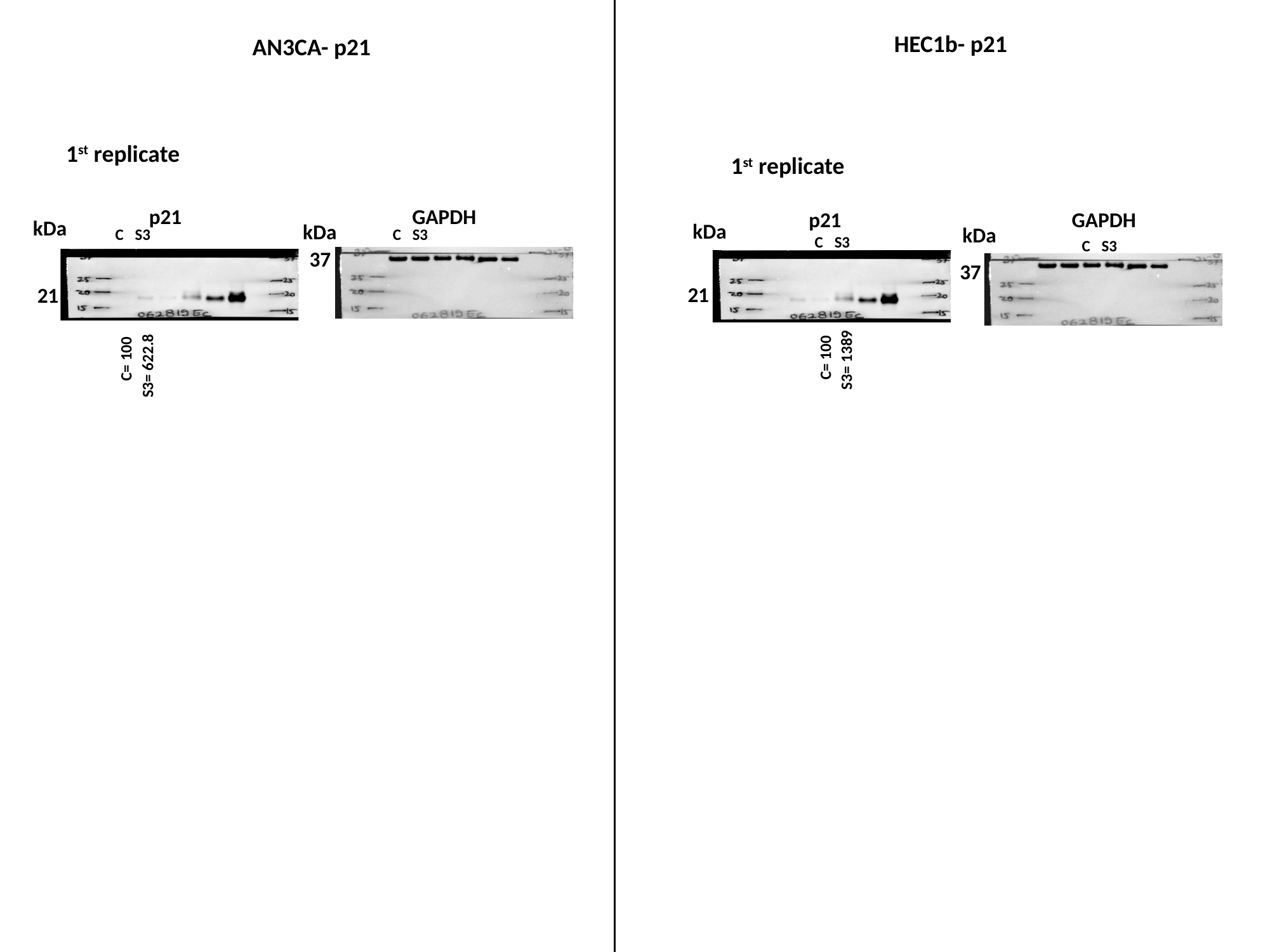

HEC1b- p21
AN3CA- p21
1st replicate
1st replicate
p21
GAPDH
p21
GAPDH
kDa
kDa
kDa
kDa
C
S3
C
S3
C
S3
C
S3
37
37
C
S3
C
S3
21
21
C= 100
C= 100
S3= 1389
S3= 622.8

## Slide 64
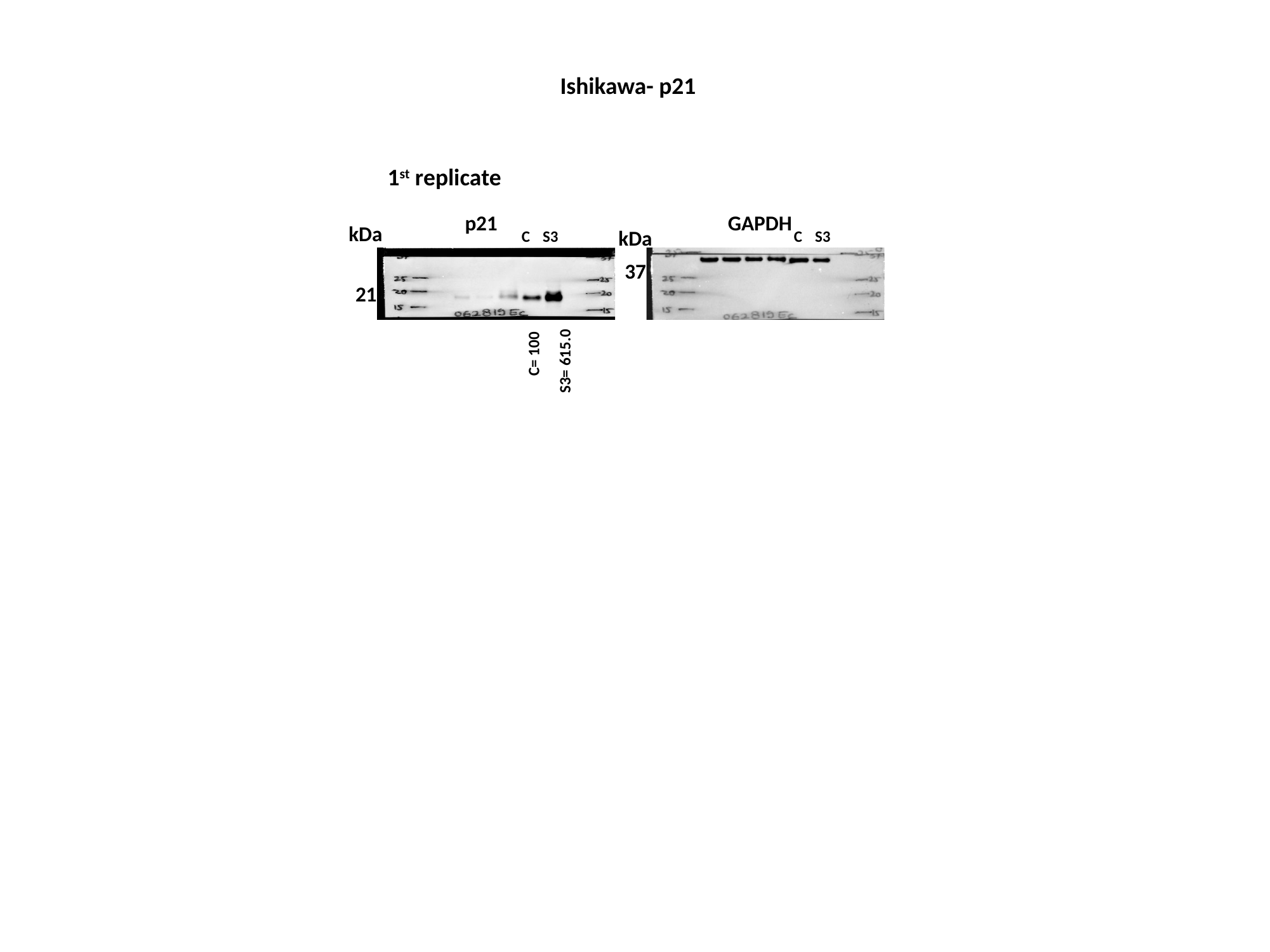

Ishikawa- p21
1st replicate
p21
GAPDH
kDa
kDa
S3
C
S3
C
37
21
C= 100
S3= 615.0

## Slide 65
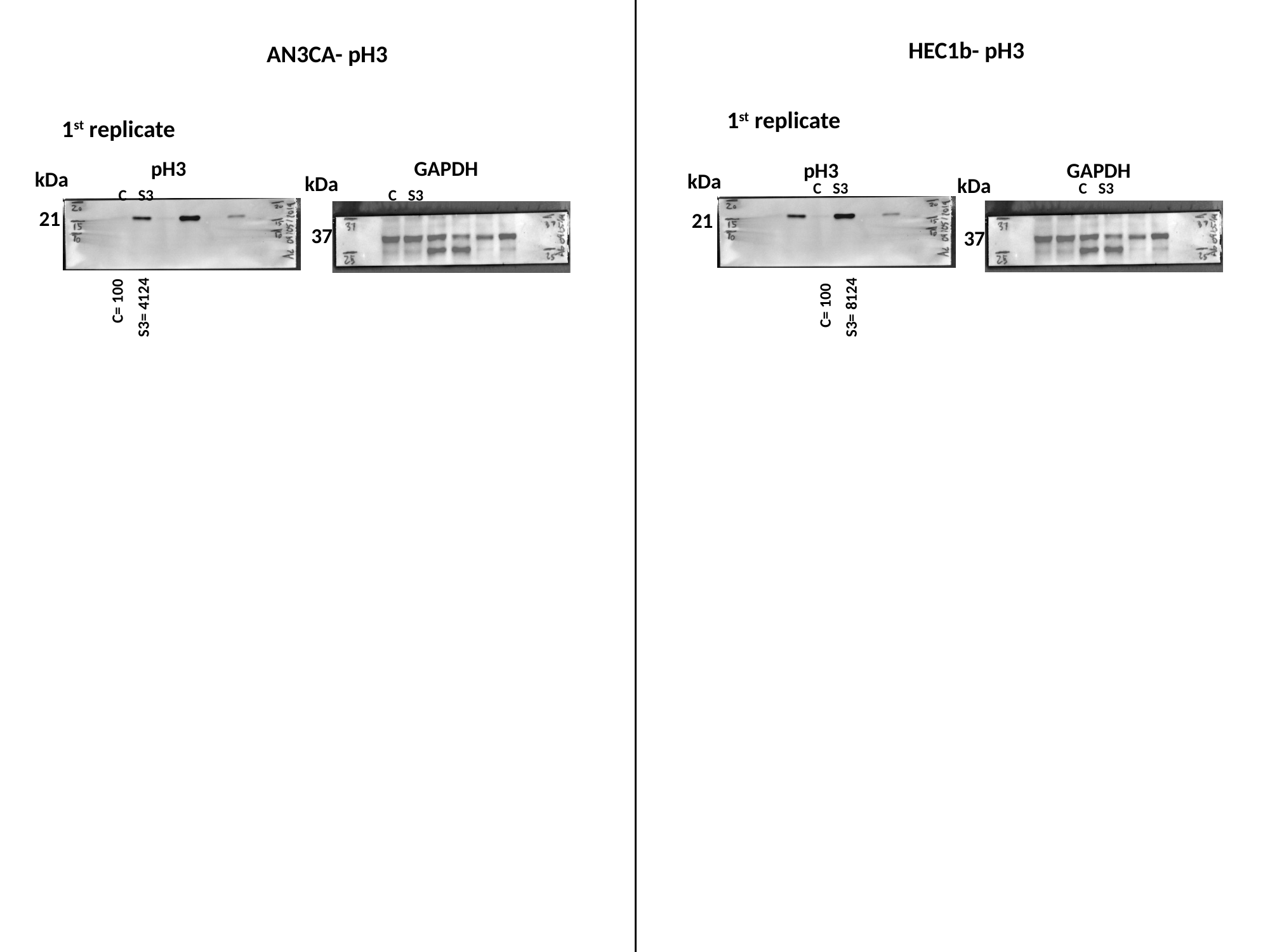

HEC1b- pH3
AN3CA- pH3
1st replicate
1st replicate
pH3
GAPDH
pH3
GAPDH
kDa
kDa
kDa
kDa
C
S3
C
S3
C
S3
C
S3
21
21
C
S3
C
S3
37
37
C= 100
C= 100
S3= 4124
S3= 8124

## Slide 66
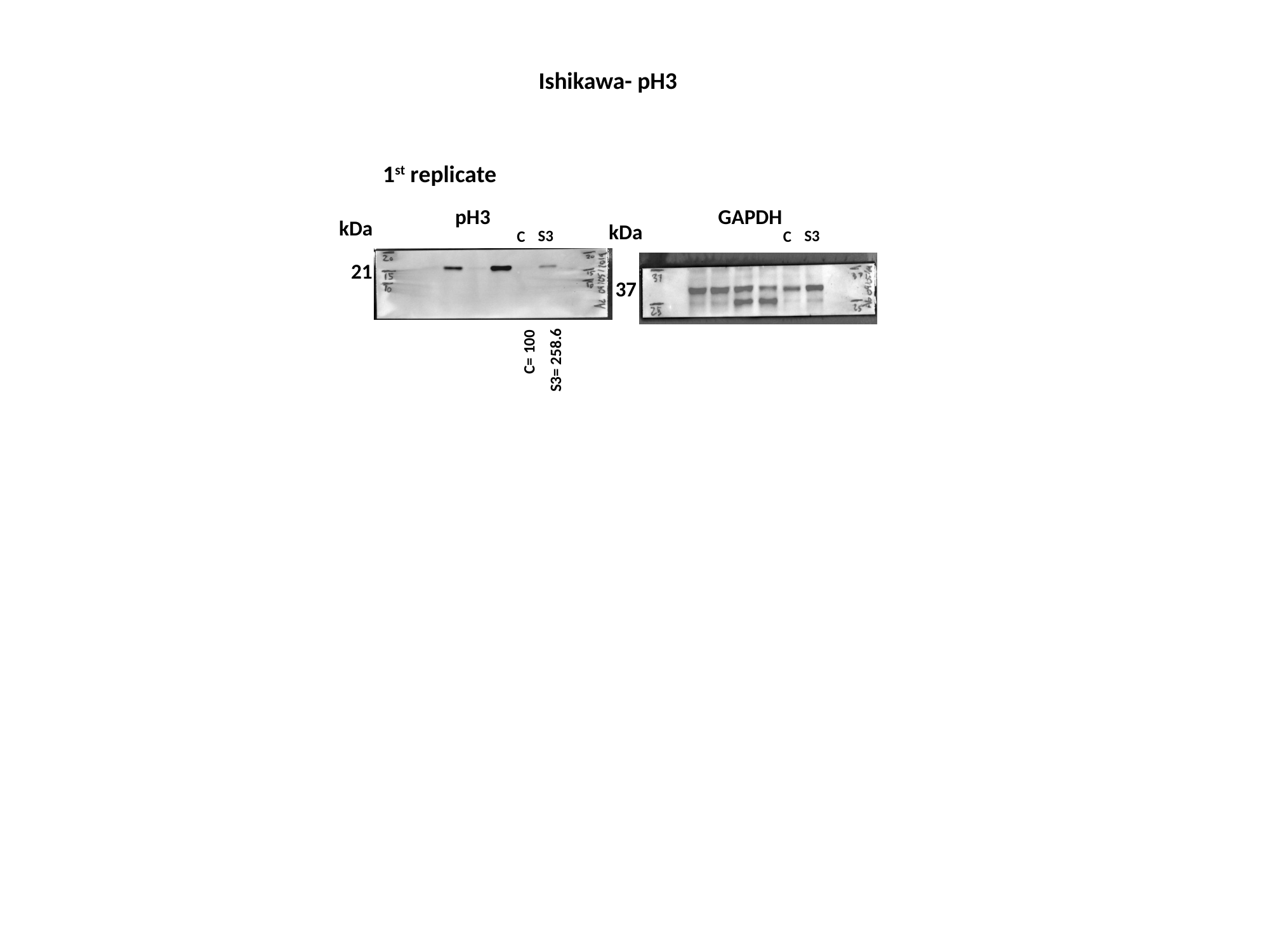

Ishikawa- pH3
1st replicate
pH3
GAPDH
kDa
kDa
S3
C
S3
C
21
37
C= 100
S3= 258.6
