# Supplementary material for: Preclinical Efficacy and Involvement of AKT, mTOR, and ERK Kinases in the Mechanism of Sulforaphane against Endometrial Cancer
Source: Cancers (Basel). 2020 May 18;12(5):1273. doi: 10.3390/cancers12051273 (PMC7281543; doi:10.3390/cancers12051273)
Supplement: Supplementary file 1 [file cancers-12-01273-s001.zip › cancers-800856 supplementary/Supplementary Table 4.rtf]

Table S4. Statistical analysis of tumor volumes in Ishikawa xenograft.
Study Week	Comparison	Mean Diff (CI)	Tukey adjusted p-value	
Day7	Control vs Pacl	-3.41 (-13.9, 7.1)	0.8176	
	Control vs SFN	-0.26 (-10.7, 10.2)	0.9999	
	Control vs SFN+Pacl	2.77 (-7.7, 13.3)	0.8912	
	Pacl vs SFN	3.14 (-7.3, 13.6)	0.8504	
	Pacl vs SFN+Pacl	6.18 (-4.3, 16.7)	0.3979	
	SFN vs SFN+Pacl	3.04 (-7.4, 13.5)	0.8628	
Day10	Control vs Pacl	1.62 (-7.5, 10.7)	0.9626	
	Control vs SFN	2.46 (-6.6, 11.5)	0.8842	
	Control vs SFN+Pacl	2.62 (-6.5, 11.7)	0.8643	
	Pacl vs SFN	0.84 (-8.2, 9.9)	0.9945	
	Pacl vs SFN+Pacl	1.00 (-8.1, 10.1)	0.9909	
	SFN vs SFN+Pacl	0.16 (-8.9, 9.2)	1.0000	
Day12	Control vs Pacl	6.13 (-3.7, 15.9)	0.3456	
	Control vs SFN	5.50 (-4.3, 15.3)	0.4403	
	Control vs SFN+Pacl	5.60 (-4.2, 15.4)	0.4235	
	Pacl vs SFN	-0.63 (-10.4, 9.2)	0.9981	
	Pacl vs SFN+Pacl	-0.52 (-10.3, 9.3)	0.9989	
	SFN vs SFN+Pacl	0.11 (-9.7, 9.9)	1.0000	
Day14	Control vs Pacl	3.51 (-20.1, 27.1)	0.9779	
	Control vs SFN	9.48 (-14.1, 33.1)	0.7031	
	Control vs SFN+Pacl	12.57 (-11.0, 36.2)	0.4874	
	Pacl vs SFN	5.97 (-17.6, 29.6)	0.9037	
	Pacl vs SFN+Pacl	9.06 (-14.6, 32.7)	0.7314	
	SFN vs SFN+Pacl	3.09 (-20.5, 26.7)	0.9847	
Day17	Control vs Pacl	6.38 (-37.3, 50.0)	0.9790	
	Control vs SFN	25.24 (-18.4, 68.9)	0.4151	
	Control vs SFN+Pacl	20.84 (-22.8, 64.5)	0.5775	
	Pacl vs SFN	18.86 (-24.8, 62.5)	0.6531	
	Pacl vs SFN+Pacl	14.46 (-29.2, 58.1)	0.8088	
	SFN vs SFN+Pacl	-4.40 (-48.0, 39.2)	0.9929	
Day19	Control vs Pacl	16.44 (-46.4, 79.3)	0.8945	
	Control vs SFN	49.58 (-13.2, 112.4)	0.1643	
	Control vs SFN+Pacl	54.14 (-8.7, 117.0)	0.1120	
	Pacl vs SFN	33.14 (-29.7, 96.0)	0.4949	
	Pacl vs SFN+Pacl	37.70 (-25.1, 100.5)	0.3825	
	SFN vs SFN+Pacl	4.55 (-58.3, 67.4)	0.9973	
Day21	Control vs Pacl	34.98 (-36.6, 106.5)	0.5589	
	Control vs SFN	55.40 (-16.2, 127.0)	0.1775	
	Control vs SFN+Pacl	71.05 (-0.5, 142.6)	0.0523	
	Pacl vs SFN	20.42 (-51.1, 92.0)	0.8681	
	Pacl vs SFN+Pacl	36.08 (-35.5, 107.6)	0.5334	
	SFN vs SFN+Pacl	15.66 (-55.9, 87.2)	0.9347	
Day24	Control vs Pacl	127.92 (-3.6, 259.5)	0.0592	
	Control vs SFN	159.70 (28.1, 291.3)	0.0122*	
	Control vs SFN+Pacl	190.96 (59.4, 322.5)	0.0021*	
	Pacl vs SFN	31.78 (-99.8, 163.3)	0.9147	
	Pacl vs SFN+Pacl	63.04 (-68.5, 194.6)	0.5748	
	SFN vs SFN+Pacl	31.26 (-100.3, 162.8)	0.9183	
Day26	Control vs Pacl	192.79 (-11.2, 396.8)	0.0698	
	Control vs SFN	259.34 (55.3, 463.4)	0.0081*	
	Control vs SFN+Pacl	312.22 (108.2, 516.3)	0.0012*	
	Pacl vs SFN	66.55 (-137.5, 270.6)	0.8160	
	Pacl vs SFN+Pacl	119.43 (-84.6, 323.5)	0.4044	
	SFN vs SFN+Pacl	52.88 (-151.1, 256.9)	0.8971	
Day28	Control vs Pacl	304.31 (27.8, 580.8)	0.0263*	
	Control vs SFN	425.06 (148.6, 701.5)	0.0011*	
	Control vs SFN+Pacl	488.36 (211.9, 764.8)	0.0002*	
	Pacl vs SFN	120.74 (-155.7, 397.2)	0.6455	
	Pacl vs SFN+Pacl	184.05 (-92.4, 460.5)	0.2933	
	SFN vs SFN+Pacl	63.31 (-213.2, 339.8)	0.9261	
Day31	Control vs Pacl	464.59 (-31.2, 960.4)	0.0730	
	Control vs SFN	790.90 (295.1, 1286.7)	0.0007*	
	Control vs SFN+Pacl	801.16 (305.4, 1296.9)	0.0006*	
	Pacl vs SFN	326.31 (-169.5, 822.1)	0.3028	
	Pacl vs SFN+Pacl	336.57 (-159.2, 832.3)	0.2770	
	SFN vs SFN+Pacl	10.26 (-485.5, 506.0)	0.9999	
SFN: Sulforaphane; Pacl; Paclitexel. Asterisk indicates statistically significant p-values
